# Integrative analyses of single-cell transcriptome and regulome using MAESTRO

**DOI:** 10.1186/s13059-020-02116-x

**Published:** 2020-08-07

**Authors:** Chenfei Wang, Dongqing Sun, Xin Huang, Changxin Wan, Ziyi Li, Ya Han, Qian Qin, Jingyu Fan, Xintao Qiu, Yingtian Xie, Clifford A. Meyer, Myles Brown, Ming Tang, Henry Long, Tao Liu, X. Shirley Liu

**Affiliations:** 1 Department of Data Science, Dana-Farber Cancer Institute, Harvard T.H. Chan School of Public Health, Boston, MA 02215 USA; 2 Center for Functional Cancer Epigenetics, Dana-Farber Cancer Institute, Boston, MA 02215 USA; 3 Clinical Translational Research Center, Shanghai Pulmonary Hospital, School of Life Science and Technology, Tongji University, Shanghai, 200433 China; 4 Beijing Institute of Radiation Medicine, Beijing, 100850 China; 5 Department of Medical Oncology, Dana-Farber Cancer Institute, Harvard Medical School, Boston, MA 02215 USA; 6 Department of Biostatistics and Bioinformatics, Roswell Park Comprehensive Cancer Center, Buffalo, NY 14263 USA

**Keywords:** Single-cell RNA-seq, Single-cell ATAC-seq, Computational workflow, Integrate scRNA-seq and scATAC-seq, Cell-type annotation, Predict transcriptional regulators

## Abstract

We present Model-based AnalysEs of Transcriptome and RegulOme (MAESTRO), a comprehensive open-source computational workflow (http://github.com/liulab-dfci/MAESTRO) for the integrative analyses of single-cell RNA-seq (scRNA-seq) and ATAC-seq (scATAC-seq) data from multiple platforms. MAESTRO provides functions for pre-processing, alignment, quality control, expression and chromatin accessibility quantification, clustering, differential analysis, and annotation. By modeling gene regulatory potential from chromatin accessibilities at the single-cell level, MAESTRO outperforms the existing methods for integrating the cell clusters between scRNA-seq and scATAC-seq. Furthermore, MAESTRO supports automatic cell-type annotation using predefined cell type marker genes and identifies driver regulators from differential scRNA-seq genes and scATAC-seq peaks.

## Background

Cells in a multicellular organism may display tremendous transcriptomic and epigenetic heterogeneities. Cellular identity and function are mainly determined by the genes that are regulated and expressed in the cell [1, 2]. Traditional profiling techniques for gene expression and *cis*-regulatory elements through bulk RNA-seq and ATAC-seq, respectively, are limited in deciphering the heterogeneous gene expression and regulation in complex biological systems [3, 4]. Recent advances in single-cell technologies enabled the measurements of gene expression and chromatin accessibility at a single-cell resolution using scRNA-seq and scATAC-seq [5–8]. They provided unprecedented opportunities to investigate the complex gene regulation mechanisms underlying immune response [9, 10], brain function [11], tumor heterogeneity [12], and developmental plasticity [13, 14]. However, these technologies also generate large volumes of data, which pose significant computational challenges.

Although many methods have been developed to analyze single-cell data, several computational challenges remain to be resolved [15]. First, pre-processing of scRNA-seq and scATAC-seq datasets can be a complicated task due to the diverse single-cell indexing strategies. Current workflows that support pre-processing single-cell datasets from sequencing files are often designed for specific technologies or platforms, such as Cellranger suites [16] for the 10X Genomics dataset, snapATAC [17] for 10X Genomics scATAC-seq analysis, and Dr.seq2 [18] for droplet-based technologies. Second, most of the tools for single-cell analysis focus on specific analytical problems instead of providing an end-to-end workflow from alignment to post-clustering annotations. For example, SC3 [19] and SNNCliq [20] are developed for scRNA-seq clustering, scde [21] and MAST [22] for differential expression, scABC [23] and cisTopic [24] for scATAC-seq clustering, and chromVAR [25] and Cicero [26] for quantifying the chromatin accessibility at transcription regulator and gene level, respectively. Even pipelines with multiple functions, such as Monocle [27], Seurat [28], and Scanpy [29], lack the function to identify transcription regulators, which is crucial to understand the gene regulatory networks that regulate cell state transition and lineage determination [25, 30]. Lastly, current multimodal single-cell technologies, such as scRNA-seq and scATAC-seq, enable analyses of cellular states and interactions from a holistic view [31]. However, most of the existing methods only concentrate on one modality. Therefore, workflows supporting multiple modalities and their integration are in great need.

In this study, we present the Model-based Analyses of Transcriptome and RegulOme (MAESTRO) workflow to overcome the computational challenges in analyzing scRNA-seq and scATAC-seq datasets. First, MAESTRO provides comprehensive functions for pre-processing, alignment, quality control, and expression- and accessibility quantification for scRNA-seq and scATAC-seq data from multiple platforms. Second, MAESTRO employs the best practices for cell clustering and differential analysis and allows automatic cell-type annotation and transcriptional regulator inference for both scRNA-seq and scATAC-seq dataset. Finally, by modeling the chromatin accessibility at the gene level, MAESTRO outperforms the existing methods in the integrative analysis of scRNA-seq and scATAC-seq data. To demonstrate the utility of MAESTRO, we applied it to scRNA-seq and scATAC-seq profiles of bone marrow-derived mononuclear cells (BMMCs) from a chronic lymphocytic leukemia (CLL) patient and a healthy donor. We identified distinct cell-type compositions and transcriptional regulators in the bone marrow microenvironment between the CLL patient and healthy donor and demonstrated robust transcriptional regulator predictions supported by both scRNA-seq and scATAC-seq data. MAESTRO provides user-friendly and scalable features to analyze and integrate scRNA-seq and scATAC-seq data, and its continued maintenance and update promise to be of great utility to the gene regulation community.

## Results

### Comprehensive features of the MAESTRO workflow

**Fig. 1:**
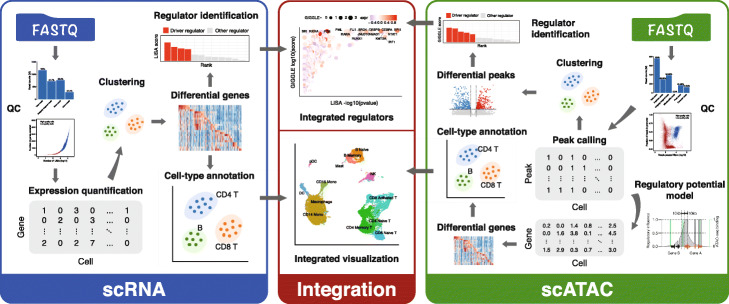
An overview of the MAESTRO workflow. Starting from fastq files, MAESTRO performs pre-processing, alignment, quality control, expression index for scRNA-seq, peak calling for scATAC-seq, clustering, differential analysis, cell-type annotation, and transcription factor identification analysis. If scRNA-seq and scATAC-seq from the same experiment are given, MAESTRO can perform the integration analysis and annotate the integrated clusters

MAESTRO workflow includes three main modules, for analyzing scRNA-seq, scATAC-seq, and integrating the two (Fig. 1). Most other single-cell analysis tools start from the processed datasets, while MAESTRO supports input from fastq files for a wide variety of single-cell sequencing-based platforms including Smart-seq for scRNA-seq [32], microfluidic-based scATAC-seq [7], and barcode-based systems such as 10X Genomics [16], drop-seq [5, 6], sci-ATAC-seq [33], and d-sci-ATAC-seq. MAESTRO also enables the most comprehensive post-alignment analysis functions compared to the existing workflows and provides a full solution for scRNA-seq and scATAC-seq analyses (Table 1 and Additional file 1: Table S1). In addition, we optimized MAESTRO to achieve high computational efficiency and scalability in scATAC-seq data analysis. We benchmarked the running time and memory usage using publicly available scATAC-seq datasets on peripheral blood mononuclear cells (PBMC, 10k cells) and basal cell carcinoma (BCC, 38k cells). MAESTRO shows superior performance in terms of CPU time and memory efficiency. It can handle large datasets of 40k cells while several other tools crashed due to memory overflow on our computer server with 380 GB total memory (Additional file 2: Table S2).

**Table 1:** Comprehensive features of MAESTRO compared to other single-cell tools

Methods	Multiple technologies (e.g., scRNA-seq, scATAC-seq)	Multiple platforms (e.g., 10X Genomics, sci-ATAC-seq)	Alignment	Bulk level QC	Single-cell QC	Expression index and peak calling	Normalization	Clustering	Differential analysis	Cell-type annotation	Regulator annotation	Integrated analysis between multiple technologies
SC3 [19]		✓					✓	✓				
SNN-cliq [20]		✓					✓	✓				
MAST [22]		✓							✓			
scde [21]		✓							✓			
Monocle [27]		✓			✓		✓	✓	✓			
Pagoda [34]		✓			✓		✓	✓	✓			
Scanpy [29]		✓			✓		✓	✓	✓			
Seurat [28]	✓	✓			✓		✓	✓	✓			✓
scABC [23]		✓					✓	✓				
CisTopic [24]		✓					✓	✓			✓	
chromVAR [25]		✓					✓	✓			✓	
Cicero [26]		✓					✓	✓	✓			
Cellranger [16]	✓		✓	✓	✓	✓	✓	✓	✓			
Dr.seq2 [18]	✓		✓	✓	✓	✓	✓	✓	✓			
snapATAC [17]		✓	✓	✓	✓	✓	✓	✓	✓		✓	
MAESTRO	✓	✓	✓	✓	✓	✓	✓	✓	✓	✓	✓	✓

We implemented the MAESTRO using the Snakemake workflow management system [35], which brings three advantages. First, MAESTRO utilizes Snakemake to deploy and parallelize jobs on most computing platforms from high-performance servers, clusters, to the cloud. Second, MAESTRO retrieves job descriptions and parameters through the Snakemake configuration files, so the pipeline can be easily customized for data from different technologies. Last, the Snakemake workflow keeps track of the parameters and log files in each step, so it is easy to reproduce the result or fine-tune the MAESTRO pipeline. Additionally, we provide all the MAESTRO components under the Conda environment [36] allowing streamlined MAESTRO installation with a single command.

### Multiple levels of quality control

MAESTRO performs quality control (QC) at two levels. The bulk-level QC evaluates the sample quality by considering all the cells together, while the single cell-level QC evaluates individual cells and filters low-quality ones from downstream analysis.

**Fig. 2:**
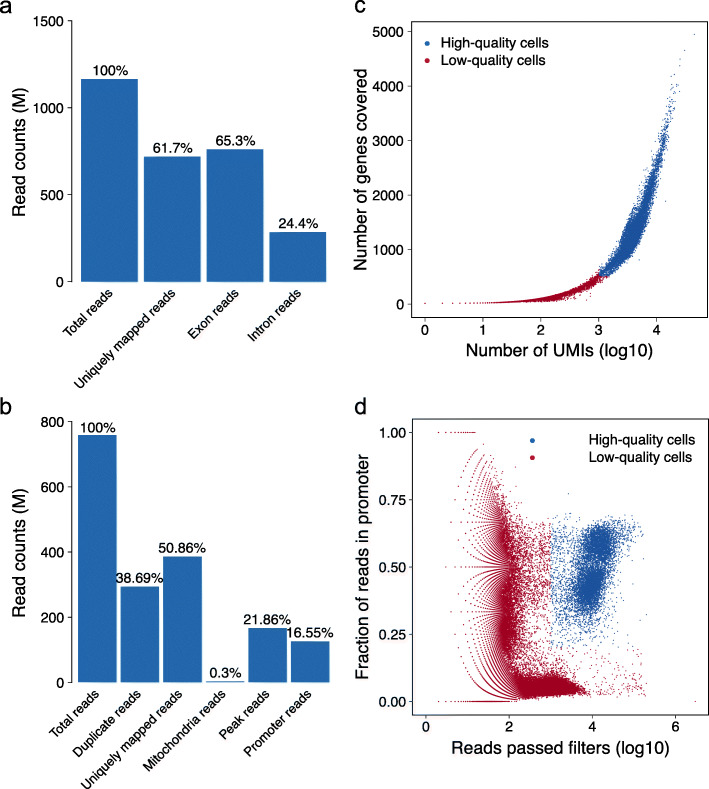
Pre-processing and quality control using MAESTRO. **a** Mapping summary of human PBMC scRNA-seq (12k cells) dataset. **b** Mapping summary of human PBMC scATAC-seq (10k cells) dataset. **c** Cell filtering plot of PBMC scRNA-seq. The *x*-axis represents the number of unique reads/UMIs present in each cell, and the *y*-axis represents the number of genes covered in each cell. **d** Cell filtering plot of PBMC scATAC-seq. The *x*-axis represents the number of unique reads present in each cell, and the *y*-axis represents the fraction of reads in promoter regions (defined as 2 kb up/downstream of TSS)

We demonstrated the bulk QC metrics on 10X Genomics human PBMC scRNA-seq (12k cells) and scATAC-seq (10k cells) datasets in Fig. 2. For scRNA-seq, MAESTRO checks read mappability, distribution of reads in coding regions (CDS) and intronic regions, and coverage of reads over a gene body (Fig. 2a and Additional file 3: Fig. S1a-d, see the “Methods” section for details). For scATAC-seq, MAESTRO evaluates read mappability, duplicated reads percentage, fraction of reads mapped to mitochondria genes and peak regions, and fragment size distribution (Fig. 2b and Additional file 3: Fig. S1e). MAESTRO also provides the normal ranges of bulk-level QC metrics in HTML output for users to better evaluate their sample quality.

The single-cell level QC in MAESTRO aims to remove the low-quality cells in single-cell experiments, which might arise from incompletely captured or dead cells and empty or overloaded droplets [5, 6]. When processing scRNA-seq data, MAESTRO filters cells with few uniquely sequenced reads or UMIs or with few expressed genes (Fig. 2c) to only keep high-quality cells with enough sequencing depth and gene detection rates for downstream analyses. For scATAC-seq, MAESTRO evaluates each cell by the number of unique reads detected in the cell, as well as the fraction of reads in promoter regions as a proxy of signal-to-noise ratio (Fig. 2d). The high-quality and low-quality cells have distinct distributions when plotted using these metrics, thus can be clearly separated efficiently (Fig. 2c, d).

### Clustering and gene activity modeling

**Fig. 3:**
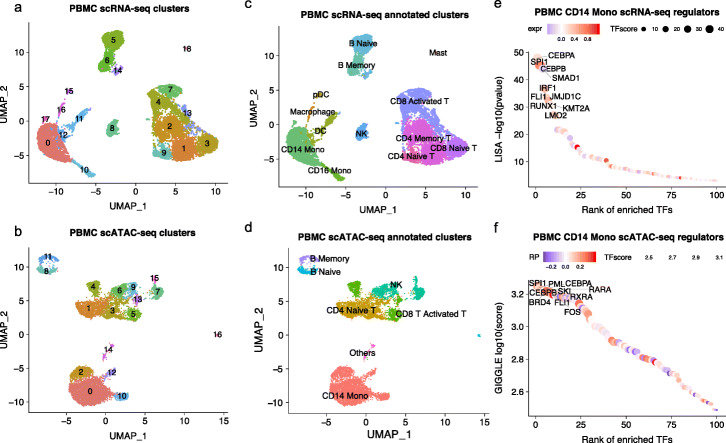
Clustering, cell-type, and transcriptional regulator annotation using MAESTRO. **a** UMAP visualization of human PBMC scRNA-seq (12k cells) clusters. Colors represent the different clusters with the cluster ID labeled. **b** UMAP visualization of human PBMC scATAC-seq (10k cells) clusters. Colors represent the different clusters with cluster ID labeled. **c** UMAP visualization of human PBMC scRNA-seq (12k cells) clusters. Colors represent the different cell types. The cell-type information for each cluster is annotated using the expression level of marker genes. **d** UMAP visualization of human PBMC scATAC-seq (10k cells) clusters. Colors represent the different cell types. The cell-type information for each cluster is annotated using the regulatory potential of marker genes. **e** The rank of driver transcription regulators in the CD14 monocyte cells of PBMC scRNA-seq (12k cells). The regulators are ranked by the TF enrichment score from LISA results in cluster-specific genes, and the color of the circles represents the averaged expression level of corresponding regulators in CD14 monocyte cells. The names of the top 10 TFs are labeled on the graph. **f** The rank of driver transcription regulators in the CD14 monocyte cells of PBMC scATAC-seq (10k cells). The regulators are ranked by the TF enrichment score from GIGGLE results in cluster-specific peaks, and the color of the circles represents the averaged regulatory potential of corresponding regulators in CD14 monocyte cells. The names of the top 10 TFs are labeled on the graph

One powerful application of single-cell technology is to de novo discover and annotate cell types, which relies on the accurate clustering of the cells [37]. MAESTRO integrates Seurat [28] to perform clustering and Presto [38] to perform differential analysis for scRNA-seq, which was reported to have superior clustering accuracy and running speed [23] (Fig. 3a). Clustering for scATAC-seq is more challenging, because the large number of peaks (*cis*-elements) and the predominantly binary read count at each peak in each cell from diploid genomes result in bigger yet sparser data matrices. We performed a systematic benchmark analysis using both simulated and published datasets on published scATAC-seq clustering methods, including scABC [23] and latent semantic indexing [39] followed by graph-based clustering (termed LSI here), cisTopic [24] followed by density-based clustering (termed cisTopic here), and snapATAC [17]. Our comparisons, together with a recent study assessing the performance of 10 scATAC-seq methods [40], suggested that LSI-based method is robust to sequencing depth and has the overall higher clustering accuracy (Additional file 4: Section A). Besides, LSI shows the best computing efficiency among all the tools tested (Additional file 2: Table S2). Therefore, we implemented LSI as the default scATAC-seq clustering method in MAESTRO but also incorporated cisTopic as an alternative option (Fig. 3b).

Modeling gene activities from scATAC-seq is the key step for understanding the cell-type identity of each cluster. MAESTRO uses the single-cell regulatory potential to model gene activities [41]. The presence of scATAC-seq peaks surrounding each gene reflects the potential transcriptional regulator (TR) bindings and their impact on the gene expression. Our regulatory potential (RP) model assumes that the effect of a scATAC-seq peak on the expression of a given gene is independent and additive, which follows an exponential decay with the distance from the peak to the transcription start site (TSS) (Additional file 3: Fig. S2, see the “Methods” section). Regulatory potential is calculated independently for each gene *i* in each cell *j* to reflect the accumulated regulation of the surrounding scATAC-seq peaks on the gene *i* and predict gene *i* expression in cell *j*. Besides increased promoter and enhancer accessibilities, active genes also have increased accessibility at the exon regions, which might reflect the binding of the RNA Pol II complex on chromatin during active transcription. In addition, we noticed that RP calculations could deviate from gene activity by the promoter and exon accessibilities of highly expressed genes nearby. Based on these observations, we implemented RP models with different combinations and tested their performances in integrating scATAC-seq with scRNA-seq (Additional file 4: Section B). We set the “enhanced RP model” with the best association with the gene expression from scRNA-seq as the default to calculate the gene activity score in MAESTRO, but allow users to pick other models. This RP model still weighs peaks by exponential decay from TSS, but sums all peaks on the given gene exons as if they are on TSS, normalizes the sum by total exon length, and excludes the peaks from promoters and exons of nearby genes (Additional file 3: Fig. S2).

### Automatic cell-type annotation

Annotating cell clusters from scRNA-seq is usually through marker genes from pre-existing knowledge. However, manual examination of the marker gene expression in each cluster is time-consuming and can create incompatible annotations between different datasets. MAESTRO could automatically annotate the cell type of clusters based on input signature files with lists of marker genes for each cell type. By default, MAESTRO includes signature files for immune cell types from CIBERSORT [42] and brain cell types [43] and optionally allows users to add custom cell-type signatures (Additional file 5: Table S3). For each cell cluster, MAESTRO calculates the average expression log fold change of marker genes in this cluster as compared to all the other cells, and the cell type with the highest expression log fold changes of marker genes is assigned to the cluster (Additional file 3: Fig. S3a). Clusters with average log fold change below zero for all the cell-type signatures are left unannotated, as they might represent rare populations of previously unknown cell types (see the “Methods” section). To annotate the cell identity for scATAC-seq clusters, MAESTRO first uses regulatory potential to infer gene expression at the single-cell level. Then, the genes with differential regulatory potentials in a specific cluster compared to all other clusters can be used as markers to annotate the cluster cell type as if in scRNA-seq.

We conducted automatic cell-type annotation on clusters from human PBMC scRNA-seq and scATAC-seq dataset using MAESTRO (Fig. 3a, b) and were able to annotate the cell types for both technologies (Fig. 3c, d). Clusters from scRNA-seq display distinct expression of marker genes, enabling the annotation at both lineage and sub-lineage levels (Additional file 3: Fig. S3b). In contrast, clusters from scATAC-seq could only be roughly annotated at the lineage level, suggesting that epigenetic profiles might reflect more lineage plasticity than transcriptome profiles (Additional file 3: Fig. S3c). We further compared the performance of MAESTRO cell-type annotation with several existing software [44, 45]. Using the LM22 immune signature [42], MAESTRO could successfully annotate the majority of the cell types in a sorted PBMC scRNA-seq dataset [16] (Additional file 3: Fig. S3d) and with the highest accuracy by median F1-score (Additional file 3: Fig. S3e). In addition, compared with other annotation tools that need cross-validation to train a classifier, MAESTRO shows good computational efficiency (Additional file 2: Table S2). Taken together, these results suggest that MAESTRO could annotate cell types from both scRNA-seq and scATAC-seq accurately and efficiently.

### Inference of transcriptional regulators

In single-cell RNA-seq analyses, identifying the transcriptional regulators which drive differential expression is crucial to understanding the underlying gene regulatory networks [46]. Our lab developed CistromeDB [47], which collected and processed ~ 24,000 ChIP-seq profiles for ~ 1300 human and mouse transcriptional regulators. With this comprehensive dataset, MAESTRO incorporates LISA [41] to predict the transcriptional regulators that shape the expression patterns in different scRNA-seq clusters. LISA builds an epigenetic model based on a list of cluster-specific genes and finds factors whose binding sites are most likely to regulate these genes. Since transcriptional regulators from the same family often have similar binding motifs and sometimes similar binding profiles, we grouped the transcription regulators with similar motifs (Pearson’s correlation coefficient of motif profiles > 0.7) [48]. After LISA identifies the candidate transcription regulators, MAESTRO reports all the regulators in the same motif group that are expressed (Additional file 3: Fig. S4a, b, Additional file 6: Table S4, see the “Methods” section). For example, LISA identifies STAT5B as the regulator in CD8 T cells; however, since the expression of STAT5B is not detected in CD8 T cells, MAESTRO reports STAT3, STAT4, and STAT1 instead, since they are expressed in CD8 T cells within the same regulator family (Additional file 3: Fig. S4c, d).

To predict the driver regulators from scATAC-seq clusters, MAESTRO employs the GIGGLE [49] method to identify the transcription regulators whose publicly available ChIP-seq profiles are highly enriched in the cluster-specific ATAC-seq peaks. Then, MAESTRO reports all the regulators that are predicted to be expressed, based on the regulatory potential, in the same motif family. For example, MAESTRO identified SPI1 (PU.1), CEBPA, CEBPB, and FLI1 as the top enriched regulators in the PBMC CD14 monocyte scATAC-seq cluster, which have been reported to be the lineage determinant factors for monocytes [50]. Reassuringly, the regulators identified from scRNA-seq and scATAC-seq of the same cell type are highly concordant, which increases the confidence of the MAESTRO-predicted driver regulators (Fig. 3e, f).

### Integrative analysis of scRNA-seq and scATAC-seq

**Fig. 4:**
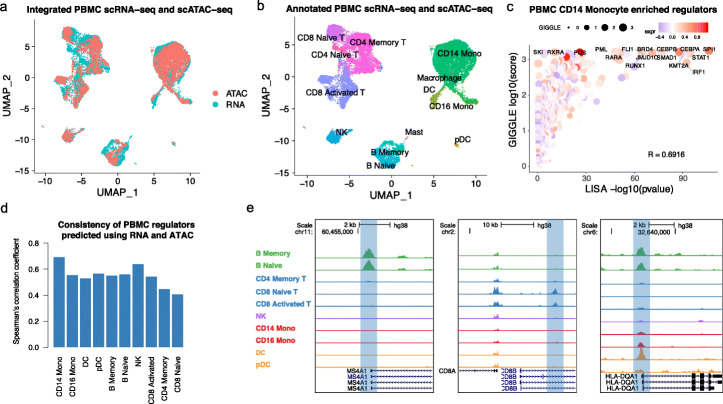
Integrated analysis of PBMC scRNA-seq and scATAC-seq data using MAESTRO. **a** UMAP visualization for joint clustering of human PBMC scRNA-seq (12k cells) and PBMC scATAC-seq (10k cells). Colors represent the cells from different technologies. The cells are joined by CCA on gene expression level and regulatory potential from MAESTRO. **b** UMAP visualization for joint clustering of human PBMC scRNA-seq and scATAC-seq. The cells are joined by CCA on the gene expression level and regulatory potential from MAESTRO. Colors represent the cell types, for which are generated using the scRNA-seq dataset and transferred to the scATAC-seq dataset. **c** The rank of driver regulators in CD14 monocyte cells of the PBMC dataset. The *x*-axis represents the TF enrichment score from LISA results in cluster-specific genes using scRNA-seq; the *y*-axis represents the TF enrichment score from GIGGLE results in cluster-specific peaks using scATAC-seq. The color of the circles represents the averaged expression level of corresponding regulators in CD14 monocyte scRNA-seq cells, and the size represents the TF enrichment score using GIGGLE in CD14 monocyte scATAC-seq cells. The names of the top 10 TFs from LISA and GIGGLE are labeled on the graph. **d** Comparison of transcriptional regulators predicted using scRNA-seq and scATAC-seq in each cell type for PBMC dataset. The *y*-axis represents the Spearman’s correlation coefficient between LISA-predicted TF enrichment score and GIGGLE-predicted TF enrichment score for all the tested regulators. **e** Genome browser view of MS4A1 (B cells), CD8A (T cells), and HLA-DQA1 (monocytes and DCs) locus. The *pseudo*-bulk ATAC-seq profiles are generated by pooling together cells within each cell type. The *y*-axis represents the sequence depth-normalized ATAC-seq signals (reads per million mapped reads (RPM))

Previous studies suggested that scRNA-seq has better power in defining cell types with distinct marker genes, while scATAC-seq is superior for the identification of lineage determinant regulators [10, 39]. Integrative analysis of scRNA-seq and scATAC-seq combines the advantages of both data types to provide a deeper understanding of the gene regulation in the experimental system. To integrate the cells from scRNA-seq and scATAC-seq, MAESTRO first calculates the regulatory potential for each gene in each cell, which measures the scATAC-seq reads near the gene weighted by an exponential decay of the read distance to the gene TSS. Then, MAESTRO performs a canonical correlation analysis (CCA) [28] between gene expression from scRNA-seq and regulatory potential from scATAC-seq. CCA captures the common variance between the two datasets and projects them into the same low-dimensional space, which essentially treats the two data platforms as two batches of data from the same platform (Fig. 4a). A pair of cells, one from scRNA-seq and the other from scATAC-seq, can be anchored using mutual nearest neighbors after dimension reduction [51]. Then, MAESTRO transfers the cell-type labels from scRNA-seq to scATAC-seq using the anchored cell pairs (Fig. 4b). This approach can roughly preserve the original clustering structures after integration, which allows cell-type labels to be matched between scRNA-seq and scATAC-seq clusters.

The integration between scRNA-seq and scATAC-seq can improve regulator inference. After integrating scRNA-seq and scATAC-seq cells, MAESTRO combines the transcriptional regulators predicted from scRNA-seq (LISA) and scATAC-seq (GIGGLE) cluster and uses the rank product to combine the two. The final candidate regulators are further filtered based on the regulator expression from scRNA-seq (Fig. 4c, see the “Methods” section). While the regulators in PBMC clusters that are predicted from scRNA-seq and scATAC-seq are mostly concordant (Fig. 4d), there are also interesting differences between the two approaches. For example, RXRA was only predicted to be a regulator from scATAC-seq in CD14 monocytes (Fig. 4c), and HDAC3 was only predicted to be a regulator from scATAC-seq in CD8 naive T cells (Additional file 3: Fig. S5a). RXRA is known to control the innate inflammatory response through the upregulation of chemokine expression in monocytes [52], while HDAC3 has been reported to restrain the CD8 lineage gene expression and maintain a bi-potential state for CD4+CD8+ cells [53]. Those examples demonstrate that the transcription regulators predicted from scATAC-seq are sufficiently meaningful and can complement the predictions from scRNA-seq.

Integration can also make the prediction of the *cis*-elements in rare cell types possible. After transferring labels from scRNA-seq, we re-clustered the cells in the scATAC-seq data and formed pseudo-bulk samples for each cell type in the human PBMC dataset, with sub-lineage clusters and rare cell types recovered from scRNA-seq (Fig. 4e). To identify potentially rare *cis*-elements that were missed from the aggregated peak calls in the earlier step of the scATAC-seq analysis, we called peaks on each cell cluster separately (Additional file 3: Fig. S5b). Although most of the peaks are already presented in the aggregated peak calls, in some rare populations such as plasmacytoid dendritic cells (pDCs), nearly 14% of the peaks are missing in the single-cell aggregated peak calls (Additional file 3: Fig. S5c). Many of these cluster-specific new peaks, e.g., a *cis*-element in the intronic region of TSPAN13 which is specifically accessible in plasmacytoid dendritic cells (pDCs) (Additional file 3: Fig. S5d,e), might be functional and regulate nearby genes in a cluster-specific manner.

### Analysis of bone marrow microenvironment in healthy donors and CLL patients

**Fig. 5:**
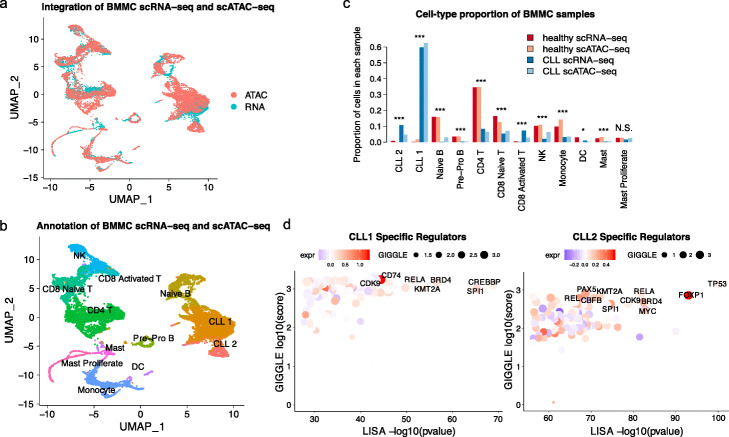
The dramatic change of bone marrow microenvironment in a CLL patient versus a healthy donor. **a** UMAP visualization for joint clustering of human BMMC scRNA-seq (5k cells) and scATAC-seq (9k cells) from the CLL patient and the healthy donor. Colors represent the cells from different technologies. The cells are joined by CCA on gene expression level and regulatory potential from MAESTRO. **b** UMAP visualization for joint clustering of human BMMC scRNA-seq (5k cells) and scATAC-seq (9k cells) from the CLL patient and the healthy donor. The cells are joined by CCA on gene expression level and regulatory potential from MAESTRO. Colors represent the cell types, which are generated using the scRNA-seq dataset and transferred to the scATAC-seq dataset. **c** Cell-type proportions of the CLL patient and the healthy donor. The total number of cells in each sample (CLL patients or healthy donors) should add up to 1. The scRNA-seq and scATAC-seq are quantified separately. Statistic significance is evaluated using two proportion *z* test, ***p < 2.2e−16, *p ≤ 0.05, ^N.S.^*p* > 0.05. **d** The rank of driver regulators in CLL1 (left) and CLL2 (right) cluster of the BMMC dataset. The *x*-axis represents the TF enrichment score from LISA result on differentially expressed genes between CLL1 and CLL2 clusters in scRNA-seq; the *y*-axis represents the TF enrichment score from GIGGLE result on differential peaks between CLL1 and CLL2 clusters in scATAC-seq. The color of the circles represents the averaged expression level of regulators in corresponding clusters of scRNA-seq, and the size represents the TF enrichment score using GIGGLE in corresponding clusters of scATAC-seq. The names of the top 10 TFs from LISA and GIGGLE are labeled on the graph

To demonstrate the performance of MAESTRO on complex sample types, we applied it to the scRNA-seq (5k cells) and scATAC-seq (9k cells) dataset of human bone marrow-derived mononuclear cells (BMMCs) from a healthy donor and a CLL patient. We first performed the alignment, quality control, clustering, and annotation on scRNA-seq and scATAC-seq dataset separately, then integrated the two (Fig. 5a, b). We combined the LM22 signatures as well as signatures from early-stage B cell development [42, 54] to annotate the clusters, and identified four different B cell populations from scRNA-seq (pre-pro B, naive B, CLL 1 and CLL 2) (Fig. 5b and Additional file 3: Fig. S6a). The cell-type labels were transferred to scATAC-seq clusters after integration (Fig. 5b and Additional file 3: Fig. S6b).

We next investigated cell population changes in the CLL patient compared to the healthy donor. As expected, the two CLL clusters (CLL1 and CLL2) are mainly in the CLL patient, while the pre-pro B cell and naive B cell population are almost exclusively in the healthy donor (Fig. 5c). This observation suggests the dominance of malignant cells in the bone marrow of the CLL patient. Interestingly, immune cell diversity is much higher in the healthy donor compared to the CLL patient, with higher fractions of CD4 T, CD8 naive T, NK, monocyte, and mast cell populations. The only expanded population in CLL patients is CD8 activated T cells, supporting the major role of cytotoxic CD8 T cells in anti-tumor activity in CLL patients (Fig. 5c). Cell-type composition estimates are consistent between scRNA-seq and scATAC-seq, demonstrating MAESTRO’s ability to robustly integrate scRNA-seq and scATAC-seq data from tissue samples with complex composition.

Our analysis suggests that the majority of B cells from the CLL patient are clustered into two distinct populations. The CLL1 cluster still preserves the ability to express IGHM, a gene highly expressed in pre-pro B cell and naive B cells (Additional file 3: Fig. S6c). The CLL2 cluster highly expresses RGS1 (Additional file 3: Fig. S6d), which was reported to be associated with poor prognosis in B cell malignancies [55]. Hypothesizing that distinct regulators might drive gene expression in these two CLL populations, we applied MAESTRO to identify the cluster-specific regulators using the differentially expressed genes from scRNA-seq and differentially accessible peaks from scATAC-seq data between CLL1 and CLL2 clusters. Consistent with the gene expression pattern, the top predicted regulators in the CLL1 cluster are similar to the regulators in naive B cells, such as SPI1 and CREBBP (Fig. 5d and Additional file 3: Fig. S6e, f). In contrast, the top MAESTRO-predicted regulators in the CLL2 cluster include TP53 and FOXP1, indicating that CLL2 might represent a distinct malignant cell population. TP53 is a well-known tumor suppressor and is frequently mutated or deleted in CLL patients [56], while FOXP1 was reported to have an oncogenic role in B cell lymphoma and associated with poor clinical outcome [57, 58]. In summary, these results demonstrated MAESTRO’s utility in identifying transcriptional regulators from both scRNA-seq and scATAC-seq datasets in complex samples.

### MAESTRO outperforms other methods in integrating scATAC-seq with scRNA-seq

**Fig. 6:**
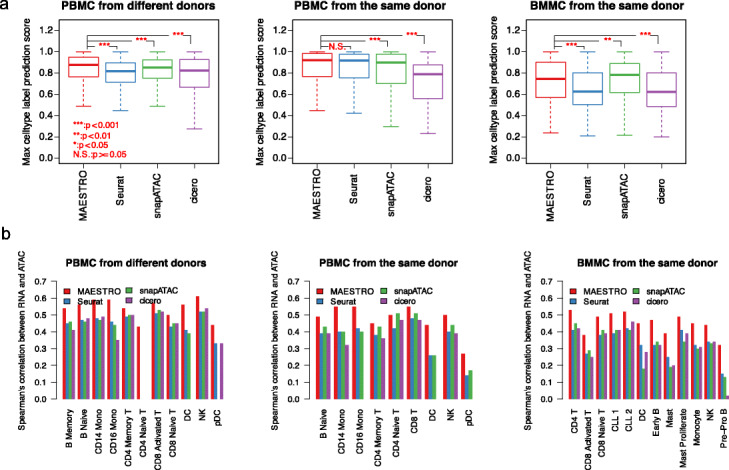
MAESTRO outperforms the existing software in integrating scRNA-seq and scATAC-seq dataset. **a** Comparison of the cell-type label prediction score distribution after integration of the scRNA-seq and scATAC-seq using gene activity scores from MAESTRO, Seurat, snapATAC, and cicero. The comparisons were made on three independent datasets: PBMC from different donors, PBMC from the same donor, and BMMC from the same donor. Statistical significance was evaluated using Wilcoxon rank-sum test. **b** Comparison of the consistency between gene expression and gene activity scores from MAESTRO, Seurat, snapATAC, and cicero. The comparisons were made on three independent datasets. The *x*-axis represents the different cell types, and the *y*-axis represents the Spearman’s correlation coefficient between the expression level from scRNA-seq and gene activity score from scATAC-seq within each cluster. Only the top 2000 highly variable genes were used in the analysis

Finally, we sought to benchmark different computational methods, including SnapATAC, cicero, Seurat, and MAESTRO, on the performance of integrating scATAC-seq regulatory activities with scRNA-seq data. We evaluated the performance using three independent datasets: dataset #1, from the human PBMC from different donors (12k cells scRNA-seq and 10k cells scATAC-seq); dataset #2, from the human PBMC from the same donor (2k cells scRNA-seq and 10k cells scATAC-seq); and dataset #3, from the human BMMC from the same donor (5k cells scRNA-seq and 9k cells scATAC-seq, Fig. 6 and Additional file 3: Fig. S7). After label transfer using CCA, we generated the label prediction score distribution for all three datasets. Compared to the other three methods, the integration using MAESTRO regulatory potential model has overall higher prediction scores and a larger number of cells with high-quality predictions, defined as prediction score > 0.5 (Fig. 6a, Additional file 3: Fig. S8a, Additional file 7: Table S5). For dataset #1, cicero failed to align memory B cells, CD 16 monocytes, and DC cells, and snapATAC failed to align pDC cells (Additional file 3: Fig. S7 and Additional file 7: Table S5). All of the tested methods failed to align CD4T naive, macrophage, and mast cells. The failure in integration might be due to the under-representation of macrophage and mast in the scATAC-seq dataset, as the scRNA-seq samples and scATAC-seq samples were collected from different donors. We indeed did not observe the macrophage and mast marker gene activity from the scATAC-seq dataset (Additional file 3: Fig. S8b, c). In addition to these cell types in PBMC dataset from the different donors, MAESTRO has a 100% transfer rate for three datasets at the cluster level (Additional file 7: Table S5).

We further evaluated the consistency between gene regulatory activities from scATAC-seq after label transfer and gene expression levels from scRNA-seq in the same cell type. Again, MAESTRO showed the best consistency in almost all the cell types, whether the correlation was calculated using all the genes or only the top 2000 variable genes (Fig. 6b and Additional file 3: Fig. S8 d,e). Collectively, these analyses suggest that MAESTRO has the best performance not only in aligning the cells between scATAC-seq and scRNA-seq, but also in predicting the gene expression level from scATAC-seq.

To test the scalability of MAESTRO in handling large and complex single-cell dataset, we performed the integration analysis on a public basal cell carcinoma (BCC) scRNA-seq (53k cells) and scATAC-seq (38k cells and 0.5M peaks). MAESTRO could successfully cluster, annotate, and integrate scRNA-seq and scATAC-seq dataset (Additional file 3: Fig. S9). This demonstrates MAESTRO’s scalability and potential to be used in consortium projects for scRNA-seq and scATAC-seq such as *Tabula Muris* [59] and Human Cell Atlas [60].

## Discussion and conclusions

The recent development of single-cell technologies has brought paradigm shifts to investigating cellular diversity from a multi-omic perspective. While these technologies have wide applications in understanding complex biological systems such as tumor, brain, and immune and developmental systems, they also create numerous computational challenges. MAESTRO is a comprehensive analysis workflow that provides full analysis solutions for integrating scRNA-seq and scATAC-seq on multiple single-cell platforms. Compared with existing tools, the regulatory potential model adopted by MAESTRO is superior in integrating scATAC-seq data with scRNA-seq. In addition, the automatic cell-type annotation from MAESTRO is very useful, especially since the increasing number of single-cell datasets makes manual annotation more impractical. Although several methods have been developed for identifying regulators from scRNA-seq or scATAC-seq, most of them rely heavily on motif information and ignore cell type-specific TF binding [17, 24, 25]. Using the comprehensive collection of ChIP-seq profiles on more than 1300 transcriptional regulators from CistromeDB, MAESTRO can robustly identify relevant regulators from both scRNA-seq and scATAC-seq datasets, and allow users to visualize the integrated predictions. We implemented MAESTRO using the Snakemake workflow [35] and deposited the package under the Conda environment, which allowed MAESTRO to be installed and executed with simple commands. These features make MAESTRO an effective workflow for comprehensive and integrative analysis of scRNA-seq and scATAC-seq data.

MAESTRO models gene expression activity from scATAC-seq using a combination of two models: one related to the effects of *cis*-regulatory elements, the other related to the effects of transcription. The first is a regulatory potential model that has shown great efficacy in modeling the effect of TFs on regulating gene expression [41, 61]. The second considers the peaks on exon regions, which includes possible alternative promoters, and removes interfering signals from adjacent promoters and exons. The combined model showed the best performance in gene expression prediction using scATAC-seq data. Recently, another model proposed in ArchR (Granja et al.) also uses the regulatory potential model [41, 61] in combination with a gene body component to model the gene activity from scATAC-seq [62]. ArchR uses a gene boundary model to exclude interference from other genes, although in cases such as near-neighbor divergently transcribed gene pairs, this is likely to eliminate many real long-range *cis*-regulatory effects [63, 64]. In terms of gene body accessibility, ArchR considers the whole gene body while MAESTRO uses only the exon regions. Despite these differences, both MAESTRO and ArchR can model gene expression activity from scATAC-seq well, with comparable performance in integrating scATAC-seq with scRNA-seq.

Despite the aforementioned merits, MAESTRO still has some limitations which deserve future development. For example, in transcriptional regulator inference, information on the TF motifs bound by collaborating TFs might provide additional insights to distinguish the different family members to complement the clues from gene expression. Future machine learning approaches to infer TF binding sites from chromatin accessibility profiles could help improve this function. Currently, MAESTRO is able to handle large single-cell datasets of one million cells by processing all the gene expression and peak quantification matrices in the compressed sparse matrix format and stored using HDF5. However, MAESTRO analysis functions were built in R, so they have limited memory and processing efficiency. Future migration of the framework functions from R to Python and Cython, and deployment of the workflow on the Cloud will improve MAESTRO’s capability in handling even bigger single-cell datasets.

The current integrated functions of MAESTRO, which support the analysis of scRNA-seq and scATAC-seq, can be adapted to other single-cell gene regulation techniques such as single-cell ChIP-seq [65], cut & tag [66], or spatial transcriptomics [67]. As single-cell datasets accumulate over time, MAESTRO not only provides a scalable and uniform workflow to process these data, but also benefits from these data in enhancing MAESTRO functions. For example, with more cell-type gene signatures generated from scRNA-seq datasets, the automatic cell-type annotation in MAESTRO will be more precise and could be further improved using supervised classification methods such as scmap [45, 68] and cellAssign [69]. If more scATAC-seq datasets become available, MAESTRO could generate a comprehensive atlas of cell type-specific regulatory landscapes. This atlas can in turn facilitate cell clustering, annotation, and regulatory modeling. We foresee MAESTRO becoming an important tool to help biologists with scRNA-seq and scATAC-seq data to derive a deeper understanding of cellular heterogeneity and regulatory dynamics.

## Methods

### MAESTRO pipeline

#### Data formatting and barcode demultiplexing

MAESTRO supports multiple scRNA-seq and scATAC-seq platforms. ScRNA-seq from Smartseq2 protocols and scATAC-seq from microfluidics protocols do not need barcode demultiplexing. ScRNA-seq with variable barcodes like Drop-seq, inDrop, and 10X Genomics are demultiplexed using STARsolo according to the given barcode whitelist. ScATAC-seq data from sci-ATAC-seq, dsci-ATAC-seq, or 10X Genomics are demultiplexed using custom python codes.

#### Alignment, sorting, and duplicates removal

After demultiplexing, reads are aligned to the hg38 or mm10 genome. MAESTRO uses STAR solo mode [70] to align the scRNA-seq reads and minimap2 short-read mapping mode to align the scATAC-seq reads. All alignments are then sorted by genome coordination using samtools [71]. PCR duplicates from the same barcodes are removed using the MarkDuplicates function from Picard tools.

#### ScRNA-seq quality control and barcode selection

MAESTRO performs quality control for scRNA-seq in two aspects: the bulk and the single-cell levels. At the bulk level, MAESTRO summarizes the mapping statistics, reads quality, GC content, nucleotide composition bias, reads distribution, and gene body coverage using RseQC [72]. The percentage of mapped reads indicates sample quality, as sample contamination or improper processing could decrease mappability. The CDS read distribution checks proper RNA processing and library preparation, which ensures accurate expression quantification. And the gene body coverage evaluates sample read bias over 3′ or 5′ of the transcripts. At the single-cell level, MAESTRO applies the following criteria to select high-quality barcodes: (1) the barcode should be found in more than 1000 unique reads or UMIs, (2) The barcode should be found in at least 500 genes, (3) no more than 5% of the reads containing this barcode could be aligned to the mitochondria genome, and (4) if spike-in is available, no more than 5% of the reads containing this barcode are spike-in reads. Only barcodes passing the single-cell QC are labeled as high-quality cells and used for downstream expression quantification and clustering analysis. All the QC parameters are configurable and can be tuned accordingly.

#### ScATAC-seq quality control and barcode selection

MAESTRO performs quality control for scATAC-seq in two aspects as for scRNA-seq. At the bulk level, MAESTRO summarizes the mapping statistics and the fraction of mitochondria reads and reads in promoter regions and peak regions, and generates the fragment size distribution using custom codes. The percentage of duplicated reads shows whether the library is over-amplified due to limited starting material. The percentage of reads in mitochondria genes and peak regions is a widely used metric to evaluate the signal-to-noise ratio of ATAC-seq data quality [1]. The last feature for bulk-level QC is fragment periodicity, which examines the insert size distribution of the sequenced fragments. It should show a periodicity of approximately 200 bp due to nucleosome protection of the chromatin to transposase cutting [3]. At the single-cell level, MAESTRO applies the following criteria to select high-quality barcodes. (1) The barcode should be found in more than 1000 unique reads. (2) At least 10% of the reads containing the barcode should present in gene promoter regions. We observed that the peak calls from the single-cell aggregated data sometimes are dominated by the major populations, and barcodes filtering using the fraction of reads in peak regions (FRiP score) might eliminate the rare populations from the sample. Therefore, we used the fraction of reads in promoter regions instead of the FRiP score to filter the barcodes. (3) No more than 10% of the reads containing this barcode could be aligned to the mitochondria genome. Only barcodes passing the single-cell QC are labeled as high-quality cells and used in the downstream analysis. All the QC parameters are configurable and can be tuned accordingly.

#### ScRNA-seq expression quantification

MAESTRO applies different algorithms to calculate the reads/UMI count from different scRNA-seq platforms. Data from Smartseq2 protocol are processed using RSEM to generate the read count for each transcript [73]; data from Drop-seq or 10X Genomics platform are quantified using STARsolo. By default, MAESTRO stores all the data in memory in a sparse matrix and save the data to disk with HDF5 format, in order to achieve memory efficient and improve the scalability of MAESTRO.

#### ScATAC-seq peak calling and binarization

For scATAC-seq data using microfluidics protocols, only cells passing QC are merged and called for peaks. For scATAC-seq data using sci-ATAC-seq, 10X Genomics, or other barcoded protocols, aligned bam files are directly used for peak calling. MAESTRO performs peak calling using MACS2 on the single-cell aggregated data [74], with the options set as “-B -q 0.05 –nomodel –extsize=50 --SPMR.” Peaks overlapping with ENCODE blacklist [75] are removed, and only peaks from autosome and chromosomes X and Y are used in the downstream analysis. MAESTRO also provides the option to add custom-defined *cis*-elements for downstream analysis. In the scATAC-seq dataset, the accessible elements in every single cell should be either on or off (1/0) due to the nature of the diploid genome. After peak calling, MAESTRO calculates the peak count for each barcode that passes QC and then converts the peak count matrix to binary matrix using custom python codes.

#### Normalization

MAESTRO adopts Seurat for scRNA-seq normalization. By default, MAESTRO employs the global scaling normalization method in Seurat to scale the expression in each cell to 10,000, then log-transformed the result. For scATAC-seq data, no additional normalization methods are used if LSI or cisTopic is used for the clustering analysis [24, 39]. If scABC is used for clustering analysis, the peak matrix will be weighted by the total number of peaks present in each cell [23].

#### Feature selection

For scRNA-seq data, MAESTRO employs the FindVariableFeatures function in Seurat to identify genes that exhibit high cell-to-cell variation [28]. By default, MAESTRO uses the variance-stabilizing transformation (vst) to adjust the variance and returns the top 2000 genes with the highest standardized variance. No feature selection is performed for the scATAC-seq dataset, and all input peaks were used in the downstream dimension reduction analysis.

#### Dimension reduction and determine significant components

For the scRNA-seq dataset, before dimension reduction, MAESTRO scales the expression matrix to make sure that the mean expression of each gene across cells is 0, and the variance across cells is 1. The users can also remove unwanted variations like mitochondrial contamination, different stages of cell cycle at this step, by providing unwanted variations as features to regress out. After scaling, MAESTRO performs principal component analysis (PCA) on top variable features to reduce the dimension of the dataset. An elbow plot is used to visualize the variance of each PC and identify the “elbow” point to determine the significant PCs. If not set, the top 15 PCs are selected by default for downstream analysis. For the scATAC-seq dataset, both PCA and LSI are provided for dimension reduction. For LSI analysis, MAESTRO first computes the term frequency-inverse document frequency (TF-IDF) on the scATAC-seq peak count matrix, followed by singular value decomposition (SVD) to reduce the dimensionality to 50. Among our benchmark, TF-IDF transformation followed by SVD, Louvain algorithm for distance calculation, and *K*-nearest neighbor (KNN) analysis for cluster identification had the best performance in terms of clustering accuracy and structure; we thus set LSI as the default dimension reduction method for scATAC-seq data analysis. MAESTRO also adopts cisTopic to conduct topic modeling and reduce the dimension of the dataset; if cisTopic is selected, by default, 30 topics are used in the cisTopic analysis.

#### Clustering

MAESTRO employs the graph-based clustering method in Seurat for scRNA-seq clustering analysis. Briefly, MAESTRO first builds a *K*-nearest neighbor (KNN) graph using the reduced dimensions from the previous step and then refines the edge weights between two cells based on the Jaccard similarity of their neighborhoods, this function is adopted from the FindNeighbors function in Seurat. To cluster the cells, MAESTRO uses the FindClusters function, which applies the Louvain algorithm to cluster cells together iteratively. The default clustering resolution for scRNA-seq is set to 0.6, and users can also tune the parameters for different conditions. For scATAC-seq clustering, if LSI is used, MAESTRO will perform similar graph building and cluster identifications like scRNA-seq analysis, and the default clustering resolution is set to 0.6. If cisTopic is used, MAESTRO performs Uniform Manifold Approximation and Projection (UMAP) [76] analysis on the cell-topic distributions to further reduce the dataset to two dimensions and then applies a density-based clustering method DBSCAN [77] to identify potential clusters; the default reachability distance is set to 0.75, and reachability minimum number of points is set to 10. If scABC is used, by default, MAESTRO sets *K* to 10 for the *K*-medoid clustering analysis.

#### Visualization of single-cell clusters

The clustering results from both the scRNA-seq and scATAC-seq are visualized using UMAP. The UMAP function is already included in Seurat and cisTopic. To visualize the clustering result of scABC, MAESTRO adopts the uwot package and performs the UMAP analysis using the Spearman’s correlation between different cells as the distance [23].

#### Differential expression and peak calling

For the scRNA-seq analysis in MAESTRO, we optimized the FindAllMarkers function in Seurat to perform differential expression and identify the positive markers for each cluster. The default differential expression method is achieved using “presto,” a fast version of the Wilcox rank-sum test implemented in R, and other methods that had been already incorporated in Seurat like ROC, *t* test, MAST, and DESeq2 are also supported [22, 38, 78]. Genes with a log fold change greater than 0.25, minimum presence fraction in cells of 0.25, and *p* value less than 1E−5 are identified as marker genes for each cluster. For the scATAC-seq analysis, MAESTRO first normalizes the binary peak count matrix by the number of peaks presented in each cell, then performs the differential peak analysis using “presto” on the normalized peak count matrix. Peaks with logFC greater than 0.1, minimum presence fraction in cells of 0.01, and *p* value less than 1E−5 are identified as cluster-specific peaks for each cluster. All these threshold parameters are tunable in the MAESTRO package.

#### Regulatory potential score to quantify gene activity at the single-cell resolution for scATAC-seq

To model the gene activity from scATAC-seq, MAESTRO calculates the gene regulatory potential score for each gene in each cell using matrix multiplication based on the formula below.

$$ R={W}^TB $$

Matrix *B* is a binary matrix output from the single-cell ATAC-seq peak calling and binarization, and *B*_*ij*_ represents the occurrence peak_*i*_ in cell_*j*_. Matrix *W* represents the regulatory potential matrix, and *W*_*ij*_ represents the weight of regulatory potential of each peak_*i*_ to each gene_*j*_ calculated from an exponential function on the distance between the center of peak_*i*_ and the transcription start site of gene_*j*_ (*d*_*ij*_) with a half-decay of *d*_0_:

$$ {W}_{ij}={2}^{-\frac{d_{ij}}{d_0}} $$

The *d*_0_ can be customized by users, and our recommendation is that 1 kb shall be used for promoter-driven regulation and 10 kb shall be used for enhancer-driven regulation, and the default *d*_0_ for scATAC-seq is set to 10 kb. For a given gene_*j*_, if *d*_*ij*_ of peak_*i*_ is over 150 kb, the weight *W*_*ij*_ will be less than 0.0005 if *d*_0_ is 10 kb. To save the computation time, we set the *W*_*ij*_ as 0 if the peak to TSS distance is over 150 kb. In an “enhanced RP model,” we did further adjustments to the weights. If the peak_*i*_ is located at the exons region of the gene_*j*_, the weight *W*_*ij*_ is set to 1 first (as if *d*_*ij*_ = 0), then further normalized by total exon length of the gene_*j*_ (i.e., *W*_*ij*_ = 1/total_exon_length); and if the peak_*i*_ is located in the promoter or exon regions of any nearby genes, then *W*_*ij*_ is set to 0 (i.e., the peak is excluded). After the matrix multiplication, the matrix *R* stores the final scores of regulatory potential, in which *R*_*ij*_ represents gene_*i*_’s regulatory potential score in cell_*j*_.

#### Cell-type annotation based on differentially expressed or regulated genes

MAESTRO performs automatic cell-type annotation in a supervised manner, which requires the pre-existing knowledge of marker genes for each cell type. Given the gene signatures of each cell type, for each cluster, MAESTRO calculates the summed logFC (cells in one cluster versus all other cells, which could be both positive or negative) of marker genes divided by log2 total number of marker genes as the cell-type scores of the input gene signature; the cell type of gene signature with the highest score is annotated as the cell-type identity of that cluster. The minimum gene signature score is set to 0, and if the score of all input signatures is less than 0, the cluster will be annotated as “others.” By default, the immune LM22 gene signature from CIBERSORT [42] is used to annotate the cell types (Additional file 2: Table S2); we have also included signatures from adult mouse brain and *Tabula Muris* in the MAESTRO package [43], and other user-defined signatures are also supported. For the scATAC-seq dataset, MAESTRO performs the cell-type annotation using the gene regulatory potential to represent the gene expression, and the logFC of gene regulatory potential between one cluster and all the other cells is used to calculate the gene signature scores.

#### Cell-type annotation of scATAC-seq clusters based on bulk chromatin accessibility data

MAESTRO supports automatic cell-type annotation for scATAC-seq dataset using the publicly available bulk chromatin accessibility data. To generate a high-quality annotation index for scATAC-seq clusters, we first clustered the chromatin accessibility datasets (DNase-seq and ATAC-seq) in the Cistrome database into 80 clusters [47]. Each Cistrome cluster identity was determined by the majority of cell type or tissue type information of datasets within that cluster. Then, the identity was assigned to all the datasets within that cluster. MAESTRO utilizes GIGGLE to evaluate the enrichment of bulk chromatin accessibility peaks on cluster-specific peaks from scATAC-seq data. It then transfers the Cistrome cluster annotation from the most enriched bulk chromatin accessibility data to the scATAC-seq cluster as its cell-type annotation.

#### Prediction of driver regulators for scRNA-seq and scATAC-seq

Based on the marker genes or the cluster-specific peaks from each cluster, MAESTRO could predict the potential driver transcription regulators in each cell type. For the scRNA-seq dataset, MAESTRO incorporates LISA [41], which utilizes the transcriptional regulator binding profiles from CistromeDB [47] to identify the potential regulators shaping the expression pattern of each cluster. LISA first models the epigenetic landscape based on the input marker genes as well as public epigenomic profiles (DNase-seq, H3K27ac ChIP-seq) in CistromeDB, then performs in silico detection of TF binding sites on the epigenetic landscape to evaluate the essentiality of the transcriptional regulators. All the candidate regulators are ranked by their expression within that cluster to identify potential significant ones. For scATAC-seq, MAESTRO utilizes GIGGLE [49] to evaluate the enrichment of transcriptional regulator ChIP-seq peaks on cluster-specific peaks from scATAC-seq data. The GIGGLE score is a composite of -log10 *p* value and log2 odds ratio after querying regulator peaks in the cluster-specific peaks. For the ChIP-seq dataset of the same factor, only the dataset with the highest GIGGLE score is kept. All the candidate regulators are further ranked by its RP within that cluster to identify potential significant ones. Currently, 1314 regulators, including both transcription factors and chromatin regulators, are supported in MAESTRO regulator analysis.

#### Transcription factor clustering based on motif similarities

To improve the transcription factor prediction and correct the enrichment scores, MAESTRO clusters transcription factor based on motif similarities. We downloaded 769 and 529 positional weight matrices (PWM), which represent 680 human and 453 mouse transcription factors from the HOCOMOCO v11 database, respectively [48]. Within each species, the similarity between TF motif models is calculated using the CCAT PWMclus tool [79], which uses the Pearson correlation coefficient (PCC) weighted by information content as the similarities. We clustered the PWMs by hierarchical clustering and identified the motif clusters with the threshold of similarity greater than 0.7. For humans, 257 PWM clusters were identified and 109 of them contains two or more PWMs (Additional file 5: Table S3). The highest enrichment score of TFs within the same motif clusters is assigned to all the TFs belong to that cluster, and the TFs are further ranked by the averaged expression level for scRNA-seq and regulatory potential level for scATAC-seq in that cluster. TFs with mean expression level equal to 0 or RP level less than 0.5 are filtered from the output. The driver TF candidates of the cluster should have both significant enrichment scores and expression levels.

#### Visualization of marker genes or enriched regulators

MAESTRO provides two functions, “VisualizeVlnplot” and “VisualizeUMAP,” for the visualization of the expression level or regulatory potential of marker genes or regulators in scRNA-seq and scATAC-seq datasets. Besides, MAESTRO provides a genome browser function “ATACViewTracks” for visualizing the scATAC-seq signals across the chromosome in different clusters. For better visualizing the predicted regulators, MAESTRO provides the function named VisualizeTFenrichment to generate the TF rank plot (as Fig. 3e, f and Fig. 4c) and only output the top 100 regulators in each cluster. The size of the point reflects the enrichment score of TFs from LISA for scRNA-seq and GIGGLE for scATAC-seq, and the color indicates the expression level from scRNA-seq or regulatory potential scores from scATAC-seq. If regulators are predicted from both scRNA-seq and scATAC-seq, MAESTRO will combine the ranks using the rank product and use the combined ranks to determine the top regulators. The candidates with gene expression levels equal to 0 are further removed from the output.

#### Integrative analysis of scRNA-seq and scATAC-seq clusters

MAESTRO integrates the scRNA-seq and scATAC-seq clusters with canonical correlation analysis (CCA) and provides joint visualization of the cells together. The scRNA-seq clusters with cell-type annotations are generated in previous steps. The scATAC-seq clusters are generated based on the peak count matrix as described before. MAESTRO then attaches the gene regulatory potential matrix to the scATAC-seq clusters, scales and log-transforms the RP matrix, identifies variable features, and then combines the scRNA-seq clusters with scATAC-seq clusters using the FindTransferAnchors function incorporated in Seurat. After identifying the transfer anchors, the cell-type annotation from scRNA-seq clusters can be transferred to scATAC-seq clusters using the TransferData function. To visualize all the cells in the same low dimensional space, MAESTRO uses the same anchors during the label transferring analysis and imputed the scRNA-seq gene expression using scATAC-seq regulatory potential score on highly variable genes from scRNA-seq, for which default is set to the top 2000 variable genes. The measured scRNA-seq and imputed scRNA-seq data are then merged together and scaled to normalize the variance and mean. Finally, MAESTRO performs PCA for dimension reduction and uses UMAP to visualize all the cells together.

#### Summary result and HTML output

For users to better understand the results from the MAESTRO workflow, we provide output files in HTML format to summarize the mapping statistics, quality control analysis from RseQC, single-cell QC plot, clustering result, cell-type annotation result, and transcription regulator predictions from LISA or GIGGLE. The HTML output contains three different sections, the scRNA-seq page, the scATAC-seq page, and the integration page (Additional files 8, 9, and 10). The description of each result and the normal range of QC metrics are also included in the HTML output.

### Clustering evaluation based on the simulated scATAC-seq dataset

To benchmark the clustering performance of different methods on scATAC-seq datasets, we generated the simulated scATAC-seq dataset from bulk ATAC-seq experiments. The BAM files of ATAC-seq from 10 different cell types were downloaded from CistromeDB, using the accession numbers GSM1817207, GSM1876022, GSM2083780, GSM2243034, GSM2243040, GSM2386582, GSM2439074, GSM2476338, GSM2692563, and GSM2898847. We randomly sampled the reads from each BAM file and simulated 200 single-cell ATAC-seq datasets at 4 different sequence depths, which are 1000, 2500, 5000, and 10,000 total reads. For each sequencing depth, 2000 cells were generated in total for the 10 cell types. Then, we merged the simulated bam files and called peaks, generated the peak by cell binary count matrix, and performed clustering analysis using scABC, cisTopic, and LSI. For scABC, we set *k* = 10 for the *k*-means-based clustering. For cisTopic, the topic number used was 30. For LSI, the top 50 dimensions are used after TF-IDF conversion, and the clustering resolution was set to 0.6. For snapATAC, we created a cell-by-bin matrix with 5 kb bin size for each sequencing depth and performed clustering using the default parameters in snapATAC. The clustering accuracy was evaluated using the normalized mutual information (NMI), with 0 representing no mutual information and 1 representing perfect match between two different labels, and was calculated using the “aricode” [80] package in R.

### Clustering evaluation based on the published scATAC-seq dataset

We evaluated the clustering performance on public scATAC-seq datasets from 7 mixed cell lines (GSE65360 from GEO), cells from HSC lineages (GSE74310 and GSE96772 from GEO), and scATAC-seq from 10k PBMC cells [81]. All the datasets were processed using the MAESTRO workflow from the fastq files. Cells with less than 1000 unique fragments or less than 10% of promoter enriched reads were removed from the analysis. For scABC, we set *k* = 10 for the *k*-means-based clustering. For cisTopic, the topic number used was 30. For LSI, the top 50 dimensions are used after TF-IDF conversion, and the clustering resolution was set to 0.6. For snapATAC, we created a cell-by-bin matrix with 5 kb bin size for each dataset and performed clustering using the default parameters in snapATAC. For 7 cell line mixed dataset and HSC differentiation dataset, the original cell-type labels were used to calculate the NMI. For the PBMC dataset, we adopted the Residual Average Gini Index (RAGI) score from a recent publication to evaluate the clustering performance [82]. We then calculated the averaged GINI index of marker genes from scRNA-seq between different clusters and compared them to the averaged GINI index calculated using housekeeping genes (https://m.tau.ac.il/~elieis/HKG/HK_genes.txt). The difference of the averaged GINI index between marker genes and housekeeping genes was defined as RAGI scores.

### Analysis of 10X Genomics PBMC scRNA-seq and scATAC-seq dataset

The PBMC 12K scRNA-seq dataset and 10K scATAC-seq dataset from different donors (Dataset #1) were downloaded from the 10X Genomics website (https://support.10xgenomics.com/single-cell-gene-expression/datasets/2.1.0/pbmc8k, https://support.10xgenomics.com/single-cell-gene-expression/datasets/2.1.0/pbmc4k, https://support.10xgenomics.com/single-cell-atac/datasets/1.1.0/atac_v1_pbmc_10k). We merged the two scRNA-seq datasets together and confirmed that there is no significant batch effect between these two datasets. The PBMC 2K scRNA-seq and 10K scATAC-seq dataset from the same donor (dataset #2) were generated and shared by 10X Genomics. We processed all the PBMC datasets from fastq files using MAESTRO, with all parameters set to default. After clustering, we first annotated the cell type automatically using the LM22 gene signature from CIBERSORT, then manually corrected the annotation result and added annotations for some of the rare populations like pDC.

### Analysis of 10X Genomics BMMC scRNA-seq and scATAC-seq dataset

The 5K scRNA-seq and 9K scATAC-seq of BMMCs from a healthy donor and a CLL patient were generated and shared by 10X Genomics (dataset #3). We converted the bam files to fastq files using the bamtofastq function from CellRanger and processed the dataset using MAESTRO with all default parameters. To get a comprehensive understanding of the immune microenvironment change between the healthy donor and the CLL patient, we merged the scRNA-seq from the BMMC healthy donor and the CLL patient, and also merged the corresponding scATAC-seq datasets, and the ran MAESTRO on the merged dataset using RNA module and ATAC module, respectively. Then, we integrated the clusterings from RNA and ATAC analyses. The scRNA-seq clusters were first annotated using LM22 gene signatures; however, as the BMMC microenvironment contains plenty of premature B cells, we further manually checked the expression of SELL, MME, CD19, MS4A1, CD79A, IGHM, and IGHD and modified the misannotated cell types manually. By default, MAESTRO identifies driver regulators for each scRNA-seq and scATAC-seq clusters. To identify the differential regulators from CLL1 and CLL2 clusters, we also used MAESTRO to identify the regulators based on differential expressed genes and differential peaks between CLL1 and CLL2 clusters.

### Analysis of 10X Genomics basal cell carcinoma scRNA-seq and scATAC-seq dataset

We downloaded the processed expression matrix of 53K scRNA-seq from BCC microenvironment from the GEO database (GSE123814). Then, we performed the clustering analysis using MAESTRO with default parameters. The cell types for the scRNA-seq clusters were annotated using the meta-information provided in the original study. The BCC 38K scATAC-seq fragments and peak files were downloaded from the GEO database (GSE129785). We then lifted over the fragments and peaks from hg19 to hg38 and calculated the binarized peak count matrix based on the fragments and peak files. The gene activity scores were calculated using TSS 10K “enhanced RP model” in MAESTRO. Before clustering, we first filtered out the peaks only present in less than 50 cells and cells with less than 500 peaks. After clustering, we performed differential analysis and annotated the cell types using the annotations from the original study [83]; we integrated the scATAC-seq with scRNA-seq using MAESTRO and transferred the cell-type labels from scRNA-seq to scATAC-seq.

### Evaluation of cell-type annotation performance using MAESTRO, Garnett, and SCINA

We used the published sorted PBMC dataset from Zheng et al. to evaluate the annotation performance of cell-type annotation [16]. The dataset was downloaded and downsampled from a recent evaluation study [84], which guaranteed each cell type has exactly 2000 cells. For SCINA, we first performed quantile normalization on the log-transformed expression matrix and annotated the dataset with the LM22 gene signature. For Garnett, we performed 5-fold cross-validation to train the classifier with both the LM22 gene signature and a simple immune cell-type signature from the Garnett package. For MAESTRO, we clustered all the cells with default parameters and annotated them using the LM22 gene signature. We adopted the median F1-score from Abdelaal et al., which measures the test accuracy by 2 × precision × recall/(precision + recall). All cell types, including both NK, T cell, B cell, monocyte, and granulocyte, were used to calculate the median F1-score.

### Evaluation of integration results from MAESTRO, Seurat, SnapATAC, and cicero

We integrated scRNA-seq and scATAC-seq using gene body accessibility score from snapATAC, co-accessibility gene score from cicero, promoter and gene body accessibility score from Seurat, and gene regulatory potential (TSS 10K “enhanced RP model”) from MAESTRO. All the integration analyses were performed using the CCA-based method and default parameters. We evaluate the integration performance from two aspects. The first aspect is to evaluate the performance of cell alignment between scRNA-seq and scATAC-seq. After label transferring using Seurat v3 [28], we compared the distribution of the maximal label prediction scores for the integration using four different methods. Cells with a maximal prediction score higher than 0.5 are defined as having a high-quality prediction. Cells with low prediction scores were removed from the downstream analysis. We then compared the number of cells with high-quality prediction in each cluster using four different methods, to evaluate how confidently each method can align scATAC-seq with scRNA-seq (Additional file 7: Table 5). Another aspect is the consistency between gene expression level from scRNA-seq and gene activities from scATAC-seq. We grouped the scRNA-seq and scATAC-seq cells based on the cell-type annotation from scRNA-seq and calculated the averaged gene expression in scRNA-seq and averaged scATAC-seq gene accessibility scores for scATAC-seq for each gene. We then calculated the genome-wide Spearman’s correlation coefficients between gene expression levels and gene accessibility levels, and the correlation reflected whether the gene activity score from scATAC-seq is a confident predictor of gene expression. In addition, we also calculated the averaged gene expression and gene accessibility score only on the top 2000 variable genes which were used in the integration analysis. We evaluated the consistency using Spearman’s correlation coefficient. The *p* value of the correlation was determined by the cor.test in R.

## Supplementary information

**Additional file 1: Table S1.** Components of MAESTRO that achieved using the existing software and custom code.

**Additional file 2: Table S2.** Running time and memory comparison between MAESTRO and other tools for scATAC-seq analysis.

**Additional file 3.** Supplementary figures.

**Additional file 4.** Supplementary materials.

**Additional file 5: Table S3.** Immune and stromal gene signatures used in the analysis

**Additional file 6: Table S4.** Transcription factor motif clusters used in MAESTRO workflow

**Additional file 7: Table S5.** Number of cells in scATAC-seq that received transferred labels from scRNA-seq.

**Additional file 8.** HTML output for the scRNA-seq analysis on the human PBMC sample (12k cells) from different donors using MAESTRO.

**Additional file 9.** HTML output for the scATAC-seq analysis on the human PBMC sample (10k cells) from different donors using MAESTRO.

**Additional file 10.** HTML output for the integrated analysis of scRNA-seq (12k cells) and scATAC-seq (10k cells) datasets of human PBMC from different donors using MAESTRO.

**Additional file 11.** Review history.

## Data Availability

The MAESTRO package is freely available under the GPL-3.0 license. The source code of MAESTRO can be found at the GitHub repository (https://github.com/liulab-dfci/MAESTRO) [85] and Zenodo with the access code DOI: 10.5281/zenodo.3862812 [86]. We also provide a docker version of the package at https://hub.docker.com/r/winterdongqing/maestro. The accession numbers for the public dataset used in this study include GSE65360, GSE74310, GSE96772, GSE123814, and GSE129785. Other public datasets are downloaded from 10X Genomics website (https://support.10xgenomics.com/single-cell-gene-expression/datasets/2.1.0/pbmc8k, https://support.10xgenomics.com/single-cell-gene-expression/datasets/2.1.0/pbmc4k, https://support.10xgenomics.com/single-cell-atac/datasets/1.1.0/atac_v1_pbmc_10k). Additional benchmark code used in this paper is deposited at the GitHub repository (https://github.com/chenfeiwang/MAESTRO_benchmark) [87] and Zenodo with the access code DOI: 10.5281/zenodo.3953145 [88].
