# Supplementary material for: Integrative analyses of single-cell transcriptome and regulome using MAESTRO
Source: Genome Biol. 2020 Aug 7;21:198. doi: 10.1186/s13059-020-02116-x (PMC7412809; doi:10.1186/s13059-020-02116-x)
Supplement: Supplementary file 3 — Additional file 3. Supplementary figures. [file 13059_2020_2116_MOESM3_ESM.docx]

**Supplementary Figures**

**Fig S1. Quality control of human PBMC scRNA-seq and scATAC-seq dataset**

(**a**). Phred quality score of scRNA-seq reads. The x-axis represents the position of reads from 5’ to 3’.

(**b**). GC content distribution of scRNA-seq reads.

(**c**). Nucleotide frequency plot of scRNA-seq reads. The x-axis represents the position of reads from 5’ to 3’.

(**e**). Reads distribution of gene body for scRNA-seq. All RefSeq genes are separated into 100 percentile and aligned together to generate this figure.

(**f**). Fragment size distribution for 1% reads sampled from scATAC-seq. Only fragments with insert size less than 1,000 are considered. Y-axis represents the number of fragments.

**Fig S2. Regulatory potential score for modeling gene activity in scATAC-seq**

Schema of the regulatory potential model in MAESTRO. For each gene, we weighed the presence of ATAC-seq peaks around its TSS by an exponential decay function on the distance of peak center to TSS, with 10kb as the half-decay distance. Peaks present within the exon region of the candidate gene (Gene A) were given a weight of 1 and further normalized by total exon length of the gene. Peaks located in the nearby gene promoter or exon regions (Gene B) were removed from the score calculation. The weighted scores for each peak (green bar in the figure) are summed to generate the regulatory potential score for the candidate gene.

**Fig S3. Cell-type annotation of human PBMC scRNA-seq and scATAC-seq dataset**

**
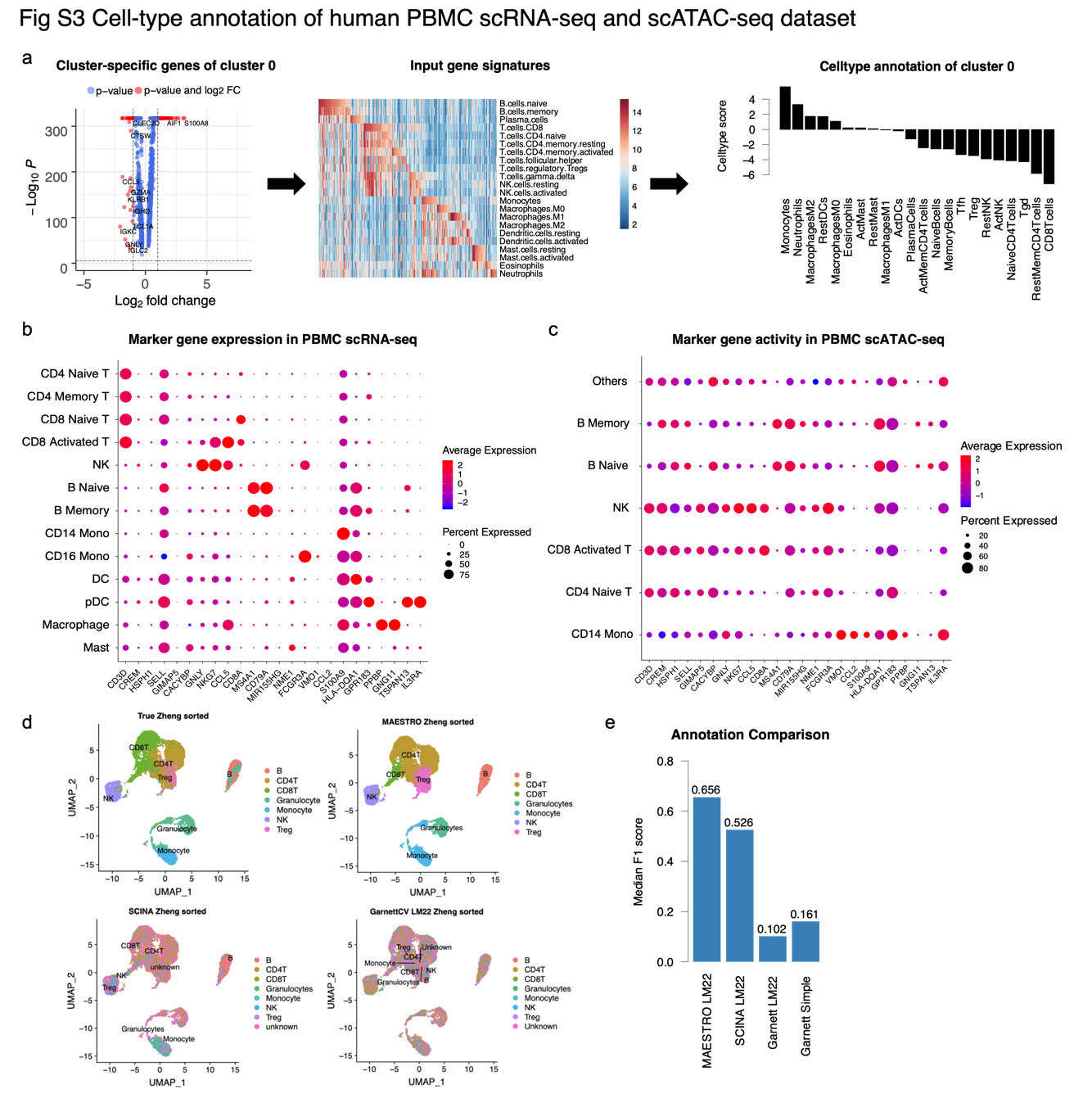
**

(**a**). Chart illustrating the cell-type annotation in MAESTRO. (1) For one cluster from the scRNA-seq dataset, we derived the differentially expressed genes (both positive and negative markers) for cells in this cluster versus other cells in the dataset. (2) Given the signature of different cell-types, we calculated the averaged logFC for each cell-type based on the logFC of the markers in one cluster, as the cell-type scores for that cluster. (3) After we calculated the cell-types scores, we ranked all of the scores and used the cell-type with the highest score as the identity for that cluster. If the highest cell-type score is below a certain threshold (less than 0), the cluster will be annotated as “Others”.

(**b**). Dot plot showing the marker gene expression level in scRNA-seq clusters. The color of the circles indicates the averaged expression level, and size represents the fraction of cells with the gene expressed.

(**c**). Dot plot showing the regulatory potential score of marker genes in scATAC-seq clusters. The color of the circles indicates the averaged regulatory potential level, and size represents the fraction of cells that have a non-negative log2 regulatory potential level.

(**d**). Comparison of the performance on automatic cell-type annotation using a dataset from sorted PBMCs from Zheng et al. UMAP visualization of true labels, MAESTRO annotated labels, SCINA annotated labels and GarnettCV annotated labels on sorted PBMC dataset.

(**e**). Median F1-score for cell-type annotation using MAESTRO, SCINA, GanettCV on LM22 signatures, and GanettCV on the default gene signatures.

**Fig S4. Clustering of transcription factors based on motif similarity and expression level**

(a). Hierarchical clustering of the human AP1 transcription factor family. Colors represent the information content weighted Pearson correlation coefficients between different motif PWM models. Motif logos of ATF2 and MAFK are shown to demonstrate the similarity between different PWM models.

(b). Hierarchical clustering of 769 human PWM models representing 680 transcription factors.

(c). The rank of driver transcription regulators in the CD8 activated T-cells of PBMC scRNA-seq. The regulators are ranked by the TF enrichment score from LISA, and the color of circles represents the averaged expression level of corresponding regulators in CD8 T-cells. The names of the top 10 TFs are labeled on the graph.

(d). Dot plot showing the gene expression level of STAT1, STAT3, STAT4, STAT5A, STAT5B, and BCL6 in scRNA-seq clusters. The color of the circles indicates the averaged expression level, and size represents the fraction of cells with the gene expressed.

**Fig S5. Integrative analysis of scRNA-seq and scATAC-seq enables the identification of rare elements in rare populations**

**
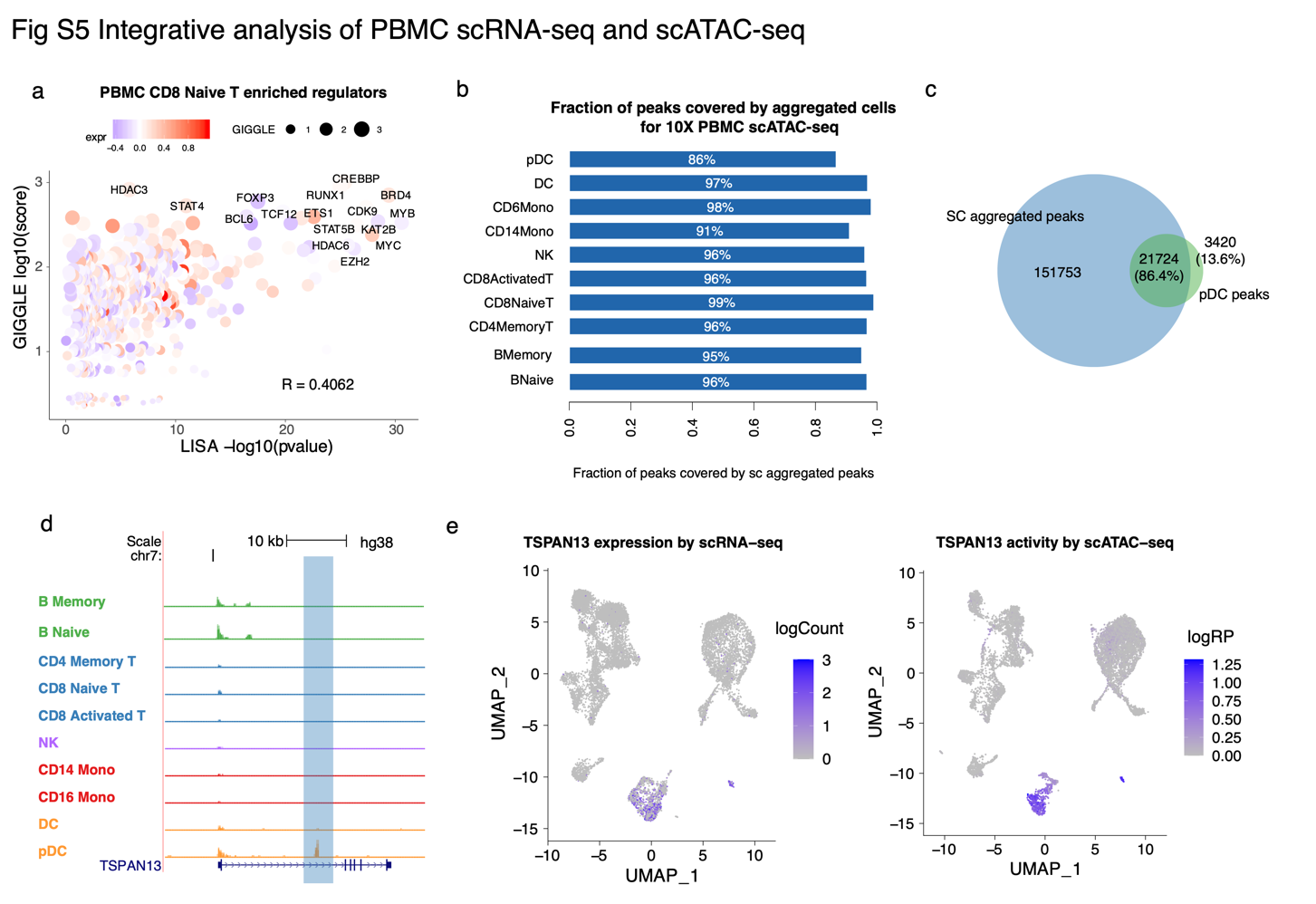
**

(a). The rank of driver regulators in CD8 Naïve T cells of the PBMC dataset. The x-axis represents the TF enrichment score from LISA results in cluster-specific genes using scRNA-seq; the y-axis represents the TF enrichment score from GIGGLE results in cluster-specific peaks using scATAC-seq. The color of circles represents the averaged expression level of corresponding regulators in CD14 monocyte scRNA-seq cells, and size represents the TF enrichment score using GIGGLE in CD8 Naïve T scATAC-seq cells. The names of the top 10 TFs from LISA and GIGGLE are labeled on the graph.

(b). Barplot showing the fraction of peaks in each cell-type cluster that can be covered by the peaks called from the single-cell aggregated dataset.

(c). The overlap between single-cell aggregated peaks and peaks called from pDC clusters, which is the aggregation of scATAC-seq cells only from the pDC cluster.

(d). Genome browser view of TSPAN13 locus. The *pseudo*-bulk ATAC-seq profiles are generated by pooling together cells within each cell-type. Y-axis represents sequence depth normalized ATAC-seq signals (reads per million mapped reads, RPM).

(e). Left, UMAP visualization of TSPAN13 expression in scRNA-seq cells. Right, UMAP visualization of TSPAN13 gene regulatory potential in scATAC-seq cells.

**Fig S6. Integration of BMMC scRNA-seq and scATAC-seq from CLL patients and healthy donors**

(a). Dot plot showing the marker gene expression level in scRNA-seq clusters. The color of the circles indicates the averaged expression level, and size represents the fraction of cells with the gene expressed.

(b). Dot plot showing the regulatory potential score of marker genes in scATAC-seq clusters. The color of the circles indicates the averaged regulatory potential level, and size represents the fraction of cells that have a non-negative log2 regulatory potential level.

(c). UMAP visualization of IGHM expression in the BMMC scRNA-seq (5k cells) dataset.

(d). UMAP visualization of RGS1 expression in the BMMC scRNA-seq (5k cells) dataset.

(e-f). The rank of driver regulators in naïve B-cells and pre-pro B-cells of the BMMC dataset. The x-axis represents the TF enrichment score from LISA results in cluster-specific genes using scRNA-seq; the y-axis represents the TF enrichment score from GIGGLE results in cluster-specific peaks using scATAC-seq. The color of circles represents the averaged expression level of corresponding regulators in naïve B-cells or pre-pro B-cells of scRNA-seq, and size represents the TF enrichment score using GIGGLE in naïve B-cells or pre-pro B-cells of scATAC-seq. The names of the top 10 TFs from LISA and GIGGLE are labeled on the graph.

**Fig S7. Integrative analysis of human PBMC scRNA-seq and scATAC-seq using snapATAC-seq, cicero, and Seurat**


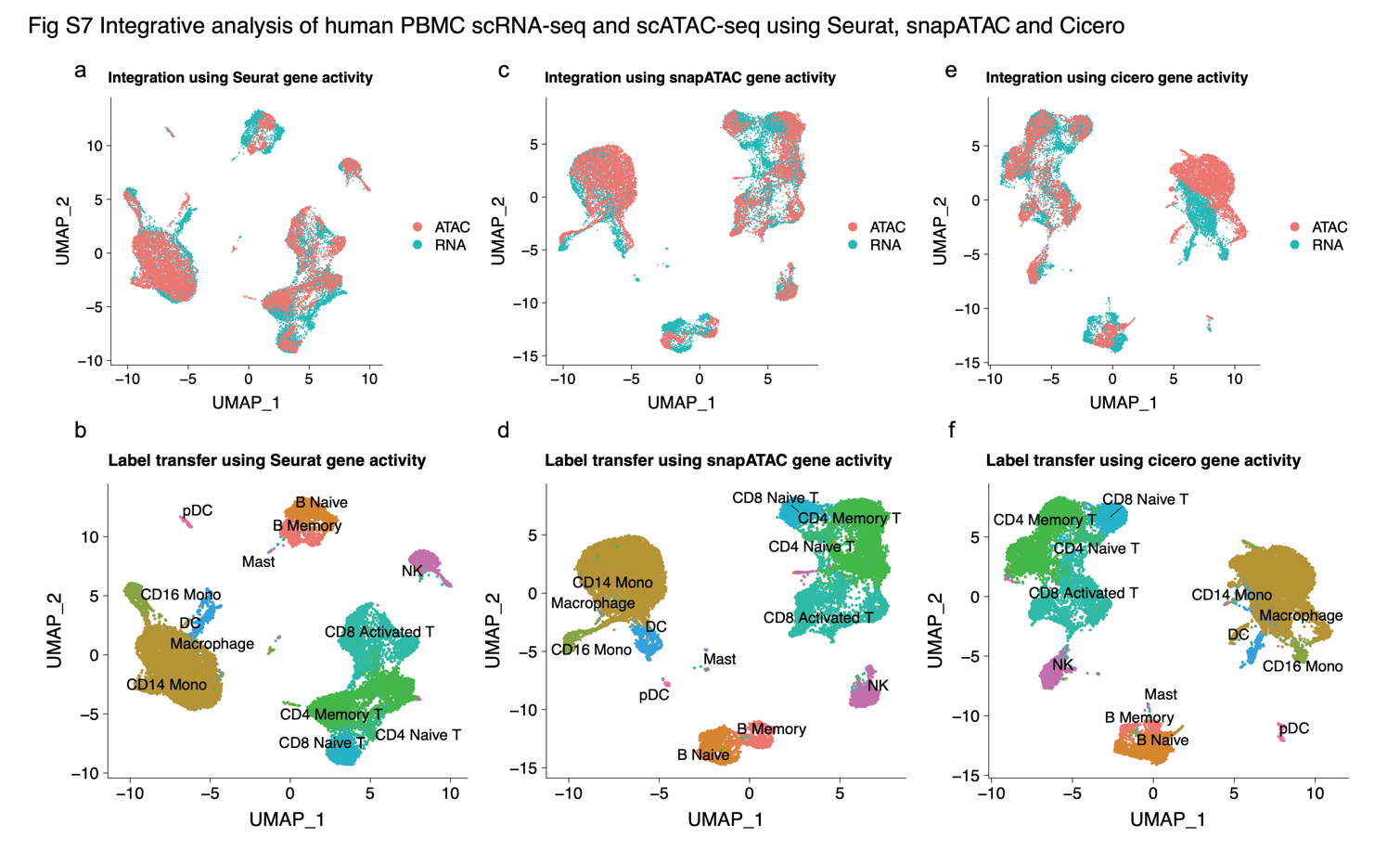


(**a**). UMAP visualization for joint clustering of human PBMC (12k cells) scRNA-seq and scATAC-seq (10k cells). The cells are joined by CCA on gene expression level and Seurat gene score. Colors represent cells from different technologies.

(**b**). UMAP visualization for joint clustering of human PBMC (12k cells) scRNA-seq and scATAC-seq (10k cells). The cells are joined by CCA on gene expression level and Seurat gene score. Colors represent the cell-types, for which is generated using the scRNA-seq dataset and transferred to the scATAC-seq dataset.

(**c**). UMAP visualization for joint clustering of human PBMC (12k cells) scRNA-seq and scATAC-seq (10k cells). The cells are joined by CCA on gene expression level and snapATAC gene score. Colors represent cells from different technologies.

(**d**). UMAP visualization for joint clustering of human PBMC (12k cells) scRNA-seq and scATAC-seq (10k cells). The cells are joined by CCA on gene expression level and snapATAC gene score. Colors represent the cell-types, for which is generated using the scRNA-seq dataset and transferred to the scATAC-seq dataset.

(**e**). UMAP visualization for joint clustering of human PBMC (12k cells) scRNA-seq and scATAC-seq (10k cells). The cells are joined by CCA on gene expression level and cicero gene activity score. Colors represent cells from different technologies.

(**f**). UMAP visualization for joint clustering of human PBMC (12k cells) scRNA-seq and scATAC-seq (10k cells). The cells are joined by CCA on gene expression level and cicero gene activity score. Colors represent the cell-types, for which is generated using the scRNA-seq dataset and transferred to the scATAC-seq dataset.

**Fig S8. Comparison of integration performance of MAESTROl Seurat, snapATAC and cicero**


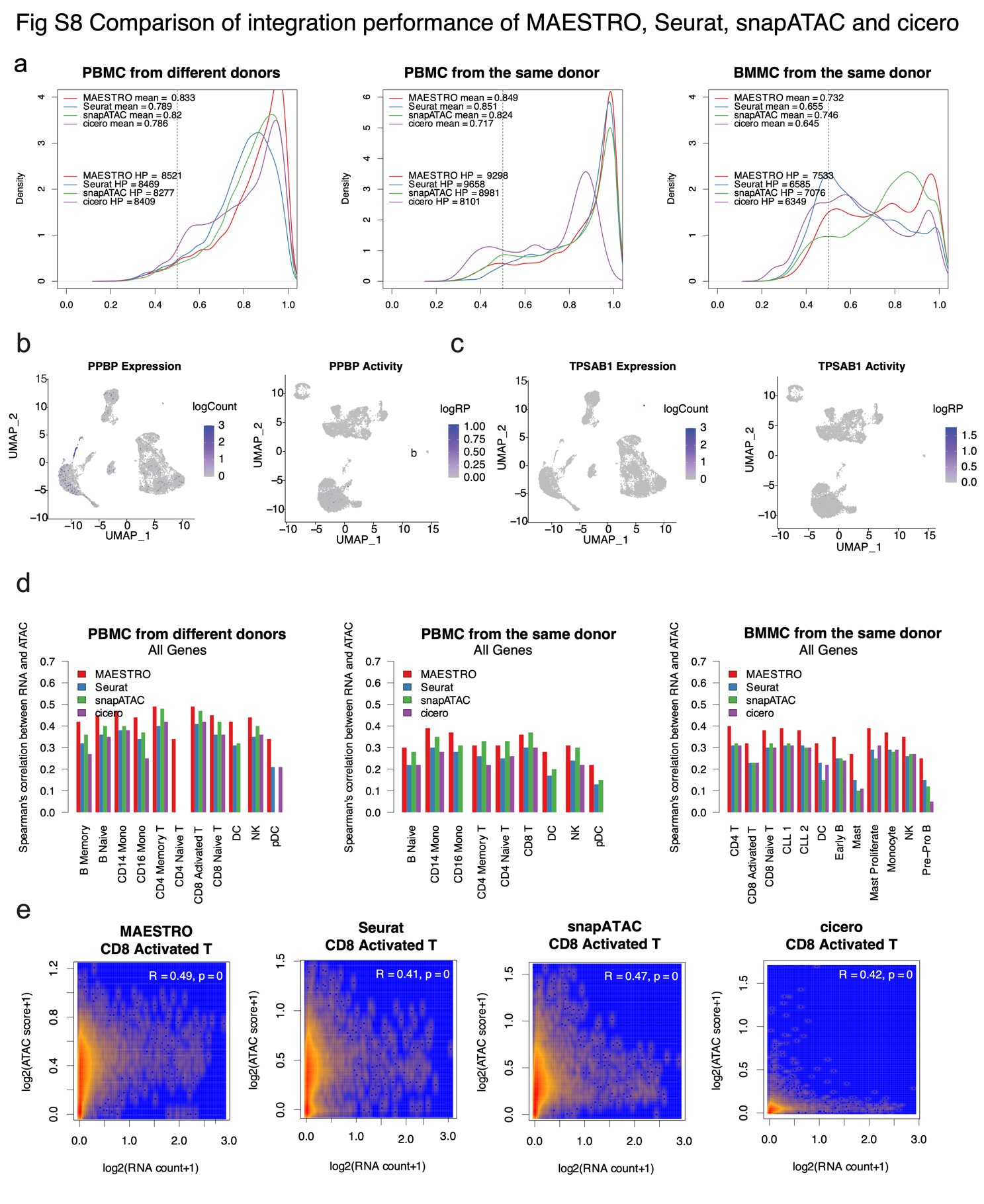


(**a**). Distribution of the cell-type label prediction score after integration of the scRNA-seq and scATAC-seq using gene activity scores from MAESTRO, Seurat, snapATAC and cicero. The analyses were made on three independent datasets: PBMC from different donors, PBMC from the same donor and BMMC from the same donor. For each dataset, the mean of cell-type label prediction score and the number of cells that have a high-quality prediction (score > 0.5, termed as HP) are labeled on the figure.

(**b**). Umap plot showing the gene expression from scRNA-seq and gene activity from scATAC-seq for Macrophage marker PPBP.

(**c**). Umap plot showing the gene expression from scRNA-seq and gene activity from scATAC-seq for Mast marker TPSAB1.

(**d**). Comparison of the consistency between gene expression and gene activity scores from MAESTRO, Seurat, snapATAC, and cicero. The comparisons were made on three independent datasets. The x-axis represents different cell-types, and the y-axis represents the Spearman’s correlation coefficient between expression level from scRNA-seq and gene activity score from scATAC-seq within each cluster. All the genes were used in the analysis.

(**e**). Scatter plot for visualizing the average gene expression level and average gene activity score for all genes in CD8 activated T-cells from PBMC data from the different donors. The x-axis represents the log2 UMI count from scRNA-seq, the y-axis represents the log2 gene activity score from scATAC-seq.

**Fig S9. Integration of scRNA-seq and scATAC-seq from the BCC microenvironment**


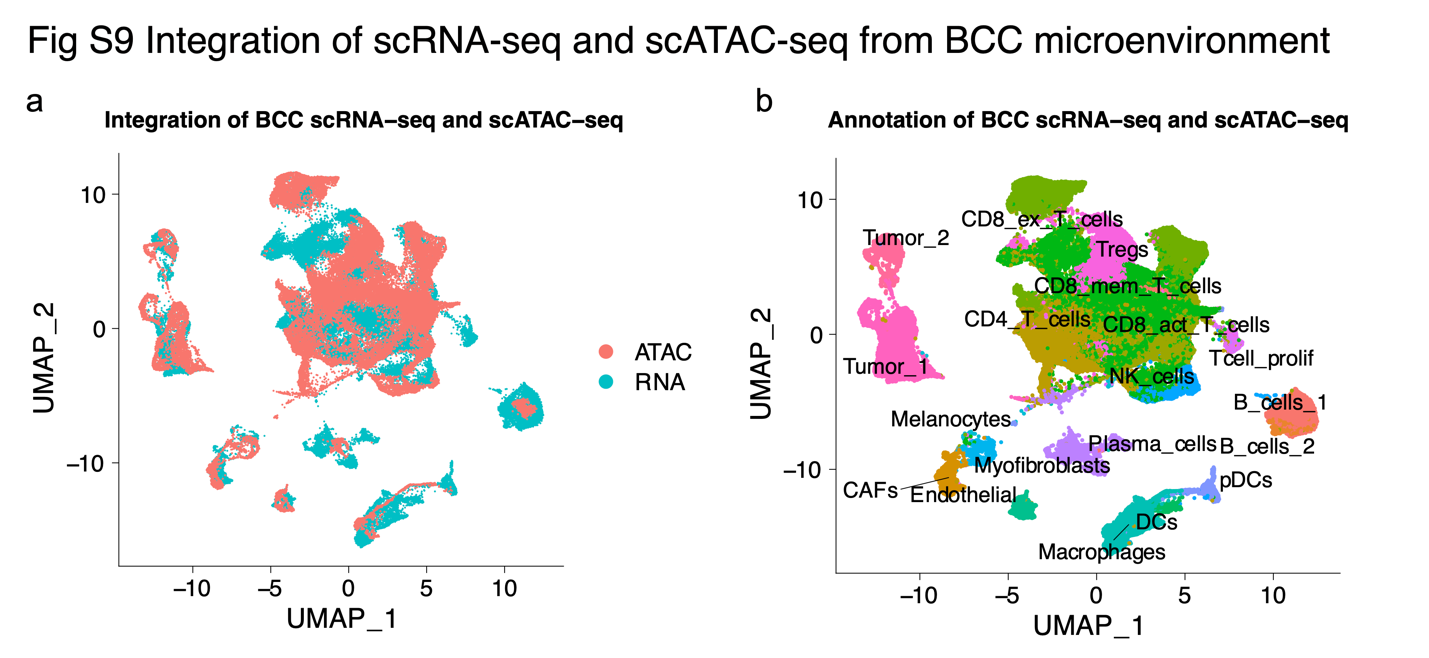


(**a**). UMAP visualization for joint clustering of human BCC scRNA-seq (53k cells) and scATAC-seq (38k cells). Colors represent cells from different technologies. The cells are joined by CCA on gene expression level and regulatory potential from MAESTRO.

(**b**). UMAP visualization for joint clustering of human BCC scRNA-seq (53k cells) and scATAC-seq (38k cells). The cells are joined by CCA on gene expression level and regulatory potential from MAESTRO. Colors represent the cell-types, for which is generated using the scRNA-seq dataset and transferred to the scATAC-seq dataset.
