# Supplementary material for: Integrative analyses of single-cell transcriptome and regulome using MAESTRO
Source: Genome Biol. 2020 Aug 7;21:198. doi: 10.1186/s13059-020-02116-x (PMC7412809; doi:10.1186/s13059-020-02116-x)
Supplement: Supplementary file 4 — Additional file 4. Supplementary materials. [file 13059_2020_2116_MOESM4_ESM.docx]

**Supplementary Materials**

**A. Comparing the performance of different clustering methods and peak sets for scATAC-seq**

To select the best practice for scATAC-seq clustering, we compared the clustering performance of four published methods including scABC, latent semantic indexing followed by graph-based clustering (termed LSI here), cisTopic followed by density-based clustering (termed cisTopic here), and snapATAC. To evaluate the ability to process sparse data, we first simulated a set of scATAC-seq datasets from 10 public bulk ATAC-seq datasets of different cell types with different coverages (from 1,000 to 10,000 reads/cell). To measure the agreement with the original cell types, we used Normalized Mutual Information (NMI), with 0 representing no mutual information and 1 representing perfect match between clusters from an algorithm and from the original cell types. Comparisons on simulated data showed that scABC is highly sensitive to sequencing depth, while LSI is the most robust method even at low coverage (Figure M1).


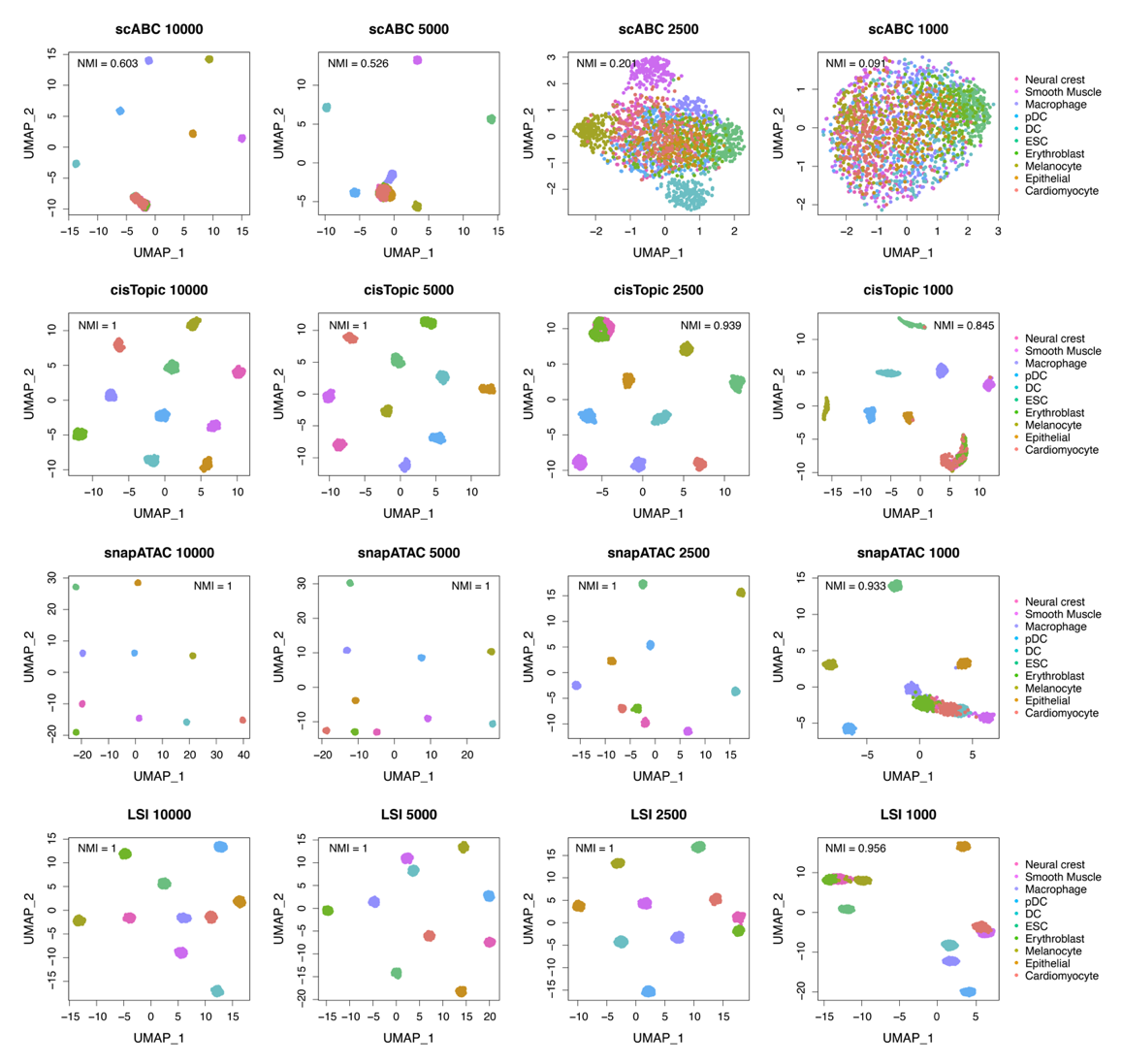


Figure M1. Benchmark of scATAC-seq clustering performance using a simulated dataset

Comparison of scATAC-seq clustering performance using scABC, cisTopic, snapATAC, and LSI on a simulated dataset. For each sequencing depth, we simulated 2,000 (200 x 10) single-cells from 10 bulk ATAC-seq datasets. Colors indicate different cell types. pDC, plasmacytoid dendritic cell; DC, dendritic cell; ESC, embryonic stem cell.

We further evaluated the clustering performance of these algorithms on the published scATAC-seq datasets from different experimental platforms (Figure M2). We first assessed microfluidic-based scATAC-seq with known cell type labels. SnapATAC and LSI have the best accuracy on the scATAC-seq dataset with seven cell lines, indicating their ability to distinguish overall lineage differences (Figure M2a, b). LSI and cisTopic can better discriminate differentiated cells from hematopoietic stem cells (HSC), suggesting a better sensitivity for detailed clustering structures and rare populations from the same lineage (Figure M2c, d). Although scATAC-seq from barcoded technologies such as 10X Genomics does not provide cell-type annotation, a recent publication successfully utilized the Residual Average Gini Index (RAGI) score to evaluate cluster separations by measuring whether cell type marker genes showed higher heterogeneity (or higher GINI index) between clusters than housekeeping genes (see Methods). Using the RAGI metric on the human 10K PBMC scATAC-seq dataset from 10X Genomics suggested significant advantages of LSI compared with other methods (Figure M2e, f). Based on these comparisons, LSI has the overall best performance in terms of robustness and clustering accuracy.


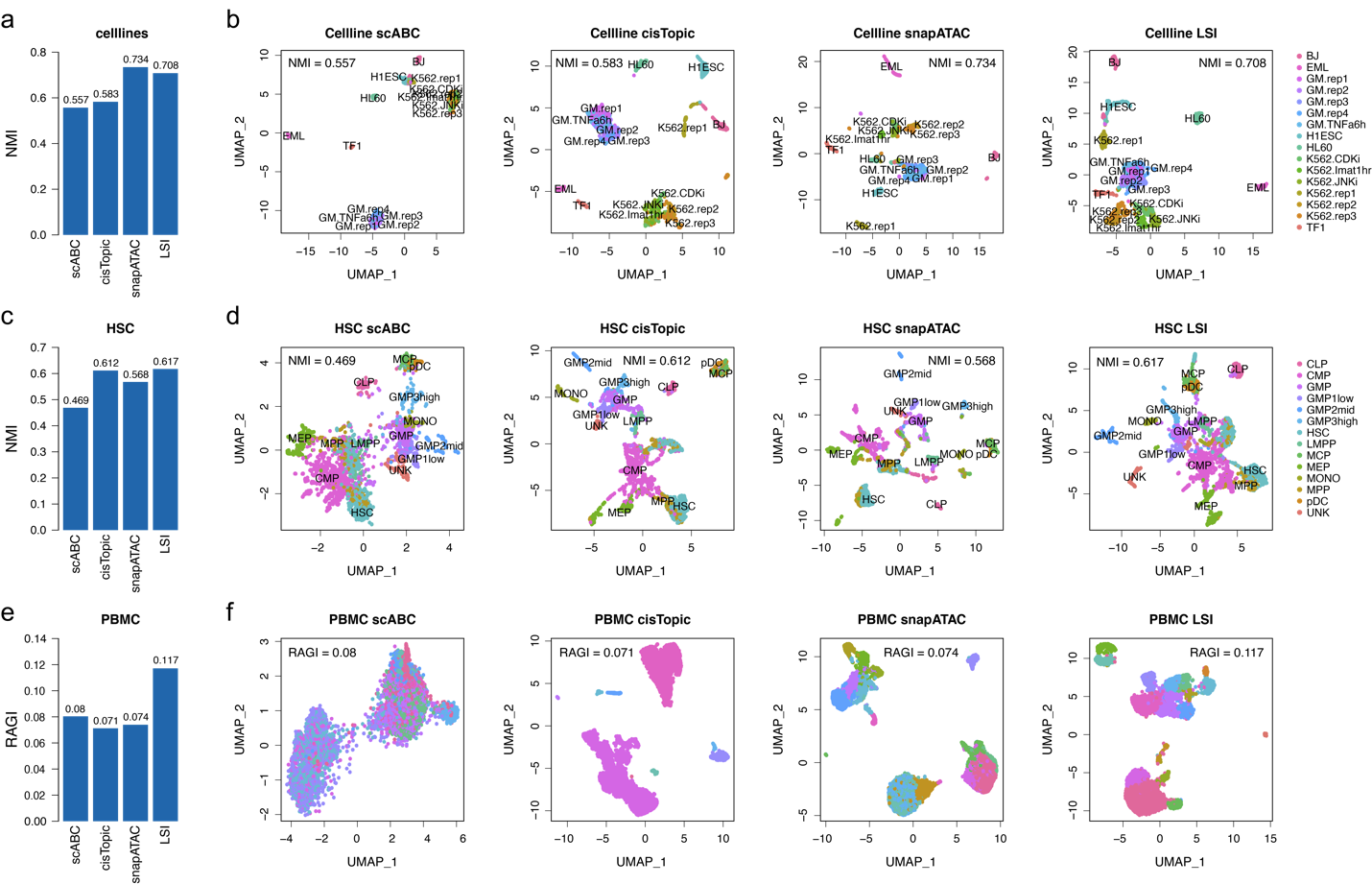


Figure M2. Benchmark of scATAC clustering performance on public datasets

(a). NMI comparison of scABC, cisTopic, snapATAC, and LSI on the 7-cell-line mixed scATAC-seq dataset.

(b). UMAP visualization of the clustering result using scABC, cisTopic, snapATAC and LSI on the 7-cell-line mixed scATAC-seq dataset. Colors represent different cell types.

(c). NMI comparison of scABC, cisTopic, snapATAC, and LSI on the HSC scATAC-seq dataset.

(d). UMAP visualization of the clustering result using scABC, cisTopic, snapATAC and LSI on HSC scATAC-seq dataset. Colors represent different cell types.

(e). RAGI comparison of scABC, cisTopic, snapATAC, and LSI on human PBMC scATAC-seq (10k cells) dataset.

(f). UMAP visualization of the clustering result using scABC, cisTopic, snapATAC and LSI on the PBMC scATAC-seq (10k cells) dataset. Colors represent different clusters.

MAESTRO clusters the cells in scATAC-seq using binary read count in the peaks called from aggregated signals, which might be biased towards major populations in the sample. To remove the potential bias, we added a function for clustering using candidate cis-regulatory elements (CCRE) catalog from the ENCODE project, which covers cis-elements from most of the known cell-types and tissues. Clustering on additional cis-elements indeed improved the clustering accuracy for the dataset containing multiple lineages (Figure M3a, b). However, when the data contained cells from the same lineage, adding cis-elements from other lineages reduced the clustering accuracy, probably due to the increased noise from unrelated CCRE (Figure M3c, d). Therefore, MAESTRO performs the clustering on peaks from cell aggregations by default, with the option to use user-defined cis-elements such as CCRE.


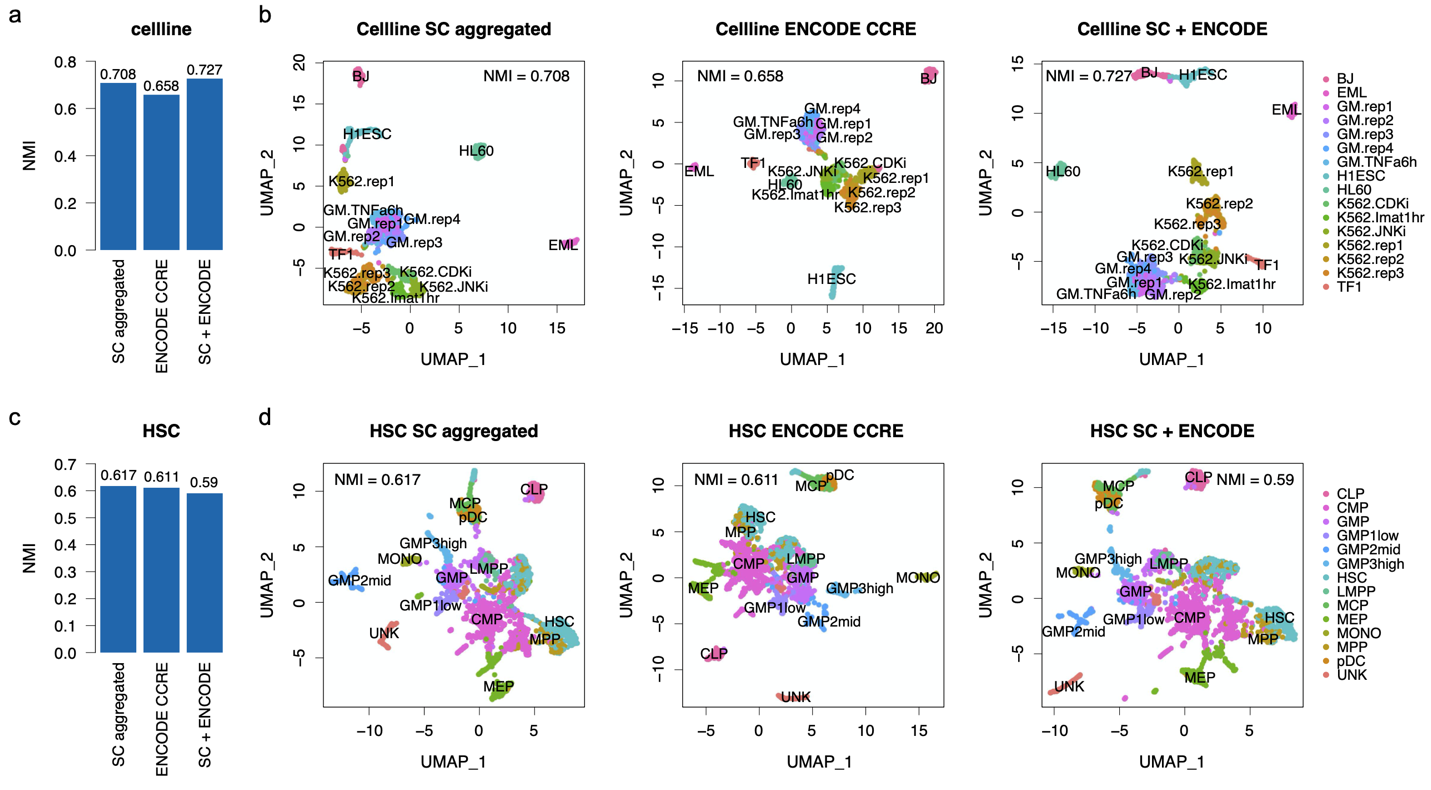


Figure M3. Benchmark of scATAC-seq clustering using different peak sets

(a). NMI comparison of clustering using (1) peaks called from single-cell aggregated samples (2) peaks from ENCODE representative DHS sites (3) merged peaks from single-cell aggregated samples and ENCODE rDHS on 7-cell-line mixed scATAC-seq samples.

(b). UMAP visualization of the clustering result using different sets of peaks on the 7-cell-line mixed scATAC-seq dataset. Colors represent different cell types.

(c). NMI comparison of clustering using different sets of peaks on the HSC scATAC-seq dataset.

(d). UMAP visualization of the clustering result using different sets of peaks on the HSC scATAC-seq dataset. Colors represent different cell types.

**B. Evaluate the integration performance of different MAESTRO regulatory potential models**

We modeled the gene activity from scATAC-seq using the regulatory potential (RP) model. The presence of scATAC-seq peaks surrounding each gene reflects the potential transcription regulator (TR) bindings and their impact on the gene expression. Our regulatory potential model assumes that the influence of a peak on the expression of a gene decreases monotonically as a function of distance to the TSS, and the influence of multiple peaks are addictive (Figure M4a). We consider a smooth decay function to be a more reasonable model than a binary distance cut-off, as Hi-C chromatin interaction data demonstrated the decreased interaction frequency with increased genomic proximity (Fig 2A in Lieberman-Aiden et al, Science 2009). We termed this naïve RP model as “simple RP model”. In scATAC-seq datasets, we observed that the exon region of a gene can also be accessible, which might be caused by the sliding of RNA Pol II transcriptional complex during transcription. In addition, we noticed that unexpressed genes may be incorrectly assigned with gene regulatory potential scores due to the strong promoter and exon signals from nearby expressed genes. Based on these considerations, we have modified the RP model, by giving the peaks presented in the exon region an equal weight to TSS (weighted as 1 and further normalized by the exon length, Gene A in Figure M4a), and removing the peaks located in nearby promoters and exons (Gene B in Figure M4a) when calculating RP for Gene A. We termed the modified RP model as “enhanced RP model”.

To demonstrate our regulatory potential model could better predict the gene expression level at single-cell level, we calculated the RP for three independent datasets using different models (Figure M4b), and compared the performance after integrating with corresponding scRNA-seq datasets. Our analyses showed that after integration, the “enhanced RP model” has the highest label prediction score compared to other models (Figure M4b, model 8, pink group versus all other groups), which indicates the cell-type labels from scRNA-seq were transferred more appropriately. In addition, we have compared the consistency of gene activity with gene expression for each cell-type cluster, and the “enhanced RP-model” has an overall higher consistency evaluated using spearman correlation coefficient on three different datasets (Figure M4c, model 8, pink group versus all other groups). These results suggest that the “enhanced RP model” has better performance in both integrating scATAC-seq with scRNA-seq, and predicting potential gene expression from scATAC-seq. In the MAESTRO workflow, we include both the “simple RP model” and “enhanced RP model”, and use the “enhanced RP model” as the default gene activity scoring model.

Figure M4. Comparison of scRNA-seq and scATAC-seq integration performance using different regulatory potential models.

(a) Schema of the new regulatory potential model in MAESTRO. For each gene, we weighed the presence of ATAC-seq peaks around its TSS by an exponential decay function on its distance to TSS, with 10kb as half-decay distance to both directions. Peaks present within the exon region of the candidate gene (Gene A) were given a weight of 1 and further normalized by exon length. Peaks located in the nearby gene promoter or exon regions (Gene B) were removed from the score calculation. The weighted scores for each peak (green bar in the figure) are summed to generate the regulatory potential score for the candidate gene.

(b) Distribution of cell-type label transferring score for different RP models, listed as follows. The 8^th^ model is the default RP model used in MAESTRO.

1: TSS-centered RP, simple RP model;

2: Gene-centered RP;

3: TSS-centered RP excluding peaks in adjacent gene promoters;

4: TSS-centered RP excluding peaks in adjacent gene promoters and exons;

5: TSS-centered RP excluding peaks in adjacent gene promoters and gene-bodies;

6: TSS-centered RP with equal weights for peaks located in exon of candidate genes, normalized by total exon length;

7: TSS-centered RP with equal weights for peaks located in gene-body of candidate genes, normalized by gene body;

8: TSS-centered RP, with equal weights for peaks located in exon of candidate gene, normalized by exon length , excluding peaks in any other gene promoters and exons, enhanced RP model;

9: TSS-centered RP, with equal weights for peaks located in gene-bodies of candidate gene, excluding peaks in any other gene promoters and gene-bodies;

(c) Distribution of spearman’s correlation coefficient between RP score and gene expression for each cluster. Different models from Figure M4b are compared.
