# Supplementary material for: Integrative analyses of single-cell transcriptome and regulome using MAESTRO
Source: Genome Biol. 2020 Aug 7;21:198. doi: 10.1186/s13059-020-02116-x (PMC7412809; doi:10.1186/s13059-020-02116-x)
Supplement: Supplementary file 8 — Additional file 8. HTML output for the scRNA-seq analysis on the human PBMC sample (12k cells) from different donors using MAESTRO. [file 13059_2020_2116_MOESM8_ESM.html]

MAESTRO


MAESTRO

- scRNA-seq module
- scATAC-seq module
- Integration module

- Sample Information
- Quality Control
  - Bulk level
  - Single-cell level
- Cell Clustering
- Annotation
  - Celltype annotation
  - Regulator annotation

# Sample Information

|  |  |
| --- | --- |
| Sample ID | 10X\_PBMC\_12k |
| FASTQ Path | /home1/wangchenfei/Project/SingleCell/scRNA/Analysis/10X\_PBMC\_12k\_MAESTRO/Data/fastqs |
| Species | GRCh38 |
| Platform | 10x-genomics |

# Quality Control

## Bulk level

### Mappability

Read mappability and distribution of reads in coding regions (CDS) and intronic regions. In general, for human or mouse, **uniquely mapped reads** should account for more than 60% of all reads, and more reads should be distributed in exon regions than intron reads. La Manno et al (2018) found that for scRNA-seq, 15–25% of reads contained unspliced intronic sequences.

### Reads quality

Phred quality score of scRNA-seq reads. The x-axis represents the position of reads from 5’ to 3’.

### Reads nucleotide composition

Nucleotide frequency plot of scRNA-seq reads. The x-axis represents the position of reads from 5’ to 3’.

### GC content

GC content distribution of scRNA-seq reads.

### Gene body coverage

Reads distribution of gene body for scRNA-seq. Gene-body coverage evaluates sample read bias over 3’ or 5’ of the transcripts. For 10x-genomics, reads are supposed to be biased to 3' of the transcripts. For Smartseq2, reads are supposed to be distributed evenly over the transcripts.

## Single-cell level

### Cell filtering based on counts and covered genes

Cell filtering plot of scRNA-seq. The x-axis represents the number of unique reads/UMIs present in each cell, and the y-axis represents the number of genes covered in each cell.

# Cell Clustering

### Cell clustering based on gene expression

UMAP visualization of the clustering result. Colors represent different clusters with the cluster ID labeled.

# Annotation

## Celltype annotation

### Celltype annotation based on DE genes

UMAP visualization of annotated clusters. Colors represent different cell types. The cell type information for each cluster is annotated using the expression level of marker genes.

## Regulator annotation

### Cluster-specific regulator identified by LISA

Driver transcription regulators identified based on cluster-specific genes. By default, the regulators are ranked by the enrichment score in each cluster.

| Cluster | Celltype Annotation | Transcription Factor | log10(LISA score) |
| --- | --- | --- | --- |
| 0 | Monocytes | SPI1 | IRF1 | STAT1 | IRF8 | STAT2 | STAT3 | IRF2 | BCL6 | IRF3 | BCL11A | STAT5A | STAT5B | PRDM1 | IRF4 | SPIB | STAT4 | 137.68 |
| 0 | Monocytes | SMAD1 | 118.29 |
| 0 | Monocytes | CEBPB | CEBPD | ATF4 | CEBPG | CEBPA | NFIL3 | DDIT3 | BATF | DBP | BATF3 | CEBPE | HLF | 116.17 |
| 0 | Monocytes | KMT2A | 115.83 |
| 0 | Monocytes | BRD4 | 108.4 |
| 0 | Monocytes | RUNX1 | CBFB | RUNX3 | RUNX2 | 94.36 |
| 0 | Monocytes | JMJD1C | 93.43 |
| 0 | Monocytes | CDK8 | 88.31 |
| 0 | Monocytes | NCOR1 | 81.84 |
| 0 | Monocytes | ELF1 | FLI1 | ETV6 | ELF2 | ETS2 | ELK3 | ELK4 | NFAT5 | GABPA | ELK1 | ETV5 | ETS1 | ETV2 | ETV7 | ELF3 | EHF | ERG | 80.43 |
| 1 | NaiveCD4Tcells | BRD4 | 35.74 |
| 1 | NaiveCD4Tcells | MYB | MYBL1 | 33.77 |
| 1 | NaiveCD4Tcells | CDK9 | 30.46 |
| 1 | NaiveCD4Tcells | IRF1 | STAT3 | IRF3 | STAT1 | IRF2 | STAT5A | STAT4 | STAT5B | STAT2 | SPI1 | IRF8 | BCL11A | BCL6 | SPIB | IRF4 | PRDM1 | 30.12 |
| 1 | NaiveCD4Tcells | WDR5 | 27.41 |
| 1 | NaiveCD4Tcells | CREBBP | 25.96 |
| 1 | NaiveCD4Tcells | MAFK | MAFG | MAFF | NRL | MAF | MAFB | 25.61 |
| 1 | NaiveCD4Tcells | ZNF18 | TBX21 | EOMES | TBX19 | 25.21 |
| 1 | NaiveCD4Tcells | STAT6 | 25 |
| 1 | NaiveCD4Tcells | CBFB | RUNX1 | RUNX3 | RUNX2 | 24.63 |
| 10 | Monocytes | SPI1 | IRF1 | IRF8 | IRF2 | STAT3 | STAT1 | STAT2 | IRF3 | BCL6 | STAT5A | BCL11A | STAT5B | STAT4 | SPIB | IRF4 | PRDM1 | 144.2 |
| 10 | Monocytes | KMT2A | 104 |
| 10 | Monocytes | SMAD1 | 103.91 |
| 10 | Monocytes | BRD4 | 96.36 |
| 10 | Monocytes | ELF1 | FLI1 | ELF2 | ETS2 | ELK4 | ETV6 | NFAT5 | GABPA | ETS1 | ELK3 | ELK1 | ETV2 | ETV7 | ELF3 | ETV5 | 92.12 |
| 10 | Monocytes | RUNX3 | CBFB | RUNX1 | RUNX2 | 88.92 |
| 10 | Monocytes | NR4A2 | NR4A1 | RXRA | RARA | ESRRA | RXRB | VDR | THRA | NR2C1 | NR4A3 | NR1H3 | RARG | PPARD | PPARA | NR2F6 | NR1I3 | ESR1 | 88.44 |
| 10 | Monocytes | CEBPB | ATF4 | CEBPD | CEBPA | BATF3 | CEBPG | DDIT3 | DBP | NFIL3 | BATF | 82.7 |
| 10 | Monocytes | JMJD1C | 79.98 |
| 10 | Monocytes | NCOR1 | 79.72 |
| 11 | RestDCs | SPI1 | IRF8 | IRF1 | STAT3 | BCL11A | IRF2 | STAT5A | STAT1 | IRF3 | STAT2 | BCL6 | SPIB | IRF4 | STAT5B | STAT4 | PRDM1 | 85.59 |
| 11 | RestDCs | SMAD1 | 75.17 |
| 11 | RestDCs | BRD4 | 68.67 |
| 11 | RestDCs | KMT2A | 68.63 |
| 11 | RestDCs | JMJD1C | 58.7 |
| 11 | RestDCs | CBFB | RUNX1 | RUNX2 | RUNX3 | 58.69 |
| 11 | RestDCs | CEBPD | CEBPB | ATF4 | CEBPA | BATF3 | CEBPG | DBP | DDIT3 | BATF | NFIL3 | CEBPE | 55.11 |
| 11 | RestDCs | CDK8 | 52.52 |
| 11 | RestDCs | LYL1 | TCF4 | ASCL2 | TCF12 | TFAP4 | ZBTB18 | ZEB1 | SNAI1 | TCF3 | GATA3 | MESP1 | NEUROD2 | 51.35 |
| 11 | RestDCs | TP53 | 51.01 |
| 12 | Monocytes | SPI1 | IRF1 | STAT1 | STAT3 | IRF8 | IRF2 | IRF3 | STAT2 | BCL6 | STAT5A | STAT4 | STAT5B | PRDM1 | BCL11A | SPIB | IRF4 | 48.53 |
| 12 | Monocytes | CEBPB | CEBPD | ATF4 | CEBPG | CEBPA | DBP | BATF | DDIT3 | NFIL3 | CEBPE | BATF3 | 43.59 |
| 12 | Monocytes | KMT2A | 38.53 |
| 12 | Monocytes | JMJD1C | 37.19 |
| 12 | Monocytes | BRD4 | 34.37 |
| 12 | Monocytes | CBFB | RUNX3 | RUNX1 | RUNX2 | 33.67 |
| 12 | Monocytes | ELF1 | ETS1 | FLI1 | ELF2 | ELK4 | ETV6 | ETS2 | GABPA | ELK3 | NFAT5 | ELK1 | ETV5 | ETV2 | ETV7 | ELF3 | 28.43 |
| 12 | Monocytes | LYL1 | GATA3 | ASCL2 | TCF12 | ZBTB18 | ZEB1 | TCF3 | TCF4 | TFAP4 | MESP1 | SNAI1 | NEUROD2 | 27.91 |
| 12 | Monocytes | MED12 | 26.01 |
| 12 | Monocytes | MAX | ATF3 | USF2 | MLX | MYC | BHLHE40 | MXI1 | ARNTL | TFEB | TFE3 | CLOCK | HES1 | USF1 | MITF | 25.44 |
| 13 | CD8Tcells | STAT6 | 21.45 |
| 13 | CD8Tcells | IRF1 | STAT3 | IRF3 | STAT1 | STAT4 | STAT5A | IRF2 | STAT5B | STAT2 | SPI1 | IRF8 | PRDM1 | BCL6 | IRF4 | BCL11A | SPIB | 18.44 |
| 13 | CD8Tcells | MYBL1 | MYB | 17.43 |
| 13 | CD8Tcells | GATA3 | ZEB1 | TFAP4 | TCF12 | LYL1 | ZBTB18 | TCF3 | ASCL2 | TCF4 | MESP1 | NEUROD2 | 15.71 |
| 13 | CD8Tcells | TBX21 | EOMES | ZNF18 | TBX19 | 15.18 |
| 13 | CD8Tcells | CDK9 | 12.93 |
| 13 | CD8Tcells | FOXP3 | 12.7 |
| 13 | CD8Tcells | BRD4 | 11.69 |
| 13 | CD8Tcells | CREBBP | 11.5 |
| 13 | CD8Tcells | REL | NFKB2 | RELA | NFKB1 | RELB | HIVEP1 | 11.41 |
| 14 | NaiveBcells | FOXP1 | FOXO3 | FOXO1 | FOXK1 | FOXJ2 | FOXO4 | 33.16 |
| 14 | NaiveBcells | CD74 | 24.63 |
| 14 | NaiveBcells | IRF1 | IRF8 | STAT3 | SPIB | BCL11A | IRF3 | SPI1 | STAT1 | IRF2 | STAT4 | STAT5A | STAT2 | STAT5B | PRDM1 | BCL6 | IRF4 | 24.5 |
| 14 | NaiveBcells | MEF2C | MEF2A | MEF2D | 21.79 |
| 14 | NaiveBcells | CREBBP | 20.3 |
| 14 | NaiveBcells | CIITA | 19.2 |
| 14 | NaiveBcells | NCOR1 | 18.86 |
| 14 | NaiveBcells | NCOR2 | 18.55 |
| 14 | NaiveBcells | SND1 | 17.66 |
| 14 | NaiveBcells | IKZF1 | RBPJ | 15.93 |
| 15 | NaiveBcells | TCF4 | TCF3 | LYL1 | ZBTB18 | TCF12 | TFAP4 | ZEB1 | SNAI1 | ASCL2 | GATA3 | 121.21 |
| 15 | NaiveBcells | KMT2A | 93.01 |
| 15 | NaiveBcells | KLF6 | KLF4 | KLF13 | KLF3 | SP3 | ZNF148 | KLF16 | ZNF281 | SP2 | KLF9 | SP4 | EGR1 | SP1 | ZBTB17 | KLF12 | 91.45 |
| 15 | NaiveBcells | YY1 | TAF1 | YY2 | 86.18 |
| 15 | NaiveBcells | REL | RELA | NFKB1 | HIVEP1 | NFKB2 | RELB | 84.69 |
| 15 | NaiveBcells | CREBBP | 84.55 |
| 15 | NaiveBcells | TCF7 | LEF1 | 84.25 |
| 15 | NaiveBcells | USF2 | MAX | ATF3 | MLX | BHLHE40 | TFEB | MXI1 | CLOCK | TFE3 | MYC | ARNTL | MITF | 82.48 |
| 15 | NaiveBcells | IRF8 | BCL11A | SPIB | IRF4 | IRF1 | IRF3 | STAT1 | STAT2 | SPI1 | STAT3 | IRF2 | STAT5B | STAT5A | STAT4 | BCL6 | PRDM1 | 81.88 |
| 15 | NaiveBcells | TBP | 81.79 |
| 16 | MacrophagesM0 | LYL1 | TAL1 | GATA2 | ASCL2 | GATA1 | TCF4 | ZEB1 | ZBTB18 | GATA3 | TFAP4 | TCF12 | SNAI1 | TCF3 | MESP1 | 52.27 |
| 16 | MacrophagesM0 | LMO2 | 36.95 |
| 16 | MacrophagesM0 | KLF6 | KLF4 | KLF3 | KLF13 | KLF9 | SP3 | ZNF281 | EGR1 | ZNF148 | SP1 | KLF12 | KLF16 | SP2 | ZBTB17 | SP4 | EGR2 | KLF5 | 31.57 |
| 16 | MacrophagesM0 | FOS | JUNB | JUN | FOSB | JUND | NFE2 | BACH1 | NFE2L2 | FOSL2 | BACH2 | ZNF554 | 30.45 |
| 16 | MacrophagesM0 | KMT2A | 30.35 |
| 16 | MacrophagesM0 | RUNX1 | RUNX3 | CBFB | RUNX2 | 28.35 |
| 16 | MacrophagesM0 | RAD21 | 28.28 |
| 16 | MacrophagesM0 | ELF1 | FLI1 | ETS1 | ELK3 | ELF2 | ETV6 | ETS2 | ELK4 | GABPA | NFAT5 | ETV5 | ELK1 | 27.63 |
| 16 | MacrophagesM0 | SPI1 | IRF1 | STAT3 | IRF8 | STAT2 | IRF3 | BCL6 | STAT1 | IRF2 | STAT5B | BCL11A | PRDM1 | STAT5A | STAT4 | SPIB | IRF4 | 27.14 |
| 16 | MacrophagesM0 | TRIM28 | 26.51 |
| 17 | Monocytes | SPI1 | IRF1 | IRF8 | BCL11A | STAT1 | SPIB | IRF2 | IRF3 | STAT3 | STAT5B | STAT2 | BCL6 | STAT5A | STAT4 | PRDM1 | IRF4 | 34.36 |
| 17 | Monocytes | CEBPB | CEBPD | ATF4 | CEBPG | NFIL3 | CEBPA | DDIT3 | BATF | DBP | 28.11 |
| 17 | Monocytes | KMT2A | 25.95 |
| 17 | Monocytes | JMJD1C | 20.76 |
| 17 | Monocytes | LYL1 | TCF4 | TCF3 | TCF12 | ASCL2 | ZBTB18 | TFAP4 | ZEB1 | 20.38 |
| 17 | Monocytes | CBFB | RUNX3 | RUNX1 | RUNX2 | 20.17 |
| 17 | Monocytes | LMO2 | 19.06 |
| 17 | Monocytes | ELF1 | FLI1 | ETS1 | ELF2 | ETS2 | GABPA | ELK4 | ETV6 | ELK3 | NFAT5 | 16.62 |
| 17 | Monocytes | BRD4 | 15.94 |
| 17 | Monocytes | FOXP1 | FOXO3 | FOXO1 | FOXK1 | FOXO4 | FOXJ2 | 14.01 |
| 18 | ActMast | RUNX1 | CBFB | RUNX3 | RUNX2 | 44.45 |
| 18 | ActMast | LYL1 | GATA2 | TCF4 | GATA1 | TFAP4 | TCF12 | TAL1 | TCF3 | GATA3 | MESP1 | ASCL2 | ZEB1 | 41.75 |
| 18 | ActMast | FOS | JUN | JUNB | FOSB | JUND | NFE2 | NFE2L2 | BACH1 | FOSL2 | ZNF554 | BACH2 | 38.9 |
| 18 | ActMast | KLF6 | KLF4 | EGR1 | KLF1 | ZNF148 | ZNF281 | KLF3 | KLF13 | KLF9 | SP3 | SP2 | ZBTB17 | KLF12 | SP1 | KLF16 | 37.44 |
| 18 | ActMast | ELF1 | ETS2 | ETV6 | FLI1 | ERG | ELF2 | ELK3 | NFAT5 | ELK4 | ETS1 | ETV2 | GABPA | ETV5 | ELK1 | 32.37 |
| 18 | ActMast | IRF1 | SPI1 | STAT3 | STAT5A | BCL11A | IRF2 | STAT2 | STAT5B | IRF3 | IRF8 | STAT4 | BCL6 | STAT1 | SPIB | 32.03 |
| 18 | ActMast | TBP | 28.97 |
| 18 | ActMast | BPTF | 26.66 |
| 18 | ActMast | KMT2A | 25.21 |
| 18 | ActMast | RAD21 | 25 |
| 2 | RestMemCD4Tcells | STAT6 | 26.89 |
| 2 | RestMemCD4Tcells | MYBL1 | MYB | 25.97 |
| 2 | RestMemCD4Tcells | REL | NFKB2 | RELA | NFKB1 | RELB | HIVEP1 | 22.44 |
| 2 | RestMemCD4Tcells | CDK9 | 22.2 |
| 2 | RestMemCD4Tcells | BRD4 | 21.95 |
| 2 | RestMemCD4Tcells | KMT2A | 21.53 |
| 2 | RestMemCD4Tcells | ETS1 | ELF1 | FLI1 | ELF2 | ELK4 | ELK3 | NFAT5 | ELK1 | ETV6 | GABPA | ETS2 | ETV7 | ETV2 | ELF3 | 21.05 |
| 2 | RestMemCD4Tcells | GATA3 | ZEB1 | LYL1 | TFAP4 | TCF12 | TCF3 | ZBTB18 | TCF4 | ASCL2 | MESP1 | SNAI1 | NEUROD2 | 32.37 |
| 2 | RestMemCD4Tcells | IRF1 | STAT3 | IRF3 | STAT1 | IRF2 | STAT5A | STAT4 | STAT5B | PRDM1 | STAT2 | SPI1 | IRF8 | BCL6 | IRF4 | SPIB | BCL11A | 28.81 |
| 2 | RestMemCD4Tcells | TBX21 | ZNF18 | EOMES | TBX19 | 36.34 |
| 3 | NaiveCD4Tcells | KAT2B | 28.43 |
| 3 | NaiveCD4Tcells | MYB | MYBL1 | 30.59 |
| 3 | NaiveCD4Tcells | BRD4 | 29.42 |
| 3 | NaiveCD4Tcells | EZH2 | 26.27 |
| 3 | NaiveCD4Tcells | HDAC6 | 25.72 |
| 3 | NaiveCD4Tcells | CREBBP | 25.39 |
| 3 | NaiveCD4Tcells | RUNX3 | RUNX2 | CBFB | RUNX1 | 24.98 |
| 3 | NaiveCD4Tcells | STAT3 | IRF1 | STAT1 | IRF3 | IRF2 | STAT4 | STAT5A | STAT5B | STAT2 | SPI1 | IRF8 | BCL11A | BCL6 | SPIB | IRF4 | PRDM1 | 26.51 |
| 3 | NaiveCD4Tcells | MYC | MAX | USF2 | MLX | MXI1 | CLOCK | ARNTL | USF1 | ATF3 | TFE3 | BHLHE40 | TFEB | HES1 | MITF | 27.89 |
| 3 | NaiveCD4Tcells | CDK9 | 27.33 |
| 4 | CD8Tcells | EOMES | TBX21 | ZNF18 | TBX19 | 56.84 |
| 4 | CD8Tcells | GATA3 | ASCL2 | ZEB1 | TCF12 | TFAP4 | LYL1 | TCF3 | MESP1 | ZBTB18 | TCF4 | SNAI1 | NEUROD2 | 58.72 |
| 4 | CD8Tcells | CDK9 | 35.18 |
| 4 | CD8Tcells | IRF1 | STAT3 | IRF3 | STAT1 | IRF2 | STAT4 | STAT5A | STAT5B | STAT2 | PRDM1 | SPI1 | IRF8 | BCL6 | IRF4 | SPIB | BCL11A | 44.41 |
| 4 | CD8Tcells | MYBL1 | MYB | 41.64 |
| 4 | CD8Tcells | KMT2A | 41.1 |
| 4 | CD8Tcells | REL | RELA | NFKB2 | NFKB1 | RELB | HIVEP1 | 30.22 |
| 4 | CD8Tcells | ETS1 | ELF1 | FLI1 | ELF2 | ELK4 | NFAT5 | ELK3 | GABPA | ELK1 | ETS2 | ETV6 | ETV2 | ETV7 | ETV5 | ELF3 | 33.75 |
| 4 | CD8Tcells | RUNX3 | CBFB | RUNX1 | RUNX2 | 31.95 |
| 4 | CD8Tcells | IKZF1 | RBPJ | 30.27 |
| 5 | NaiveBcells | IRF8 | BCL11A | SPIB | IRF1 | SPI1 | STAT3 | IRF2 | IRF3 | STAT2 | STAT1 | STAT5A | IRF4 | STAT5B | BCL6 | STAT4 | PRDM1 | 82.03 |
| 5 | NaiveBcells | SND1 | 55.35 |
| 5 | NaiveBcells | MEF2C | MEF2A | MEF2D | 72.02 |
| 5 | NaiveBcells | CREBBP | 68.48 |
| 5 | NaiveBcells | CD74 | 63.56 |
| 5 | NaiveBcells | IKZF1 | RBPJ | ARNT2 | 45.29 |
| 5 | NaiveBcells | NCOR1 | 54.56 |
| 5 | NaiveBcells | NCOR2 | 53.57 |
| 5 | NaiveBcells | KMT2A | 48.68 |
| 5 | NaiveBcells | FOXP1 | FOXO1 | FOXK1 | FOXO3 | FOXJ2 | FOXO4 | FOXM1 | 97.67 |
| 6 | MemoryBcells | MEF2C | MEF2A | MEF2D | 62.45 |
| 6 | MemoryBcells | CREBBP | 58.1 |
| 6 | MemoryBcells | NCOR2 | 51.01 |
| 6 | MemoryBcells | SND1 | 50.33 |
| 6 | MemoryBcells | EBF1 | 41.54 |
| 6 | MemoryBcells | KMT2A | 41.46 |
| 6 | MemoryBcells | NCOR1 | 40.39 |
| 6 | MemoryBcells | IRF8 | SPIB | BCL11A | IRF1 | SPI1 | STAT3 | IRF3 | IRF2 | STAT1 | STAT2 | STAT5A | STAT5B | IRF4 | BCL6 | STAT4 | PRDM1 | 66.74 |
| 6 | MemoryBcells | CD74 | 63.97 |
| 6 | MemoryBcells | FOXP1 | FOXO3 | FOXK1 | FOXO1 | FOXO4 | FOXJ2 | FOXM1 | 82.12 |
| 7 | ActNK | IRF1 | IRF3 | STAT3 | STAT4 | IRF2 | STAT1 | PRDM1 | STAT5A | STAT2 | STAT5B | IRF8 | SPI1 | SPIB | BCL6 | BCL11A | IRF4 | 42.21 |
| 7 | ActNK | GATA3 | ASCL2 | ZEB1 | TCF12 | TFAP4 | TCF3 | LYL1 | ZBTB18 | MESP1 | SNAI1 | TCF4 | NEUROD2 | 56.26 |
| 7 | ActNK | EOMES | TBX21 | ZNF18 | TBX19 | 54.83 |
| 7 | ActNK | MAF | MAFF | MAFG | MAFK | MAFB | NRL | 27.47 |
| 7 | ActNK | ETS1 | ELF1 | FLI1 | ELF2 | ELK4 | NFAT5 | ELK3 | GABPA | ELK1 | ETV6 | ETS2 | ETV7 | ETV2 | ELF3 | ETV5 | 26.9 |
| 7 | ActNK | RUNX3 | CBFB | RUNX1 | RUNX2 | 26.85 |
| 7 | ActNK | BRD4 | 25.23 |
| 7 | ActNK | CDK9 | 31.41 |
| 7 | ActNK | KMT2A | 41.86 |
| 7 | ActNK | MYBL1 | MYB | 37.33 |
| 8 | ActNK | TBX21 | EOMES | ZNF18 | TBX19 | 140.01 |
| 8 | ActNK | GATA3 | ASCL2 | ZEB1 | TCF3 | TCF4 | TCF12 | MESP1 | TFAP4 | LYL1 | ZBTB18 | SNAI1 | GATA2 | NEUROD2 | 144.65 |
| 8 | ActNK | ETS1 | ELF1 | FLI1 | ELF2 | NFAT5 | ELK4 | GABPA | ELK1 | ETV6 | ELK3 | ETS2 | ETV2 | ETV7 | ETV5 | 93.43 |
| 8 | ActNK | KMT2A | 126.02 |
| 8 | ActNK | MYBL1 | MYB | 107.39 |
| 8 | ActNK | IRF1 | IRF3 | STAT3 | STAT4 | IRF8 | IRF2 | STAT1 | PRDM1 | STAT2 | STAT5A | STAT5B | SPI1 | BCL6 | SPIB | BCL11A | IRF4 | 96.92 |
| 8 | ActNK | MAFF | MAF | MAFG | MAFB | NRL | MAFK | 83.74 |
| 8 | ActNK | CDK9 | 90.59 |
| 8 | ActNK | RUNX3 | CBFB | RUNX1 | RUNX2 | 86.93 |
| 8 | ActNK | NR4A2 | RXRB | THRA | RARA | NR4A1 | ESRRA | NR2C1 | RARG | RXRA | PPARD | PPARA | NR1H3 | NR4A3 | NR2F6 | ESR1 | PPARG | 83.93 |
| 9 | NaiveCD4Tcells | EZH2 | 27.07 |
| 9 | NaiveCD4Tcells | CREBBP | 24.29 |
| 9 | NaiveCD4Tcells | MYB | MYBL1 | 27 |
| 9 | NaiveCD4Tcells | WDR5 | 25.39 |
| 9 | NaiveCD4Tcells | BRD4 | 25.34 |
| 9 | NaiveCD4Tcells | MAFK | MAFG | MAF | MAFB | MAFF | NRL | 20.74 |
| 9 | NaiveCD4Tcells | IRF1 | STAT3 | IRF3 | STAT1 | IRF2 | STAT4 | STAT5A | STAT5B | STAT2 | SPI1 | IRF8 | SPIB | BCL6 | BCL11A | IRF4 | PRDM1 | 24.19 |
| 9 | NaiveCD4Tcells | ETS1 | ELF1 | FLI1 | ELK4 | ELF2 | ELK1 | NFAT5 | ELK3 | GABPA | ETS2 | ETV6 | ETV2 | ELF3 | ETV7 | 23.41 |
| 9 | NaiveCD4Tcells | HDAC6 | 20.85 |
| 9 | NaiveCD4Tcells | CDK9 | 28.14 |

Copyright @2019 Liu lab
