# Supplementary material for: Integrative analyses of single-cell transcriptome and regulome using MAESTRO
Source: Genome Biol. 2020 Aug 7;21:198. doi: 10.1186/s13059-020-02116-x (PMC7412809; doi:10.1186/s13059-020-02116-x)
Supplement: Supplementary file 9 — Additional file 9. HTML output for the scATAC-seq analysis on the human PBMC sample (10k cells) from different donors using MAESTRO. [file 13059_2020_2116_MOESM9_ESM.html]

MAESTRO


MAESTRO

- scRNA-seq module
- scATAC-seq module
- Integration module

- Sample Information
- Quality Control
  - Bulk level
  - Single-cell level
- Cell Clustering
- Annotation
  - Celltype annotation
  - Regulator annotation
- Gene Track

# Sample Information

|  |  |
| --- | --- |
| Sample ID | 10X\_PBMC\_10k |
| FASTQ Path | /home1/wangchenfei/Project/SingleCell/scATAC/Analysis/10X\_PBMC\_10k\_MAESTRO/atac\_v1\_pbmc\_10k\_fastqs |
| Species | GRCh38 |
| Platform | 10x-genomics |

# Quality Control

## Bulk level

### Mappability

Read mappability, duplicated reads percentage, fraction of reads mapped to mitochondrial genes, peak regions or promoter regions.

- The **percentage of duplicate reads** shows whether the library is over-amplified due to limited starting material. For human or mouse, if the percentage of duplicate reads is more than 50%, there may be something wrong during the library preparation.
- The **percentage of uniquely mapped reads** is usually more than 50%. If the percentage is less than 50%, it might be due to inadequate sequencing depth or excessive PCR amplification.
- **Reads derived from mitochondrial DNA** represent noise in ATAC-seq data, and should be discarded in the downstream analysis. The mitochondrial read fraction shows a large degree of variation across different species and cell types.
- The **fraction of reads in peak or promoter regions** is used to evaluate the signal-to-noise ratio of ATAC-seq data quality. At the bulk level, if the values are less than 2%, it might indicate that the ATAC-seq assay does not capture strong signals.

### Fragment size distribution

Fragment size distribution for 1% reads sampled from scATAC-seq. Only fragments with insert size less than 1,000 are considered. Y-axis represents the number of fragments. The fragment size distribution should show a periodicity of approximately 200bp due to nucleosome protection of the chromatin to transposase cutting.

## Single-cell level

### Cell filtering based on valid fragments and FRIP

Cell filtering plot of scATAC-seq. The x-axis represents the number of unique reads present in each cell, and the y-axis represents the fraction of reads in promoter regions (defined as 2kb up/downstream of TSS).

# Cell Clustering

### Cell clustering based on peaks

UMAP visualization of the clustering result. Colors represent different clusters with the cluster ID labeled.

# Annotation

## Celltype annotation

### Celltype annotation based on RP and DE genes

UMAP visualization of annotated clusters. Colors represent different cell types. The cell type information for each cluster is annotated using the regulatory potential of marker genes.

### Celltype annotation based on bulk chromatin accessibility data

UMAP visualization of cells. Colors represent different biological sources. The biological source information for each cluster is inferred based on cluster-specific peaks.

## Regulator annotation

### Cluster-specific regulator identified by GIGGLE

Driver transcription regulators identified based on cluster-specific peaks. By default, the regulators are ranked by the enrichment score in each cluster.

| Cluster | Celltype Annotation | Transcription Factor | log10(Giggle score) |
| --- | --- | --- | --- |
| 0 | Monocytes | IRF1 | STAT3 | SPI1 | IRF2 | BCL6 | STAT2 | BCL11A | STAT1 | IRF3 | STAT5A | IRF8 | SPIB | STAT5B | IRF4 | PRDM1 | STAT4 | 3.28 |
| 0 | Monocytes | ZNF18 | TBX21 | EOMES | TBR1 | TBX3 | T | TBX19 | TBX5 | 3.28 |
| 0 | Monocytes | RARA | NR4A2 | NR4A1 | ESRRA | NR4A3 | RXRA | RARG | PPARD | VDR | RXRB | PPARA | THRA | NR1H3 | PPARG | NR2F6 | THRB | NR2C1 | ESR2 | ESR1 | NR2F1 | NR1I3 | NR1I2 | NR5A2 | NR2F2 | RXRG | HNF4A | HNF4G | ESRRB | NR1H4 | NR5A1 | RARB | 3.27 |
| 0 | Monocytes | KMT2A | 3.22 |
| 0 | Monocytes | SMAD1 | 3.22 |
| 0 | Monocytes | BRD4 | 3.22 |
| 0 | Monocytes | GLI1 | GLI3 | GLI2 | 3.22 |
| 0 | Monocytes | DBP | NFIL3 | CEBPA | DDIT3 | ATF4 | CEBPG | CEBPB | CEBPD | BATF3 | CEBPE | BATF | HLF | 3.21 |
| 0 | Monocytes | PML | 3.21 |
| 0 | Monocytes | FOS | JUNB | FOSB | JUND | FOSL2 | NFE2L2 | JUN | BACH1 | FOSL1 | NFE2 | BACH2 | ZNF554 | 3.21 |
| 1 | NaiveCD4Tcells | TET2 | 3.4 |
| 1 | NaiveCD4Tcells | ZBTB18 | GATA3 | TCF3 | TFAP4 | ZEB1 | SNAI1 | TCF12 | LYL1 | NEUROG2 | GATA2 | TCF4 | MESP1 | BHLHA15 | FIGLA | ASCL2 | SNAI2 | NEUROD2 | OLIG2 | GATA6 | TAL1 | GATA5 | GATA4 | ASCL1 | MYOG | NEUROD1 | MYOD1 | GATA1 | PTF1A | TWIST1 | 3.32 |
| 1 | NaiveCD4Tcells | ASXL1 | 3.31 |
| 1 | NaiveCD4Tcells | POLR2A | 3.3 |
| 1 | NaiveCD4Tcells | ZNF18 | EOMES | TBX21 | TBR1 | TBX3 | TBX19 | T | TBX5 | 3.28 |
| 1 | NaiveCD4Tcells | KDM4A | 3.27 |
| 1 | NaiveCD4Tcells | BRD4 | 3.27 |
| 1 | NaiveCD4Tcells | PHF8 | 3.26 |
| 1 | NaiveCD4Tcells | NRL | MAFK | MAFF | MAFG | MAF | MAFB | 3.26 |
| 1 | NaiveCD4Tcells | RUNX3 | RUNX1 | CBFB | FOXH1 | RUNX2 | 3.26 |
| 10 | Monocytes | NR4A1 | RARA | NR4A2 | ESRRA | NR4A3 | RXRA | VDR | PPARD | RARG | RXRB | THRA | PPARA | NR1H3 | NR2F6 | PPARG | THRB | NR2C1 | ESR2 | NR2F2 | ESR1 | NR1I3 | NR1I2 | RXRG | NR5A2 | NR2F1 | HNF4A | HNF4G | ESRRB | RARB | NR1H4 | NR5A1 | 3.22 |
| 10 | Monocytes | IRF1 | STAT3 | SPI1 | IRF2 | BCL6 | BCL11A | STAT1 | STAT2 | STAT5A | IRF3 | SPIB | IRF8 | STAT5B | IRF4 | PRDM1 | STAT4 | 3.2 |
| 10 | Monocytes | ZNF18 | TBX21 | EOMES | TBR1 | T | TBX19 | TBX3 | TBX5 | 3.17 |
| 10 | Monocytes | PML | 3.13 |
| 10 | Monocytes | BRD4 | 3.13 |
| 10 | Monocytes | LDB1 | 3.12 |
| 10 | Monocytes | GLI1 | GLI3 | GLI2 | 3.12 |
| 10 | Monocytes | DBP | ATF4 | DDIT3 | CEBPA | NFIL3 | BATF3 | CEBPG | CEBPD | CEBPB | BATF | CEBPE | HLF | 3.11 |
| 10 | Monocytes | POLR2A | 3.11 |
| 10 | Monocytes | KMT2A | 3.1 |
| 11 | MemoryBcells | CD74 | 3.3 |
| 11 | MemoryBcells | FOXO3 | FOXO1 | FOXJ2 | FOXA3 | FOXO6 | FOXP1 | FOXM1 | FOXO4 | FOXK1 | FOXD1 | FOXC1 | FOXP2 | FOXD3 | FOXF1 | FOXF2 | FOXQ1 | FOXA1 | FOXA2 | 3.27 |
| 11 | MemoryBcells | IRF1 | STAT3 | IRF2 | IRF3 | BCL11A | IRF8 | BCL6 | STAT2 | IRF4 | SPI1 | PRDM1 | STAT1 | STAT5A | STAT4 | STAT5B | SPIB | 3.27 |
| 11 | MemoryBcells | NCOR2 | 3.27 |
| 11 | MemoryBcells | EP300 | 3.27 |
| 11 | MemoryBcells | CREBBP | 3.26 |
| 11 | MemoryBcells | MEF2D | MEF2A | MEF2C | MEF2B | 3.24 |
| 11 | MemoryBcells | PAX5 | ZNF121 | 3.23 |
| 11 | MemoryBcells | SND1 | 3.23 |
| 11 | MemoryBcells | NCOR1 | 3.22 |
| 12 | Monocytes | IRF1 | SPI1 | STAT3 | STAT2 | BCL11A | IRF2 | BCL6 | IRF3 | IRF4 | STAT1 | STAT4 | SPIB | STAT5A | IRF8 | PRDM1 | STAT5B | 3.23 |
| 12 | Monocytes | GLI1 | GLI3 | GLI2 | 3.2 |
| 12 | Monocytes | ZBTB18 | LYL1 | ASCL2 | SNAI1 | TCF3 | TFAP4 | TCF12 | TCF4 | ZEB1 | MYOG | FIGLA | GATA3 | BHLHA15 | MESP1 | GATA2 | NEUROG2 | TAL1 | OLIG2 | SNAI2 | NEUROD2 | GATA6 | GATA5 | NEUROD1 | MYOD1 | TWIST1 | GATA4 | ASCL1 | PTF1A | ATOH1 | GATA1 | 3.19 |
| 12 | Monocytes | JMJD1C | 3.18 |
| 12 | Monocytes | IKZF2 | 3.17 |
| 12 | Monocytes | BRD4 | 3.17 |
| 12 | Monocytes | RUNX1 | RUNX3 | CBFB | RUNX2 | FOXH1 | 3.16 |
| 12 | Monocytes | SMARCA4 | 3.16 |
| 12 | Monocytes | SMARCC1 | 3.16 |
| 12 | Monocytes | ZNF366 | 3.16 |
| 13 | CD8Tcells | ZFX | ZNF770 | HAND1 | 3.54 |
| 13 | CD8Tcells | POLR2A | 3.51 |
| 13 | CD8Tcells | MYC | MXI1 | BHLHE40 | USF1 | TFEB | USF2 | HES7 | MAX | ATF3 | HES1 | HEY1 | HES5 | CLOCK | ARNTL | TFE3 | MLX | CREB3L1 | MYCN | MITF | 3.48 |
| 13 | CD8Tcells | BRD2 | 3.48 |
| 13 | CD8Tcells | NELFE | 3.45 |
| 13 | CD8Tcells | IRAK1 | 3.45 |
| 13 | CD8Tcells | GMEB2 | EPAS1 | HIF1A | ARNT | AHR | E4F1 | 3.42 |
| 13 | CD8Tcells | SUPT5H | 3.42 |
| 13 | CD8Tcells | CREBBP | 3.4 |
| 13 | CD8Tcells | BRD4 | 3.4 |
| 14 | MemoryBcells | KMT2D | 3.39 |
| 14 | MemoryBcells | CHD4 | 3.39 |
| 14 | MemoryBcells | DNMT3A | 3.38 |
| 14 | MemoryBcells | SOX4 | POU3F1 | SOX13 | POU5F1 | SOX5 | SOX2 | SOX15 | SOX10 | NANOG | SOX17 | SRY | 3.38 |
| 14 | MemoryBcells | MLLT1 | 3.37 |
| 14 | MemoryBcells | POU3F2 | 3.37 |
| 14 | MemoryBcells | POLR2A | 3.36 |
| 14 | MemoryBcells | PSMD1 | 3.36 |
| 14 | MemoryBcells | SRC | 3.33 |
| 14 | MemoryBcells | SETX | 3.31 |
| 15 | ActNK | TP53 | TP73 | 3.21 |
| 15 | ActNK | TBX21 | ZNF18 | EOMES | TBR1 | TBX19 | T | 3.2 |
| 15 | ActNK | GATA3 | ZBTB18 | TCF3 | TFAP4 | ZEB1 | TCF12 | LYL1 | TCF4 | SNAI1 | ASCL2 | MESP1 | SNAI2 | NEUROG2 | GATA6 | BHLHA15 | GATA2 | NEUROD2 | TAL1 | FIGLA | OLIG2 | MYOG | GATA5 | GATA4 | ASCL1 | GATA1 | NEUROD1 | PTF1A | 3.19 |
| 15 | ActNK | RUNX3 | RUNX1 | RUNX2 | CBFB | FOXH1 | 3.18 |
| 15 | ActNK | EP300 | 3.18 |
| 15 | ActNK | IRF1 | IRF2 | STAT3 | BCL6 | STAT4 | IRF3 | STAT5A | STAT1 | STAT2 | PRDM1 | STAT5B | BCL11A | IRF8 | SPI1 | IRF4 | SPIB | 3.16 |
| 15 | ActNK | MYBL1 | MYB | 3.14 |
| 15 | ActNK | ELF2 | FLI1 | GABPA | ETV6 | ETV5 | ELK1 | ETS1 | NFAT5 | ETV2 | ELF1 | ETS2 | ELK3 | ELK4 | ETV7 | ETV4 | EHF | ETV1 | FEV | ERG | ELF5 | ELF3 | 3.13 |
| 15 | ActNK | ZBTB16 | 3.13 |
| 15 | ActNK | KMT2A | 3.11 |
| 16 | NaiveBcells | ZBTB18 | TFAP4 | TCF3 | LYL1 | ZEB1 | SNAI1 | BHLHA15 | TCF4 | TCF12 | TAL1 | NEUROG2 | FIGLA | GATA3 | ASCL2 | MYOG | SNAI2 | GATA2 | OLIG2 | NEUROD1 | MESP1 | NEUROD2 | GATA5 | MYOD1 | TWIST1 | GATA4 | PTF1A | GATA6 | GATA1 | ASCL1 | 3.27 |
| 16 | NaiveBcells | EP300 | 3.22 |
| 16 | NaiveBcells | TCF7 | TCF7L2 | LEF1 | TCF7L1 | 3.2 |
| 16 | NaiveBcells | CD74 | 3.2 |
| 16 | NaiveBcells | RUNX1 | RUNX2 | CBFB | RUNX3 | FOXH1 | 3.19 |
| 16 | NaiveBcells | FOXO3 | FOXO6 | FOXJ2 | FOXP1 | FOXA3 | FOXO1 | FOXK1 | FOXO4 | FOXA1 | FOXM1 | FOXD1 | FOXC1 | FOXF2 | FOXP2 | FOXD3 | FOXF1 | FOXQ1 | FOXA2 | 3.18 |
| 16 | NaiveBcells | STAT3 | BCL6 | IRF4 | IRF1 | IRF2 | BCL11A | STAT4 | STAT2 | IRF3 | STAT5A | IRF8 | STAT1 | PRDM1 | SPI1 | STAT5B | SPIB | 3.17 |
| 16 | NaiveBcells | PBX3 | PKNOX1 | PBX1 | MEIS1 | HOXB7 | HOXA9 | HOXB8 | PBX2 | HOXC8 | HOXA1 | HOXA10 | HOXB6 | HOXA5 | HOXB1 | PDX1 | 3.16 |
| 16 | NaiveBcells | RBPJ | IKZF1 | ARNT2 | 3.15 |
| 16 | NaiveBcells | MYB | MYBL1 | 3.15 |
| 2 | Monocytes | ZNF18 | TBX21 | EOMES | TBR1 | T | TBX3 | TBX19 | TBX5 | 3.25 |
| 2 | Monocytes | RARA | NR4A2 | NR4A1 | ESRRA | NR4A3 | PPARD | RXRA | RARG | VDR | PPARG | RXRB | PPARA | NR1H3 | THRA | NR2F6 | THRB | NR2C1 | ESR2 | ESR1 | NR1I3 | NR1I2 | NR5A2 | NR2F1 | NR2F2 | RXRG | HNF4A | NR1H4 | RARB | NR5A1 | HNF4G | ESRRB | 3.25 |
| 2 | Monocytes | IRF1 | SPI1 | STAT3 | BCL6 | IRF2 | STAT2 | BCL11A | STAT1 | IRF3 | STAT5A | IRF8 | STAT5B | SPIB | PRDM1 | IRF4 | STAT4 | 3.25 |
| 2 | Monocytes | NFIL3 | CEBPA | DDIT3 | DBP | ATF4 | CEBPG | CEBPB | BATF3 | CEBPD | CEBPE | BATF | HLF | 3.22 |
| 2 | Monocytes | SMAD1 | 3.21 |
| 2 | Monocytes | ELF2 | FLI1 | ETS2 | ETV5 | NFAT5 | ETV6 | ELK1 | ELF1 | ETV2 | GABPA | ETV7 | ETS1 | ELK3 | ELK4 | ETV4 | ETV1 | ERG | EHF | FEV | ELF5 | ELF3 | 3.2 |
| 2 | Monocytes | EP300 | 3.2 |
| 2 | Monocytes | SKI | 3.2 |
| 2 | Monocytes | GMEB2 | HIF1A | ARNT | EPAS1 | E4F1 | AHR | 3.19 |
| 2 | Monocytes | PML | 3.19 |
| 3 | NaiveCD4Tcells | POLR2A | 3.43 |
| 3 | NaiveCD4Tcells | PHF8 | 3.4 |
| 3 | NaiveCD4Tcells | RUNX3 | RUNX1 | CBFB | RUNX2 | FOXH1 | 3.36 |
| 3 | NaiveCD4Tcells | ZNF18 | TBX21 | EOMES | TBR1 | TBX19 | TBX3 | T | TBX5 | 3.36 |
| 3 | NaiveCD4Tcells | KDM4A | 3.34 |
| 3 | NaiveCD4Tcells | BRD4 | 3.33 |
| 3 | NaiveCD4Tcells | MYC | BHLHE40 | MAX | MXI1 | USF1 | TFEB | HES7 | ARNTL | USF2 | CLOCK | ATF3 | TFE3 | HEY1 | HES5 | HES1 | MLX | CREB3L1 | MITF | MYCN | 3.33 |
| 3 | NaiveCD4Tcells | NFKB2 | HIVEP1 | RELA | REL | RELB | NFKB1 | 3.33 |
| 3 | NaiveCD4Tcells | KLF13 | EGR1 | KLF6 | KLF16 | SP1 | KLF9 | SP3 | KLF3 | EGR2 | SP4 | SP2 | ZBTB17 | ZNF281 | ZNF148 | KLF1 | EGR4 | KLF5 | TBX1 | KLF4 | KLF12 | KLF14 | TBX15 | 3.32 |
| 3 | NaiveCD4Tcells | NELFA | 3.31 |
| 4 | CD8Tcells | POLR2A | 3.28 |
| 4 | CD8Tcells | BRD4 | 3.24 |
| 4 | CD8Tcells | CREBBP | 3.22 |
| 4 | CD8Tcells | EP300 | 3.21 |
| 4 | CD8Tcells | ZNF18 | EOMES | TBX21 | TBR1 | TBX3 | TBX19 | T | TBX5 | 3.21 |
| 4 | CD8Tcells | CDK9 | 3.2 |
| 4 | CD8Tcells | MYB | MYBL1 | 3.19 |
| 4 | CD8Tcells | MAFK | NRL | MAFF | MAFG | MAF | MAFB | 3.19 |
| 4 | CD8Tcells | RAG2 | 3.18 |
| 4 | CD8Tcells | RUNX3 | RUNX1 | CBFB | RUNX2 | FOXH1 | 3.18 |
| 5 | CD8Tcells | TBX21 | ZNF18 | EOMES | TBR1 | TBX19 | T | TBX5 | TBX3 | 3.26 |
| 5 | CD8Tcells | POLR2A | 3.23 |
| 5 | CD8Tcells | GATA3 | ZBTB18 | TCF3 | ZEB1 | TFAP4 | LYL1 | ASCL2 | TCF12 | SNAI1 | GATA6 | NEUROG2 | MESP1 | SNAI2 | GATA2 | NEUROD2 | BHLHA15 | TCF4 | OLIG2 | FIGLA | TAL1 | GATA4 | GATA5 | MYOG | GATA1 | NEUROD1 | MYOD1 | PTF1A | ASCL1 | 3.2 |
| 5 | CD8Tcells | CREBBP | 3.2 |
| 5 | CD8Tcells | HDAC3 | 3.2 |
| 5 | CD8Tcells | EP300 | 3.19 |
| 5 | CD8Tcells | TP53 | TP63 | TP73 | 3.19 |
| 5 | CD8Tcells | HIVEP1 | NFKB2 | RELA | REL | RELB | NFKB1 | 3.18 |
| 5 | CD8Tcells | RUNX3 | RUNX1 | RUNX2 | CBFB | FOXH1 | 3.18 |
| 5 | CD8Tcells | CDK9 | 3.18 |
| 6 | ActNK | POLR2A | 3.4 |
| 6 | ActNK | TET2 | 3.33 |
| 6 | ActNK | GATA3 | TCF3 | ZBTB18 | TFAP4 | ZEB1 | TCF12 | LYL1 | SNAI1 | ASCL2 | NEUROG2 | GATA6 | MESP1 | SNAI2 | NEUROD2 | GATA2 | TCF4 | OLIG2 | FIGLA | BHLHA15 | TAL1 | ASCL1 | GATA5 | NEUROD1 | MYOG | GATA4 | MYOD1 | GATA1 | PTF1A | TWIST1 | 3.31 |
| 6 | ActNK | TBX21 | EOMES | ZNF18 | TBR1 | TBX19 | TBX3 | T | TBX5 | 3.31 |
| 6 | ActNK | PHF8 | 3.3 |
| 6 | ActNK | BRD4 | 3.3 |
| 6 | ActNK | HDAC1 | 3.3 |
| 6 | ActNK | HIVEP1 | NFKB2 | RELA | REL | RELB | NFKB1 | 3.3 |
| 6 | ActNK | MED1 | 3.29 |
| 6 | ActNK | BPTF | 3.29 |
| 7 | ActNK | TBX21 | ZNF18 | EOMES | TBR1 | TBX19 | TBX3 | T | TBX5 | 3.25 |
| 7 | ActNK | GATA3 | ZBTB18 | TCF3 | TFAP4 | ASCL2 | TCF12 | LYL1 | ZEB1 | SNAI1 | TCF4 | MESP1 | GATA6 | OLIG2 | FIGLA | NEUROG2 | GATA2 | NEUROD2 | SNAI2 | BHLHA15 | TAL1 | GATA5 | MYOG | GATA4 | NEUROD1 | GATA1 | MYOD1 | ASCL1 | PTF1A | TWIST1 | 3.23 |
| 7 | ActNK | TP53 | TP73 | TP63 | 3.2 |
| 7 | ActNK | KMT2A | 3.17 |
| 7 | ActNK | RUNX3 | RUNX1 | CBFB | FOXH1 | RUNX2 | 3.16 |
| 7 | ActNK | IRF1 | STAT3 | IRF3 | STAT4 | IRF2 | BCL6 | STAT2 | PRDM1 | STAT1 | STAT5A | STAT5B | IRF8 | IRF4 | SPI1 | BCL11A | SPIB | 3.16 |
| 7 | ActNK | EP300 | 3.16 |
| 7 | ActNK | FOXP3 | 3.14 |
| 7 | ActNK | MYBL1 | MYB | 3.14 |
| 7 | ActNK | ETV5 | FLI1 | ELF2 | ETV2 | ETS1 | GABPA | NFAT5 | ELF1 | ELK1 | ETV6 | ELK3 | ELK4 | ETS2 | ETV4 | ETV7 | ETV1 | EHF | FEV | ELF3 | ERG | ELF5 | 3.13 |
| 8 | NaiveBcells | CD74 | 3.3 |
| 8 | NaiveBcells | FOXO3 | FOXP1 | FOXO1 | FOXO6 | FOXJ2 | FOXA3 | FOXO4 | FOXM1 | FOXK1 | FOXD1 | FOXP2 | FOXC1 | FOXF2 | FOXD3 | FOXF1 | FOXQ1 | FOXA1 | FOXA2 | 3.28 |
| 8 | NaiveBcells | IRF1 | STAT3 | IRF4 | IRF2 | IRF8 | BCL6 | IRF3 | BCL11A | STAT2 | SPI1 | STAT5A | STAT1 | STAT5B | STAT4 | SPIB | PRDM1 | 3.28 |
| 8 | NaiveBcells | EP300 | 3.27 |
| 8 | NaiveBcells | NCOR2 | 3.27 |
| 8 | NaiveBcells | CREBBP | 3.27 |
| 8 | NaiveBcells | MEF2D | MEF2A | MEF2C | MEF2B | 3.25 |
| 8 | NaiveBcells | PAX5 | ZNF121 | 3.24 |
| 8 | NaiveBcells | SMARCA4 | 3.23 |
| 8 | NaiveBcells | NCOR1 | 3.23 |
| 9 | ActNK | POLR2A | 3.3 |
| 9 | ActNK | MBD3 | 3.29 |
| 9 | ActNK | TBX21 | ZNF18 | EOMES | TBR1 | TBX3 | TBX19 | T | TBX5 | 3.29 |
| 9 | ActNK | NFKB2 | HIVEP1 | RELA | RELB | REL | NFKB1 | 3.26 |
| 9 | ActNK | TET2 | 3.26 |
| 9 | ActNK | BRD4 | 3.26 |
| 9 | ActNK | GATA3 | TCF3 | ASCL2 | ZBTB18 | TFAP4 | LYL1 | ZEB1 | TCF12 | SNAI1 | TCF4 | NEUROG2 | MESP1 | GATA6 | GATA2 | NEUROD2 | FIGLA | BHLHA15 | SNAI2 | OLIG2 | TAL1 | GATA4 | GATA5 | NEUROD1 | ASCL1 | MYOG | MYOD1 | GATA1 | PTF1A | TWIST1 | 3.25 |
| 9 | ActNK | PHF8 | 3.25 |
| 9 | ActNK | HDAC1 | 3.25 |
| 9 | ActNK | AFF4 | 3.25 |

# Gene Track

### Gene track plot for each cluster

Gene track plot for each cluster. Here, CD3D nad MS4A1 are displayed as markers of T cells and B cells, respectively.

Copyright @2019 Liu lab
