# Supplementary material for: Integrative analyses of single-cell transcriptome and regulome using MAESTRO
Source: Genome Biol. 2020 Aug 7;21:198. doi: 10.1186/s13059-020-02116-x (PMC7412809; doi:10.1186/s13059-020-02116-x)
Supplement: Supplementary file 10 — Additional file 10. HTML output for the integrated analysis of scRNA-seq (12k cells) and scATAC-seq (10k cells) datasets of human PBMC from different donors using MAESTRO. [file 13059_2020_2116_MOESM10_ESM.html]

MAESTRO


MAESTRO

- scRNA-seq module
- scATAC-seq module
- Integration module

- Sample Information
- Integration
- Cell Clustering
- Annotation

# Sample Information

|  |  |
| --- | --- |
| Sample ID | 10X\_PBMC |
| scRNA Object Path | /home1/wangchenfei/Project/SingleCell/MAESTRO/10X\_PBMC\_12k\_scRNA/Result/Analysis/10X\_PBMC\_12k\_scRNA\_Object.rds |
| scATAC Object Path | /home1/wangchenfei/Project/SingleCell/MAESTRO/10X\_PBMC\_10k\_scATAC/Result/Analysis/10X\_PBMC\_10k\_scATAC\_Object.rds |

# Integration

### Cell alignment

UMAP visualization for joint clustering of scRNA-seq and scATAC-seq. Colors represent cells from different technologies. The cells are joined by CCA on gene expression level and regulatory potential from MAESTRO.

# Cell Clustering

### Cell clustering of scRNA-seq

UMAP visualization for only scRNA-seq after joint clustering. Colors represent original cluster IDs before integration.

### Cell clustering of scATAC-seq

UMAP visualization for only scATAC-seq after joint clustering. Colors represent original cluster IDs before integration.

# Annotation

### Celltype annotation based on alignment

UMAP visualization for joint clustering of scRNA-seq and scATAC-seq. Colors represent the cell types, which are generated using the scRNA-seq dataset and transferred to the scATAC-seq dataset.

Copyright @2019 Liu lab
