# Supplementary material for: Integrative analyses of single-cell transcriptome and regulome using MAESTRO
Source: Genome Biol. 2020 Aug 7;21:198. doi: 10.1186/s13059-020-02116-x (PMC7412809; doi:10.1186/s13059-020-02116-x)
Supplement: Supplementary file 11 — Additional file 11. Review history. [file 13059_2020_2116_MOESM11_ESM.docx]

Review History

**First round of review**

**Reviewer 1**

**Were you able to assess all statistics in the manuscript, including the appropriateness of statistical tests used?**

Yes, and I have assessed the statistics in my report.

**Were you able to directly test the software?**

Yes

**Comments to author:**

In the manuscript, Wang [et.al](http://et.al/) claimed that they have developed a computational workflow for the integrative analyses of scRNA-seq and scATAC-seq data, named Model-based AnalysEs of Transcriptome and RegulOme (MAESTRO). MAESTRO provides several basic functions, such as pre-processing, alignment, quality control, expression and chromatin accessibility quantification, clustering, differential analysis for scRNA-seq and scATAC-seq data. The workflow also includes some advanced functions, such as 1) integration analysis of scRNA-seq and scATAC-seq data 2) automatic cell-type annotation 3) interference of cluster-specific driver transcriptional regulators.

This Snakemake based workflow could be useful for researchers of related fields. However, if the authors want MAESTRO to be considered as a new algorithm/software, the novelty is largely lack. Most of the functions of MAESTRO can be replaced by other existing software. Actually, a lot of code of MAESTRO is directly from Seurat, SnapATAC, Cistrome-GO, RABIT, LISA, GIGGLE et. al, without significant modification or optimization. There are indeed some novel parts, for example, the regulatory potential (RP) score has not been used for the integration of scRNA-seq and scATAC-seq data. However, the advantage and robustness need to be demonstrated by more systematic comparison.

I think the authors have two ways to improve the manuscript.

Since this work adopts a lot of existing software and just rename it as MAESTRO, it will make more sense if MAESTRO is a web-based application. The previous work from the same group, Cistrome(Liu et al. 2011), which is highly cited, is a good example. Then the manuscript does not need to be significantly reorganized. And this Snakemake based workflow can be published together as a Local (Standalone) version.

If the authors want to propose MAESTRO as a novel algorithm, then the regulatory potential (RP) score-based integration, cell-type and transcription regulator annotation should be the focus. The current version of the manuscript makes the focus of this work not clear. Please move all the parts which are less important, or not novel into supplementary material. The following are my suggestions.

What does “model-based” mean in MAESTRO?

In the previous work from the same lab, the meaning of “model-based” is clear. For example, Model-based Analysis of ChIP-Seq (MACS) (Y. Zhang et al. 2008), the two key features of are: empirical modeling of 'd' and tag shifting by d/2 to putative protein-DNA interaction site; and the use of a dynamic λ local to capture local biases in the genome. There is an assumption of Poisson distribution and some parameters need to be estimated from data. However, in this work, “model-based” doesn't have a clear explanation. What is the distribution of the data? What parameters need to be estimated? The regulatory potential (RP) score from line 547 is only a simple formula. The authors need to explain what does the “model-based” mean exactly. Otherwise, it is better to remove “model-based” in the title.

Besides, the Regulatory Potential (RP) score is the key the author proposed to do integrated analysis between scRNA-seq and scATAC-seq data. Strangely, it is just simply mentioned at line 222 without further explanation in the main text. The Regulatory Potential (RP) score is worth to be introduced as a separate section. What special feature does this score have? What is the underlying logic? If only use the reads overlap with gene body to calculate Regulatory Potential (RP) score, whether the results will be significantly different?

There have been several published works support the integration of scRNA-seq and scATAC-seq data, which mainly include CoupledNMF(Duren et al. 2018), Seurat v3(Stuart et al. 2019) and LIGER(Welch et al. 2019). SnapATAC(Fang et al. 2019), another work primarily for clustering analysis of scATAC-seq alone, adopt CCA from Seurat v3 to integrate scRNA-seq and scATAC-seq data. About the label prediction of scATAC-seq from scRNA-seq, how the performance of MAESTRO compared to existing methods, in terms of accuracy and successful rate? This will be the key question most of the audience are care about.

Thus, the section of “Integrative analysis of scRNA-seq and scATAC-seq (line 266)" should be the most important part and Figure 5e, 5f should be the key figures of the manuscript. However, the description is somehow too brief. It is not clear how the author did the analysis. It is better to provide the code for the reviewers to reproduce Figure 5e and Figure 5f step-by-step.

Actually, in Figure 5e, the difference of NMI among MAESTRO, Seurat, and SnapATAC is so small. The description of "while the other three methods had relative lower NMIs and failed to align cells for rare populations such as pDCs or Mast cells” at line 307-308 is not supported well by Figure 5e and Figure S7 a-f. If compare Figure 5a, b with Figure S7 a-f, we can see for pDCc cells, Seurat and Cicero have very similar performance with MAESTRO. For MAST cells, all the methods have poor performance. It is better to make a table to show how many cells of scATAC-seq have successfully received labels from scRNA-seq for each cell type.

For Figure 5f, it is quite strange that the correlation between RNA and ATAC is consistently higher by MAESTRO across different cell types. “Pearson correlation coefficient” assumes Gaussian distribution which may not be fitted by the data. How about replacing the “Pearson correlation coefficient” by “Spearman's rank correlation coefficient”, which is nonparametric? Besides, it is always a good way to show correlation by scatterplot. I suggest the authors generate scatter plots colored by density to show the methods, in terms of accuracy and successful rate? This will be the key question most of the audience are care about.

Thus, the section of “Integrative analysis of scRNA-seq and scATAC-seq (line 266)" should be the most important part and Figure 5e, 5f should be the key figures of the manuscript. However, the description is somehow too brief. It is not clear how the author did the analysis. It is better to provide the code for the reviewers to reproduce Figure 5e and Figure 5f step-by-step.

Actually, in Figure 5e, the difference of NMI among MAESTRO, Seurat, and SnapATAC is so small. The description of "while the other three methods had relative lower NMIs and failed to align cells for rare populations such as pDCs or Mast cells” at line 307-308 is not supported well by Figure 5e and Figure S7 a-f. If compare Figure 5a, b with Figure S7 a-f, we can see for pDCc cells, Seurat and Cicero have very similar performance with MAESTRO. For MAST cells, all the methods have poor performance. It is better to make a table to show how many cells of scATAC-seq have successfully received labels from scRNA-seq for each cell type.

For Figure 5f, it is quite strange that the correlation between RNA and ATAC is consistently higher by MAESTRO across different cell types. “Pearson correlation coefficient” assumes Gaussian distribution which may not be fitted by the data. How about replacing the “Pearson correlation coefficient” by “Spearman's rank correlation coefficient”, which is nonparametric? Besides, it is always a good way to show correlation by scatterplot. I suggest the authors generate scatter plots colored by density to show the correlation between RNA and ATAC of at least 3 cell types by MAESTRO, SnapATAC, Seurat and Cicero prediction.

Besides, for the label transfer, Seurat v3 has provided a prediction score. Since SnapATAC and MAESTRO both adopt Seurat v3's framework, at least those two methods should also have a prediction score. What is the distribution of the prediction score of different methods? What are the NMI value and correlation if filter out those cells with low prediction scores?

At line 714-716, the authors mentioned: "We performed scRNA-seq and scATAC-seq integration using gene-body accessibility score from snapATAC, co-accessibility gene score from cicero, promoter and gene-body accessibility score from Seurat, as well as gene regulatory potential from MAESTRO”. However, the comparison should not limit to be only about “gene activity function”. There are two steps important for Seurat v3's integration. Firstly, in the "anchor identification" step, Seurat V3 construct a gene-by-cell matrix from scATAC-seq data based on the promoter and gene-body accessibility score, and then use Canonical correlation vectors (CCV) to project the two gene-by-cell matrixes into a correlated low-dimensional space, and calculate mutual nearest neighbors. Secondly, in the “anchor weighting” step, Seurat v3 set the parameter weight.reduction = atac[[‘‘lsi'']] to specify the previously computed LSI dimensional reduction from the peak-by-cell matrix of scATAC-seq when calculating anchor weights. SnapATAC uses a bin-by-cell matrix and applies diffusion maps for dimensional reduction, which will be different from LSI (Seurat V3). The authors only focus on “gene activity function” from different methods, which may make the comparison unfair. If the authors use SnapATAC ‘s whole solution to transfer labels, SnapATAC(gene-body accessibility score in “anchor identification”, and diffusion maps in “anchor weighting”) may show better performance than MAESTRO(gene regulatory potential in “anchor identification”, and LSI in “anchor weighting” ).

Also, since this is the most important part of this work, even the authors can get positive results from the PBMC datasets after the optimization of MAESTRO, they should do a more comprehensive comparison with 2-3 more datasets with larger size and higher complexity to make the conclusion more convincing.

For the "Automatic cell-type annotation" part, the author's solution is just different but not necessarily to be better. Several methods have been published very recently. For example, Garnett(Pliner, Shendure, and Trapnell 2019), CellAssign(A. W. Zhang et al. 2019). And another recently published work has done a comprehensive comparison of cell-type annotation methods(Abdelaal et al. 2019). How does the performance of MAESTRO compare to existing methods, especially the best-performing classifiers SVM, SVMrejection? The author can borrow the statistics F1-score and datasets of different complexity levels (Abdelaal et al. 2019) to do the comparison to demonstrate the advantage of the Regulatory Potential (RP) score regarding automatic cell-type annotation.

Besides, Table S2 and S3 in Additional file 3 are broken.

The section of “Transcriptional regulator inference", should also be the main focus of the manuscript. The author adopted the exiting method RABIT/LISA and GIGGLE to predict regulator from the scRNA-seq and scATAC-seq dataset, separately. However, at line 262-264, they mentioned: "Reassuringly, the regulators identified from scRNA-seq and scATAC-seq of the same cell type are highly consistent, indicating that MAESTRO can robustly predict the potential driver regulators from both scRNA-seq and scATAC-seq data (Figure 4e, f).”. This statement is misleading. If the regulators identified from scRNA-seq and scATAC-seq of the same cell type are highly consistent, then what is the benefit of doing both types of essays? The authors need to analyze 2-3 more datasets, check the results of more cell types. And then conclude that in how many percents of cases, the regulators from the two data types are consistent. And if the results are not consistent, the results from which type of data make more sense based on previous knowledge.

Besides, if MAESTRO use RABIT/LISA and GIGGLE to predict regulator based on the integration results from other methods, whether the regulator will be significant different?

In the section of “Comprehensive features of MAESTRO workflow”, the part of “Table 1 Comprehensive features of MAESTRO compared to other single-cell tools” (line 1204) is more like something in the supplementary material. Besides, the comparison is unfair because most of the software listed in Table 1 is not intended to be a comprehensive pipeline. It will be more appreciated that if the authors make a summary of which feature of MAESTRO is achieved by adapting existing software listed in Table1. For example, CCA analysis is adopted from Seurat.

At lines 106-108 the authors mentioned, "MAESTRO shows superior performance, in terms of less CPU-time and memory usage, in almost all the analysis steps and can handle large datasets while several other tools crashed due to memory overflow (Table S1 in Additional file 2)". If this is true, the computational efficiency of MAESTRO should be highlighted and presented more clearly. The different steps mentioned in Table S1, including Gene activity, Dimensionality reduction, Clustering, Differential peak, Cell type annotation, TF identification should be described separately and in more detail. The current statement, “in almost all the analysis steps”, is somehow misleading. Again, since many functions of MAESTRO are achieved by adopting other existing software, the authors should make it clear whether the higher efficiency of MAESTRO is purely benefited from the Snakemake system or not.

Besides, a recent study(Chen et al. 2019) suggests that, for scATAC-seq analysis, SnapATAC is the only method able to analyze a large dataset (> 80,000 cells). How does MAESTRO compare with SnapATAC in terms of scalability?

For the “Multiple levels of quality control” part, this whole section including Figure 2 (Pre-processing and quality control using MAESTRO) are something more like supplementary material.

For the “Clustering analysis” part, the Figure 3 (Benchmark of scATAC clustering performance on public datasets) have not revealed any new information compared to a recently published work (Chen et al. 2019). Besides, as mentioned at lines 202-204, clustering using candidate cis-regulatory elements (CCRE) catalog from the ENCODE project is not a robust solution. Thus, I suggest the authors move this part to supplementary material.

Reference

Abdelaal, Tamim et al. 2019. “A Comparison of Automatic Cell Identification Methods for Single-Cell RNA-Sequencing Data.” Genome Biology: 644435. https://genomebiology.biomedcentral.com/articles/10.1186/s13059-019-1795-z.

Chen, Huidong et al. 2019. “Assessment of Computational Methods for the Analysis of Single-Cell ATAC-Seq Data.” Genome Biology 20(1): 241. https://genomebiology.biomedcentral.com/articles/10.1186/s13059-019-1854-5 (January 12, 2020).

Duren, Zhana et al. 2018. “Integrative Analysis of Single-Cell Genomics Data by Coupled Nonnegative Matrix Factorizations.” Proceedings of the National Academy of Sciences of the United States of America 115(30).

Fang, Rongxin et al. 2019. “Fast and Accurate Clustering of Single Cell Epigenomes Reveals Cis-Regulatory Elements in Rare Cell Types.” bioRxiv: 615179. https://www.biorxiv.org/content/10.1101/615179v2 (July 25, 2019).

Liu, Tao et al. 2011. “Cistrome: An Integrative Platform for Transcriptional Regulation Studies.” Genome Biology 12(8): R83. http://genomebiology.biomedcentral.com/articles/10.1186/gb-2011-12-8-r83 (January 5, 2020).

Pliner, Hannah A., Jay Shendure, and Cole Trapnell. 2019. “Supervised Classification Enables Rapid Annotation of Cell Atlases.” Nature Methods: 538652. https://www.biorxiv.org/content/10.1101/538652v1 (September 11, 2019).

Stuart, Tim et al. 2019. “Comprehensive Integration of Single-Cell Data.” Cell 177(7): 1888-1902.e21. https://linkinghub.elsevier.com/retrieve/pii/S0092867419305598 (October 15, 2019).

Welch, Joshua D. et al. 2019. “Single-Cell Multi-Omic Integration Compares and Contrasts Features of Brain Cell Identity.” Cell 177(7): 1873-1887.e17. https://linkinghub.elsevier.com/retrieve/pii/S0092867419305045.

Zhang, Allen W. et al. 2019. “Probabilistic Cell-Type Assignment of Single-Cell RNA-Seq for Tumor Microenvironment Profiling.” Nature Methods: 1–9. http://www.nature.com/articles/s41592-019-0529-1 (September 11, 2019).

Zhang, Yong et al. 2008. “Model-Based Analysis of ChIP-Seq (MACS).” Genome Biology 9(9).

**Reviewer 2**

**Were you able to assess all statistics in the manuscript, including the appropriateness of statistical tests used?**

Yes, and I have assessed the statistics in my report.

**Were you able to directly test the methods?**

Yes

**Comments to author:**

In this study, the authors developed an integrated software tool MAESTRO for analyzing and integrating single-cell RNA-seq (scRNA-seq) and single-cell ATAC-seq (scATAC-seq) data. This software provides comprehensive functions for read alignment, quality control, clustering, cell type annotation, transcriptional regulator inference and cross-modality alignment and integration. The authors benchmarked some key functions of the software such as scATAC-seq clustering and scRNA-seq and scATAC-seq integration, and they also demonstrated the software using an analysis of bone marrow microenvironment in healthy donors and CLL patients. As there is increasing demands for single-cell transcriptome and regulome analysis, the development of MAESTRO is both timely and useful. The comprehensiveness of the analysis functions and the various benchmark analyses are the strengths of MAESTRO and this manuscript.

Major issues:

1.  While the comprehensive set of functions is a strength of MAESTRO, currently the software still has a lot of external dependencies. In order to use MAESTRO, one needs to install a number of other software tools including Miniconda3, Cell Ranger, RABIT/LISA and giggle in addition to the basic system requirements (Linux/Unix, Python, R). This is a lot of work for users. Depending on users' system and experience, the installation is not necessarily trivial. MAESTRO will be more useful if the authors can simplify the installation procedure.

2. Currently MAESTRO only supports Linux/Unix. It could have higher impact if the software can also be run on Windows operating system.

3. For bulk-level quality control, MAESTRO generates a number of QC statistics and figures such as those in Figure 2. However, in practice which cutoff should one use for each QC statistic to decide if a sample is low quality or not?

4. For single-cell level quality control, the authors described which cutoffs they used for analyzing data in this manuscript and mentioned that these QC parameters are configurable and can be tuned. It would be useful if they can be more concrete and tell potential users how to tune and choose the QC cutoffs for users' own data. It might be useful to provide information on the normal ranges of QC statistics.

5. For scATAC-seq QC, Figure 2f shows that the high-quality and low-quality cells form distinct clusters when plotted using "Reads passed filters" and "Fraction of reads in promoter". This plot is used to demonstrate MAESTRO's ability to select high-quality cells. However, is this "clear separation" between high- and low-quality cells always true or is it only specific to this example dataset? In order to show that this "clear separation" is generally useful for QC, it is important to show that it is a general pattern in multiple independent datasets.

6. For scATAC-seq clustering analysis, chromVAR was not benchmarked in the manuscript but it is incorporated into MAESTRO. In contrast, snapATAC-seq was benchmarked but it is not incorporated into MAESTRO. Can authors explain why?

7. The most difficult part for automatic cell type annotation actually is compiling marker genes for each cell type. Currently, MAESTRO only provides marker genes for immune cell types. It will be more useful if marker genes for other major cell types could also be provided as a reference database.

8. For integrating scRNA-seq and scATAC-seq, the authors mentioned that the other three methods (snapATAC, cicero, Seurat) failed to align cells for rare cell populations such as pDCs or Mast cells (Figure S7a-f). However, they did not mention how MAESTRO performed in those rare cell populations. Indeed, Figure 5a-b seem to suggest that MAESTRO also failed to align Mast cells. Moreover, interpretation of Figure S7a-f and Figure 5a-b using human eyes could be misleading. For example, if blue data points are plotted after plotting the red data points, it is possible that the red data points in certain regions of the UMAP plot are all covered by blue data point. As a result, one cannot see any red dots by eye in these regions (e.g., pDC, Mast) even though both blue and red data points exist and mix/align well. Thus, it is important to "quantify" the overlap between scRNA-seq and scATAC-seq in order to convincingly compare different methods' ability to align cells for rare cell populations.

9. In Figure 5e and Figure S8d, the improvement of MAESTRO over Seurat and snapATAC in terms of NMI seems to be small. However, in Figure 5f and Figure S8c, the improvement of MAESTRO over Seurat and snapATAC in terms of correlation between ATAC and RNA gene expression seems to be much bigger. It is unclear how much of the improvement in Figure 5f and Figure S8c is attributed to the improved cell alignment (which is what NMI tries to measure) and how much of the improvement is simply caused by the improved correlation between the regulatory potential (RP) and gene expression conditional on fixing cell alignment. It will be useful to decompose Figure 5f (and similarly Figure S8c) into two figures. In the first figure, scRNA-seq and scATAC-seq are aligned using different methods (MAESTRO, Seurat, snapATAC, cicero), and for each alignment method the correlation between ATAC and RNA is computed using the RP and gene expression (even if the RP is not used in aligning the cells for Seurat, snapATAC and cicero). These four correlations are then compared. This figure will show the pure gain of MAESTRO over the other methods in terms of cell alignment. In the second figure, using the cell alignment provided by MAESTRO, compute and compare the correlation between ATAC and RNA using four different ways to summarize ATAC data (RP by MAESTRO, promoter and gene-body accessibility score from Seurat, gene-body accessibility score from snapATAC, and co-accessibility gene score from cicero). This figure will show the pure effect of using RP in improving the correlation between the RNA-seq gene expression measurements and ATAC-seq chromatin accessibility measurements.
Minor issues:

10. While MAESTRO is an abbreviation for Model-based AnalysEs of Transcriptome and RegulOme, it is unclear which part of the software represents "Model-based". Most functions of the software are implemented by calling other existing tools. This is fine from the perspective of developing useful software. However, for naming the software, are there any new "models" developed here that is unique for MAESTRO? If there are not, using the word "model-based" is somewhat confusing and misleading.

11. Related to the above comment, Table 1 is very useful for comparing functions of MAESTRO with other methods. It would be also useful to annotate which of the functions in MAESTRO are based on calling existing methods and software tools (e.g. alignment, etc.), and which of the functions are newly developed in MAESTRO and therefore represent new methods. This will be helpful for highlighting the methodological novelties of MAESTRO.  

12. How many healthy donors and CLL patients are used in the CLL example? The description in the method seems to suggest that there is only one healthy donor and one patient. However, throughout the paper, the authors used the plural form "healthy donors" and "patients" instead of "healthy donor" and "patient".

13. Line 569 "marker genes divided by log length of marker genes": here the meaning of "length" is unclear. Does it refer to the length of gene body (in the unit of base pairs) or do you mean the total number of marker genes?

14. Figure S8: the text description for S8(c) and S8(d) should be switched. Also, for S8(b), the dot size represents the fraction of cells that have a "higher" regulatory potential level. How is "higher" defined? What is the cutoff?

15. There are a number of typos in the manuscript. For example
1) Line 497: "Principle Component Analysis" should be "Principal Component Analysis".
2) Line 539: "minimum presence faction" should be "minimum presence fraction".
3) Line 569: "devided" should be "divided".
4) Figure 1: in the Analysis box of the figure, "Exprssion Index" should be "Expression Index".

**Authors Response**

**Point-by-point responses to the reviewers’ comments:**

We appreciate the reviewers’ time and effort in reviewing our manuscript. Their constructive feedbacks have enabled us to substantially improve our work. We updated the MAESTRO workflow extensively, conducted more analyses, and reorganized the manuscript to highlight the novelties of MAESTRO. The major changes were summarized as follows.

- We improved the speed of reads alignment, preprocessing and downstream analysis by updating several MAESTRO components to preferable software, provided a user-friendly command-line interface for finetuning parameters, simplified the installation of all MAESTRO components within the conda environment, and added detailed tutorials in the online MAESTRO documentation.
- We conducted additional analyses and tested more datasets to demonstrate the superior performance of MAESTRO compared to existing software in 1) integrating scATAC-seq and scRNA-seq, and 2) automatically annotating cell-types. We also added more analysis and clarified the regulatory potential model in MAESTRO.
- We reorganized our manuscript significantly, moved most of the descriptions related to quality control and clustering evaluation into methods and supplementary materials. Instead, we highlighted the novel features of MAESTRO in the manuscript, including scATAC-seq/scRNA integration based on regulatory potential, automatic cell-type annotation, as well as inference of cluster-specific transcription regulators.

*Reviewer #1: In the manuscript, Wang* [*et.al*](http://et.al/) *claimed that they have developed a computational workflow for the integrative analyses of scRNA-seq and scATAC-seq data, named Model-based AnalysEs of Transcriptome and RegulOme (MAESTRO). MAESTRO provides several basic functions, such as pre-processing, alignment, quality control, expression and chromatin accessibility quantification, clustering, differential analysis for scRNA-seq and scATAC-seq data. The workflow also includes some advanced functions, such as 1) integration analysis of scRNA-seq and scATAC-seq data 2) automatic cell-type annotation 3) inference of cluster-specific driver transcriptional regulators.*

*This Snakemake based workflow could be useful for researchers of related fields. However, if the authors want MAESTRO to be considered as a new algorithm/software, the novelty is largely lack. Most of the functions of MAESTRO can be replaced by other existing software. Actually, a lot of code of MAESTRO is directly from Seurat, SnapATAC, Cistrome-GO, RABIT, LISA, GIGGLE et. al, without significant modification or optimization. There are indeed some novel parts, for example, the regulatory potential (RP) score has not been used for the integration of scRNA-seq and scATAC-seq data. However, the advantage and robustness need to be demonstrated by more systematic comparison.*

*I think the authors have two ways to improve the manuscript.*

*a)	Since this work adopts a lot of existing software and just rename it as MAESTRO, it will make more sense if MAESTRO is a web-based application. The previous work from the same group, Cistrome(Liu et al. 2011), which is highly cited, is a good example. Then the manuscript does not need to be significantly reorganized. And this Snakemake based workflow can be published together as a Local (Standalone) version.*

Single-cell datasets are often too large for web transfer and storage, and analyses take significant computational resources. These factors make web applications impractical, so we chose the stand-alone Snakemake workflow, as it is practical and more efficient for the users.

*b)	If the authors want to propose MAESTRO as a novel algorithm, then the regulatory potential (RP) score-based integration, cell-type and transcription regulator annotation should be the focus. The current version of the manuscript makes the focus of this work not clear. Please move all the parts which are less important, or not novel into supplementary material. The following are my suggestions.*

We appreciate the reviewer for seeing the promise and interest of MAESTRO to the community. We fully agree that the novelties of MAESTRO lie in the integration of scATAC-seq with scRNA-seq using regulatory potential (RP) and the automatic inference of cell-type and transcription regulators. To highlight these, we significantly reorganized the manuscript according to reviewers’ suggestions, added more systematic analyses to demonstrate the robustness, and conducted systematic comparisons to the existing software on three independent datasets, PBMC from different donors, PBMC from the same donor, and BMMC from the same donor. In addition, using a large and complex scATAC-seq dataset from basal cell carcinoma (BCC) patients, we demonstrated the scalability of MAESTRO. Furthermore, we optimized the performance of MAESTRO by accelerating read alignment, pre-processing, and analysis steps, and improved the usability by adding detailed descriptions and documentation for the parameters and significantly simplifying the installation. We hope the revised manuscript and updated MAESTRO workflow will be much more useful to the research community.

*1. What does “model-based” mean in MAESTRO?*

*In the previous work from the same lab, the meaning of “model-based” is clear. For example, Model-based Analysis of ChIP-Seq (MACS) (Y. Zhang et al. 2008), the two key features of are: empirical modeling of 'd' and tag shifting by d/2 to putative protein-DNA interaction site; and the use of a dynamic λ local to capture local biases in the genome. There is an assumption of Poisson distribution and some parameters need to be estimated from data. However, in this work, “model-based” doesn't have a clear explanation. What is the distribution of the data? What parameters need to be estimated? The regulatory potential (RP) score from line 547 is only a simple formula. The authors need to explain what does the “model-based” mean exactly. Otherwise, it is better to remove “model-based” in the title.*

*Besides, the Regulatory Potential(RP) score is the key the author proposed to do integrated analysis between scRNA-seq and scATAC-seq data. Strangely, it is just simply mentioned at line 222 without further explanation in the main text. The Regulatory Potential(RP) score is worth to be introduced as a separate section. What special feature does this score have? What is the underlying logic? If only use the reads overlap with gene body to calculate Regulatory Potential(RP) score, whether the results will be significantly different?*

The “model-based” in MAESTRO represents the regulatory potential model for estimating the gene activity score from scATAC-seq, which is the key step to annotate the scATAC-seq clusters and to integrate with scRNA-seq. The presence of scATAC-seq peaks surrounding each gene reflects the potential transcription regulator (TR) bindings and their impact on the gene expression. The existing methods such as Seurat and snapATAC use distance-based thresholds to evaluate the effect of adjacent scATAC-seq peaks on gene activity. For which if the threshold were 100kb, a peak at the transcription start site (TSS) would have the same regulatory effect as a peak 100kb away from the TSS. Our regulatory potential model assumes that the influence of a peak on the expression of a gene decreases monotonically as a function of distance to decay center, which can be either the TSS of that gene or center of gene body, and the influence of multiple peaks are addictive. We consider a smooth decay function to be a more reasonable model than a binary distance cut-off, as Hi-C chromatin interaction data demonstrated the decreased interaction frequency with increased genomic proximity (Fig 2A in Lieberman-Aiden et al, Science 2009). The parameter to be evaluated is the half-decay distance d0 between the TR binding sites and the TSS. We classified the TR regulations into two categories, for the promoter-dominant regulations, we set the half-decay distance d0 as 1kb (TSS 1K); and for the enhancer-dominant regulations, the half-decay distance d0 was set to 10kb (TSS 10K). Per reviewer’s suggestions, we also tested peak overlap with gene body to calculate RP score, which calculates the distance between scATAC-seq peaks and the center of gene body as di, with the half-decay distance d0 set as 10 kb (Genebody 10K).

Figure R1. Comparison of integration performance using the regulatory potential model based on promoter-dominant regulation, enhancer-dominant regulation and gene-body centered regulation on three datasets.

(a) Distribution of cell-type prediction scores after integration. The mean of the prediction scores and the number of cells with a high prediction (>0.5) are labeled in the legends.
(b) NMI between scATAC-seq clusters and cell-type labels transferred from scRNA-seq.
(c) Pearson’s correlation coefficients between expression level from scRNA-seq and regulatory potential from scATAC-seq within each cluster.

To evaluate the performance of the regulatory potential model using different half-decay distances, we calculated the regulatory potential using the above strategies and integrated the matched scATAC-seq and scRNA-seq for three different datasets (Figure R1). The cell-type labels were transferred from scRNA-seq using Seurat v3. For the three datasets tested, while the three models have similar performance on NMI of scATAC-seq cluster transferred cell types, the TSS 10K regulatory potential model has significantly better correlations between scATAC-seq and scRNA-seq. These results suggest that peaks from scATAC-seq mainly reflect enhancer-dominant regulations. In reality, there are not always corresponding scRNA-seq for users to estimate the proper half-decay distance. Considering the overall good performance of TSS 10K regulatory potential model, we set TSS 10K as the default model in MAESTRO, but allow users to pick other models. We discussed the above analysis and further clarified our regulatory potential model in the revised manuscript and supplementary materials section B.

*2. There have been several published works support the integration of scRNA-seq and scATAC-seq data, which mainly include CoupledNMF(Duren et al. 2018), Seurat v3(Stuart et al. 2019) and LIGER(Welch et al. 2019). SnapATAC(Fang et al. 2019), another work primarily for clustering analysis of scATAC-seq alone, adopt CCA from Seurat v3 to integrate scRNA-seq and scATAC-seq data. About the label prediction of scATAC-seq from scRNA-seq, how the performance of MAESTRO compared to existing methods, in terms of accuracy and successful rate? This will be the key question most of the audience are care about.  

Thus, the section of “Integrative analysis of scRNA-seq and scATAC-seq (line 266)" should be the most important part and Figure 5e, 5f should be the key figures of the manuscript. However, the description is somehow too brief. It is not clear how the author did the analysis. It is better to provide the code for the reviewers to reproduce Figure 5e and Figure 5f step-by-step.*

In the revised manuscript, we conducted additional analysis to demonstrate that the integration based on MAESTRO regulatory potential model are robust and have better performance than the existing tools. All the sample datasets and codes for evaluating MAESTRO performance could be accessed from <https://github.com/chenfeiwang/MAESTRO_benchmark>. We also clarified the benchmarking details in the revised method section.

*Actually, in Figure 5e, the difference of NMI among MAESTRO, Seurat, and SnapATAC is so small. The description of "while the other three methods had relative lower NMIs and failed to align cells for rare populations such as pDCs or Mast cells” at line 307-308 is not supported well by Figure 5e and Figure S7 a-f. If compare Figure 5a, b with Figure S7 a-f, we can see for pDCc cells, Seurat and Cicero have very similar performance with MAESTRO. For MAST cells, all the methods have poor performance. It is better to make a table to show how many cells of scATAC-seq have successfully received labels from scRNA-seq for each cell type.*

We quantified the number of cells in scATAC-seq that received transferred labels from scRNA-seq for three different datasets (Table R1 and Table S5 in Additional File 7). While there is no ground truth for the number of cells for each cell-type in scATAC-seq, we hypothesis that the cell-type proportion in scATAC-seq should be roughly similar with scRNA-seq from the same sample types and estimated the expected number accordingly. We then compared the number of cells that successfully received labels for each cell-type in scATAC-seq with the expectations, and calculated the cumulative error by adding up the normalized errors for each cell-type.

For all the three tested dataset, MAESTRO, Seurat and snapATAC shows better performance than cicero, while for PBMC Different Donors and BMMC same Donor dataset, MAESTRO have the lowest cumulative error among all the methods. For PBMC Different Donors dataset, cicero failed to align Memory B-cells, CD 16 Monocytes and DC cells, and snapATAC failed to align pDC cells. All of the tested methods failed to align CD4 Naïve T-cells, Macrophage and Mast cells, while the number of CD14 Monocytes is much higher than expected. These analysis indicated the chromatin landscape of Macrophage/Mast cells might resemble other innate immune cells such as Monocytes thus cannot be correctly assigned using any current strategies. We included the detailed tables for the three datasets and discussed the above analysis in the revised manuscript.

**Dataset 1: PBMC Different Donors**

| Methods | B Naive | B Memory | CD14 Mono | CD16 Mono | CD4  Naive T | CD4 Memory T | CD8  Naïve T | CD8 Activated T | NK | DC | pDC | Macrophage | Mast | Cumulative error |
| --- | --- | --- | --- | --- | --- | --- | --- | --- | --- | --- | --- | --- | --- | --- |
| Expected | 878 | 419 | 1776 | 241 | 335 | 2228 | 834 | 1333 | 346 | 375 | 72 | 58 | 19 | 0.00 |
| MAESTRO | 366 | 232 | 3829 | 233 | 0 | 1557 | 417 | 1209 | 470 | 148 | 64 | 1 | 0 | 157.17 |
| Seurat | 348 | 238 | 3842 | 229 | 0 | 1541 | 410 | 1230 | 467 | 101 | 63 | 0 | 0 | 160.58 |
| snapATAC | 331 | 236 | 3738 | 220 | 0 | 1516 | 391 | 1251 | 459 | 135 | 0 | 0 | 0 | 165.21 |
| cicero | 561 | 22 | 4339 | 6 | 0 | 1431 | 398 | 1128 | 459 | 3 | 62 | 0 | 0 | 200.39 |

**Dataset 2: PBMC Same Donor**

| Methods | B Naive | CD14 Mono | CD16 Mono | CD4 Naive T | CD4 Memory T | CD8 T | NK | DC | pDC | Cumulative error |
| --- | --- | --- | --- | --- | --- | --- | --- | --- | --- | --- |
| Expected | 571 | 2740 | 921 | 1999 | 1011 | 1878 | 674 | 171 | 72 | 0.00 |
| MAESTRO | 769 | 3541 | 353 | 1810 | 666 | 1615 | 568 | 159 | 69 | 68.81 |
| Seurat | 769 | 3446 | 358 | 1665 | 937 | 1595 | 581 | 237 | 70 | 65.52 |
| snapATAC | 750 | 3061 | 414 | 1539 | 832 | 1557 | 579 | 178 | 71 | 57.97 |
| cicero | 767 | 4260 | 0 | 1868 | 0 | 675 | 531 | 0 | 0 | 157.14 |

**Dataset 3: BMMC Same Donor**

| Methods | CD4 T | CD8 Naïve T | CD8 Activated T | NK | CLL1 | CLL2 | Early B | Pre-pro B | DC | Mast | MastPro | Mono | Cumulative error |
| --- | --- | --- | --- | --- | --- | --- | --- | --- | --- | --- | --- | --- | --- |
| Expected | 1907 | 969 | 325 | 549 | 2526 | 485 | 720 | 171 | 180 | 119 | 187 | 573 | 0.00 |
| MAESTRO | 1636 | 709 | 116 | 451 | 2456 | 234 | 682 | 149 | 1 | 139 | 224 | 772 | 72.42 |
| Seurat | 1554 | 409 | 193 | 516 | 1953 | 338 | 343 | 145 | 72 | 113 | 210 | 739 | 86.13 |
| snapATAC | 1525 | 857 | 30 | 320 | 2354 | 166 | 632 | 152 | 62 | 110 | 178 | 690 | 76.29 |
| cicero | 1583 | 478 | 84 | 340 | 2283 | 29 | 458 | 0 | 51 | 7 | 223 | 813 | 126.40 |

Table R1. The number of cells in scATAC-seq that successfully received transferred labels from scRNA-seq for each cell type (prediction score > 0.5). The first row represent number of cells that are expected in each cell-type of scATAC-seq dataset, which is estimated by the total number of cells in scATAC-seq multiplied by the proportion of each cell-type from corresponding scRNA-seq dataset. The last column represent the cumulated error for different methods, for which is calculated by adding up the difference between observation and expectation, normalized by the number of expectation for each cell-type.

*For Figure 5f, it is quite strange that the correlation between RNA and ATAC is consistently higher by MAESTRO across different cell types. “Pearson correlation coefficient” assumes Gaussian distribution which may not be fitted by the data. How about replacing the “Pearson correlation coefficient” by “Spearman's rank correlation coefficient”, which is nonparametric? Besides, it is always a good way to show correlation by scatterplot. I suggest the authors generate scatter plots colored by density to show the correlation between RNA and ATAC of at least 3 cell types by MAESTRO, SnapATAC, Seurat and Cicero prediction.*

Based on this reviewer’s suggestion, we showed the spearman’s correlation between gene expression level from scRNA-seq and gene activity scores from scATAC-seq using MAESTRO, Seurat, snapATAC and cicero. The results indicate that MAESTRO and snapATAC have similar performance and both are better than Seurat and cicero (Figure R2a,b). However, when only focused on expressed genes with an averaged log2 UMI of scRNA-seq larger than 0.5, only the score from MAESTRO’s regulatory potential model showed a positive correlation with gene expression level, regardless of using Pearson or Spearman’s correlation (Figure R2 c-e). This indicates that only MAESTRO’s regulatory potential model has the ability to estimate the expression magnitudes. We discussed these points in the revised manuscript, and also showed the correlation using scatterplots.

Figure R2 Correlation between gene expression level from scRNA-seq and gene activity score from scATAC-seq.

(a) Spearman’s correlation coefficients between expression level from scRNA-seq and gene activity score from scATAC-seq in each cluster.

(b) Scatter plot for visualizing the average gene expression level and average gene activity score for all genes in B Memory cells from PBMC Different Donors dataset. The x-axis represents the log2 UMI count from scRNA-seq, the y-axis represents the log2 gene activity score from scATAC-seq. Both Pearson and Spearman’s correlation is labeled.

(c) Pearson’s correlation coefficients between expression level from scRNA-seq and gene activity score from scATAC-seq in each cluster, only calculated for expressed genes (averaged log2 UMI > 0.5 in scRNA-seq).

(d) Spearman’s correlation coefficients between expression level from scRNA-seq and gene activity score from scATAC-seq in each cluster, only calculated for expressed genes.

(e) Scatter plot for only the expressed genes in CD8 Activated T-cells from PBMC Different Donors dataset.

*Besides, for the label transfer, Seurat v3 has provided a prediction score. Since SnapATAC and MAESTRO both adopt Seurat v3's framework, at least th**ose two methods should also have a prediction score. What is the distribution of the prediction score of different methods? What are the NMI value and correlation if filter out those cells with low prediction scores?*

We thank the reviewer for this helpful suggestion. We generated the distribution of prediction scores for both MAESTRO, Seurat, snapATAC and cicero using three independent datasets. The integration using MAESTRO regulatory potential model has the overall higher prediction score in all the three tested datasets compared with the other three software (Figure R3). In terms of consistency between scATAC-seq and scRNA-seq clusters, MAESTRO shows slightly higher NMI than Seurat and snapATAC, and all three have better NMI than cicero. We defined the cells with a prediction score greater than 0.5 as the high-quality predicted (HP) cells. Filtering low-quality cells improved the NMI (Fig R3 b-c) for all the methods, so that MAESTRO, Seurat, snapATAC have similar performance, but at the cost of eliminating more cells in the other methods compared to MAESTRO. In addition, filtering low-quality cells did not significantly improve the correlation between the gene expression level and the gene activity score (Fig R3 d-e). Therefore, we conclude that MAESTRO has the overall best performance in integrating scATAC-seq with scRNA-seq. We included the analysis using all cells in the revised manuscript.

Figure R3 Comparisons of label prediction score using MAESTRO regulatory potential, Seurat score, snapATAC gene activity and cicero co-accessibility.

(a) Distribution of label prediction score for three different datasets after integration using Seurat v3 CCA analysis on different gene scoring methods. Mean of the prediction scores and the number of cells with a high prediction (>0.5) are labeled in the legends.

(b) Raw NMI between scATAC-seq clusters and cell-type labels transferred from scRNA-seq.

(c) NMI of only the high-quality predicted cells (> 0.5).

(d) Pearson’s correlation coefficients between expression level from scRNA-seq and gene activity score from scATAC-seq within each cluster.

(e) Pearson’s correlation coefficients of only the high-quality predicted cells (> 0.5).

*At line 714-716, the authors mentioned: "We performed scRNA-seq and scATAC-seq integration using gene-body accessibility score from snapATAC, co-accessibility gene score from cicero, promoter and gene-body accessibility score from Seurat, as well as gene regulatory potential from MAESTRO”. However, the comparison should not limit to be only about “gene activity function”. There are two steps important for Seurat v3's integration. Firstly, in the "anchor identification" step, Seurat V3 construct a gene-by-cell matrix from scATAC-seq data based on the promoter and gene-body accessibility score, and then use Canonical correlation vectors (CCV) to project the two gene-by-cell matrixes into a correlated low-dimensional space, and calculate mutual nearest neighbors. Secondly, in the “anchor weighting” step, Seurat v3 set the parameter weight.reduction = atac[[‘‘lsi'']] to specify the previously computed LSI dimensional reduction from the peak-by-cell matrix of scATAC-seq when calculating anchor weights. SnapATAC uses a bin-by-cell matrix and applies diffusion maps for dimensional reduction, which will be different from LSI (Seurat V3). The authors only focus on “gene activity function” from different methods, which may make the comparison unfair. If the authors use SnapATAC ‘s whole solution to transfer labels, SnapATAC(gene-body accessibility score in “anchor identification”, and diffusion maps in “anchor weighting”) may show better performance than MAESTRO(gene regulatory potential in “anchor identification”, and LSI in “anchor weighting” ).*

We performed the integration analysis following the reviewer’s suggestions. For snapATAC, we performed dimension reduction using diffusion maps, clustered the cells based on the Leiden algorithm and calculated the gene-body accessibility. We then integrated the scATAC-seq with scRNA-seq by setting diffusion maps for anchor weighting. The snapATAC integration performance was similar for the PBMC datasets, but even worse than using LSI as anchor weighting on the BMMC dataset Figure R4a-c). After label transferring using MAESTRO RP model or snapATAC-seq gene activity, we selected the CD8 T Activated cells from both scRNA-seq and scATAC-seq, then calculated the correlations between the gene expression level and the gene activity score from MAESTRO regulatory potential model and gene-accessibility score from snapATAC. The result showed that MAESTRO RP has a higher Pearson correlation, whether or not the CD8 T cells are defined by MAESTRO (Figure R4d) or snapATAC (Figure R4e). Taken together, these analyses suggested that the higher consistency of MAESTRO regulatory potential scores with gene expression was independent of cell alignment. In the revised manuscript, we still used LSI in “anchor weighting” for all the methods.

Figure R4. Integration of BMMC Same Donor scRNA-seq and scATAC-seq using snapATAC clustering and Seurat integration.

(a) UMAP visualization for joint clustering of BMMC scRNA-seq and scATAC-seq. The color represents cells from different technologies.

(b) UMAP visualization for joint clustering of BMMC scRNA-seq and scATAC-seq. The color represents cell-type labels transferred from scRNA-seq.

(c) Distribution of label prediction after integration using Seurat v3 CCA analysis on different methods. Mean of the prediction scores and the number of cells with a high prediction (>0.5) were labeled in the legends.

(d) Correlation comparison of MAESTRO-defined CD8 Activated T cells.

(e) Correlation comparison of snapATAC-defined CD8 Activated T cells.

*Also, since this is the most important part of this work, even the authors can get positive results from the PBMC datasets after the optimization of MAESTRO, they should do a more comprehensive comparison with 2-3 more datasets with larger size and higher complexity to make the conclusion more convincing.*

Besides the original PBMC Different Donor (12K scRNA-seq and 10K scATAC-seq) and BMMC Same Donor (5K scRNA-seq and 9K scATAC-seq) datasets, we demonstrated the performance of MAESTRO on additional inhouse PBMC Same Donor (2K scRNA-seq and 10K scATAC-seq) dataset, and public dataset from BCC patients (53K scRNA-seq and 38K scATAC-seq, Satpathy et al., 2019, Yost et al., 2019). The results can be found in the last section of the revised manuscript.

*3. For the "Automatic cell-type annotation" part, the author's solution is just different but not necessarily to be better. Several methods have been published very recently. For example, Garnett(Pliner, Shendure, and Trapnell 2019), CellAssign(A. W. Zhang et al. 2019). And another recently published work has done a comprehensive comparison of cell-type annotation methods(Abdelaal et al. 2019). How does the performance of MAESTRO compare to existing methods, especially the best-performing classifiers SVM, SVMrejection? The author can borrow the statistics F1-score and datasets of different complexity levels (Abdelaal et al. 2019) to do the comparison to demonstrate the advantage of the Regulatory Potential (RP) score regarding automatic cell-type annotation. 

Besides, Table S2 and S3 in Additional file 3 are broken.*

The study from Abdelaal et al.2019 classified the annotation tools into two categories: (1) supervised methods that require a labeled training dataset, such as SVM and SVMrejection; (2) tools based on prior-knowledge, such as using marker genes or pre-trained classifiers. As MAESTRO relies on user-defined marker genes to annotate the cell-type, we focused on the comparison with the second category of tools such as SCINA and Garnett. We used the sample dataset from the Abdelaal et al., 2019, which is a sorted PBMC dataset with known cell-type labels. We found that MAESTRO showed better performance in annotating the immune cell-types using the LM22 gene signature from Newman et al., 2015 (Figure R5). We included these analyses in the revised manuscript, and also updated the Table S2 and S3.

Figure R5. Comparison of the performance on automatic cell-type annotation using a dataset from sorted PBMCs.

(a) UMAP visualization of true labels, MAESTRO annotated labels, SCINA annotated labels and GarnettCV annotated labels on sorted PBMC dataset from Zheng et al.

(b) Median F1-score for annotation using different methods. For Garnett, we used both the LM22 signatures and the simplified signatures from the original study.

*4. The section of “Transcriptional regulator inference", should also be the main focus of the manuscript. The author adopted the exiting method RABIT/LISA and GIGGLE to predict regulator from the scRNA-seq and scATAC-seq dataset, separately. However, at line 262-264, they mentioned: "Reassuringly, the regulators identified from scRNA-seq and scATAC-seq of the same cell type are highly consistent, indicating that MAESTRO can robustly predict the potential driver regulators from both scRNA-seq and scATAC-seq data (Figure 4e, f).”. This statement is misleading. If the regulators identified from scRNA-seq and scATAC-seq of the same cell type are highly consistent, then what is the benefit of doing both types of essays? The authors need to analyze 2-3 more datasets, check the results of more cell types. And then conclude that in how many percents of cases, the regulators from the two data types are consistent. And if the results are not consistent, the results from which type of data make more sense based on previous knowledge. 

Besides, if MAESTRO use RABIT/LISA and GIGGLE to predict regulator based on the integration results from other methods, whether the regulator will be significant different?*

We thank the reviewer for this important suggestion. With MAESTRO, we are able to predict the driver regulators for each cluster in scRNA-seq and scATAC-seq separately, and combine the predictions from the same cell-type after integration. While the major regulators from different methods might be similar, there could be biologically relevant differences. We compared the transcription factors predicted by LISA on scRNA-seq and predicted by GIGGLE on scATAC-seq for the same cell-type in PBMC Different Donor dataset (Figure R6). The results are overall consistent, indicating both methods have the potential power to identify driver regulators in corresponding technology. There are also interesting differences between the two approaches. For example, RXRA was only predicted to be a regulator from scATAC-seq in CD14 Monocytes, and HDAC3 was only predicted to be a regulator from scATAC-seq in CD8 Naïve T-cells. RXRA is known to control the innate inflammatory response through the upregulation of chemokine expression in monocytes (Nunez et al., 2010), while HDAC3 has been reported to restrain the CD8-lineage gene expression and maintain a bi-potential state for CD4+CD8+ cells (Phillips et al., 2019). Those examples demonstrate that the transcription regulators predicted from scATAC-seq are sufficiently meaningful and can complement the predictions from scRNA-seq. We discussed the above analysis in the revised manuscript.

Figure R6. Comparison of transcription regulator prediction using scRNA-seq and scATAC-seq.

(a) Correlation of the LISA predicted TF enrichment score and GIGGLE predicted TF enrichment score.

(b) Visualization of predicted TFs in PBMC Different Donor CD14 Monocyte cluster.

(c) Visualization of predicted TFs in PBMC Different Donor CD8 Naïve T-cell cluster.

*5. In the section of “Comprehensive features of MAESTRO workflow”, the part of “Table 1 Comprehensive features of MAESTRO compared to other single-cell tools” (line 1204) is more like something in the supplementary material. Besides, the comparison is unfair because most of the software listed in Table 1 is not intended to be a comprehensive pipeline. It will be more appreciated that if the authors make a summary of which feature of MAESTRO is achieved by adapting existing software listed in Table1. For example, CCA analysis is adopted from Seurat.*

*At lines 106-108 the authors mentioned, "MAESTRO shows superior performance, in terms of less CPU-time and memory usage, in almost all the analysis steps and can handle large datasets while several other tools crashed due to memory overflow (Table S1 in Additional file 2)". If this is true, the computational efficiency of MAESTRO should be highlighted and presented more clearly. The different steps mentioned in Table S1, including Gene activity, Dimensionality reduction, Clustering, Differential peak, Cell type annotation, TF identification should be described separately and in more detail. The current statement, “in almost all the analysis steps”, is somehow misleading. Again, since many functions of MAESTRO are achieved by adopting other existing software, the authors should make it clear whether the higher efficiency of MAESTRO is purely benefited from the Snakemake system or not.*

*Besides, a recent study(Chen et al. 2019) suggests that, for scATAC-seq analysis, SnapATAC is the only method able to analyze a large dataset (> 80,000 cells). How does MAESTRO compare with SnapATAC in terms of scalability?*

In the revised manuscript, we added a table to summarize the tools incorporated into MAESTRO (Table R2 and Table S2 in the Additional file 4). Besides, we discussed how we optimized MAESTRO to achieve higher scalability and faster speed as follows.

- In the gene score calculation step, the R-based framework of Seurat and Cicero might fail due to memory overflow for large datasets. We optimized the regulatory potential calculation in MAESTRO by implementing the matrix manipulation function in Python, which can handle scATAC-seq with 38K cells and ~500K peaks on a computer with 380GB memory.
- MAESTRO stores all the data in memory as a sparse matrix and saves data to disk in HDF5 format, which is scalable and memory-efficient when performing clustering and differential analysis.
- To improve the time and memory efficiency of differential analysis, the updated MAESTRO workflow used Presto (<https://github.com/immunogenomics/presto>) for Wilcoxon and auROC calculations. This reduced the running time of the BCC scATAC-seq dataset from 384 min to 1.8 min with similar memory usage (36.9 GB).
- Cell-type annotation function in software that requires training and cross-validation, such as Garnett and SCINA, take significant computational resources. Instead, MAESTRO annotation is based on logFC differential analysis of marker genes, which is more time and memory efficient.
- We reduced the redundancy in Cistrome DB by removing ChIP-seq samples with highly correlated RP distribution for the same TR. This significantly improved the search speed for LISA and GIGGLE in regulator analysis.
- The Snakemake based workflow can allocate all the analysis components in MAESTRO in parallel, which further improves MAESTRO efficiency.

| **MAESTRO Components** | | **RNA** | **ATAC** |
| --- | --- | --- | --- |
| MAESTRO Basic | Barcodes demultiplexing | STARSolo | MAESTRO |
|  | Mapping | STARsolo | Minimap2 |
|  | Bulk level QC | RseQC | MAESTRO |
|  | Single-cell level QC | MAESTRO | MAESTRO |
|  | Quantification | STARsolo | MACS2, MASESTRO |
| MAESTRO Analysis | Gene activity | NA | MAESTRO |
|  | Dimension reduction | PCA | LSI |
|  | Clustering | Seurat | Seurat |
|  | Differential analysis | Presto | Presto |
|  | Cell type annotation | MAESTRO | MAESTRO |
|  | Regulator identification | LISA | GIGGLE |
|  | Integration | Seurat | |

Table R2. Components of MAESTRO that achieved using the existing software. Red indicates the component that has been updated compared to the previous version of MAESTRO.

*6. For the “Multiple levels of quality control” part, this whole section including Figure 2 (Pre-processing and quality control using MAESTRO) are something more like supplementary material.*

We reduced the length of “quality control” section, and move the descriptions of parameters to Methods section in the revised manuscript. However, the other reviewer also mentioned that the description of each QC parameter and the normal range of QC statistics are crucial for users to understand the quality of their samples. Therefore, we added a detailed description of QC parameters in the command-line interface, HTML output, as well as online tutorials of MAESTRO.

*7. For the “Clustering analysis” part, the Figure 3 (Benchmark of scATAC clustering performance on public datasets) have not revealed any new information compared to a recently published work (Chen et al. 2019). Besides, as mentioned at lines 202-204, clustering using candidate cis-regulatory elements (CCRE) catalog from the ENCODE project is not a robust solution. Thus, I suggest the authors move this part to supplementary material.*

We updated the manuscript and move the whole clustering benchmark part to supplementary materials section A.

*Reviewer #2: In this study, the authors developed an integrated software tool MAESTRO for analyzing and integrating single-cell RNA-seq (scRNA-seq) and single-cell ATAC-seq (scATAC-seq) data. This software provides comprehensive functions for read alignment, quality control, clustering, cell type annotation, transcriptional regulator inference and cross-modality alignment and integration. The authors benchmarked some key functions of the software such as scATAC-seq clustering and scRNA-seq and scATAC-seq integration, and they also demonstrated the software using an analysis of bone marrow microenvironment in healthy donors and CLL patients. As there is increasing demands for single-cell transcriptome and regulome analysis, the development of MAESTRO is both timely and useful. The comprehensiveness of the analysis functions and the various benchmark analyses are the strengths of MAESTRO and this manuscript.* 

We thank the reviewer for appreciating the comprehensive functions of MAESTRO and the potential value it will bring to the research community. We are using MAESTRO extensively to analyze large scale single-cell datasets from cancer microenvironment, patient-derived xenografts, hematopoietic differentiation and brain development. Based on the reviewer’s suggestions, we improved the MAESTRO workflow, simplified installation, accelerated the alignment, preprocessing and analysis functions, and added detailed configuration for QC parameters.

*Major issues:

1. While the comprehensive set of functions is a strength of MAESTRO, currently the software still has a lot of external dependencies. In order to use MAESTRO, one needs to install a number of other software tools including Miniconda3, Cell Ranger, RABIT/LISA and giggle in addition to the basic system requirements (Linux/Unix, Python, R). This is a lot of work for users. Depending on users' system and experience, the installation is not necessarily trivial. MAESTRO will be more useful if the authors can simplify the installation procedure.*

We significantly updated the MAESTRO workflow, summarized as follows.

- We changed the default alignment method from BWA-based Cellranger and Cellranger-ATAC pipeline to STARsolo for scRNA-seq and minimap2 for scATAC-seq. This change can make the total alignment and pre-processing speed 15 times faster according to our benchmark (30h to 2h, 8 cores). Besides, different from Cellranger suites, both STARsolo and minimap2 can be automatically installed through conda, thus we will have a simpler installation. On the other hand, if users have Cellranger and Cellranger-ATAC output, the MAESTRO R functions can still be used for all the downstream analyses.
- We set the default method for transcription factor prediction in scRNA-seq clusters to LISA. Besides, we developed an API function in LISA which can let MAESTRO automatically submit the cluster-specific genes from scRNA-seq to the LISA web server (<http://lisa.cistrome.org/>) and retrieve the parsed result. Users do not need to install LISA locally. In addition, we included giggle into our MAESTRO package so it doesn’t have to be installed separately.

With those updates, users only need to install the Miniconda3 environment, then all of the MAESTRO components and dependencies can be installed under this environment with a single command. The detailed updated components of MAESTRO are summarized in Table R2.

*2. Currently MAESTRO only supports Linux/Unix. It could have higher impact if the software can also be run on Windows operating system.*

The MAESTRO workflow contains two major modules, MAESTRO basic and MAESTRO analysis. MAESTRO basic module includes functions for mapping the scRNA-seq and scATAC-seq dataset, pre-processing the barcodes, quantifying the gene expressing and peak calls. As several of the MAESTRO dependent software, such as STARsolo and even Cell Ranger, do not support the windows system, the current MAESTRO basic module can only be used in Linux/Unix system. The MAESTRO analysis module includes the functions for gene accessibility scoring, clustering, differential analyses, cell type annotation, TF inference and integration between scRNA-seq and scATAC-seq. We packaged the analyses module into MAESTRO R package, and this module can be used on both Linux/Unix and Windows operating systems.

*3. For bulk-level quality control, MAESTRO generates a number of QC statistics and figures such as those in Figure 2. However, in practice which cutoff should one use for each QC statistic to decide if a sample is low quality or not?*

The normal ranges for the bulk QC statistics, such as the mapping ratio, the exon/intron reads distribution for scRNA-seq, the mitochondria read ratio for scATAC-seq, can vary across different species and platforms (Manno et al., 2018). We provided the normal ranges of QC statics for human and mouse, and for commonly used single-cell platforms such as 10X Genomics, Smartseq2, Drop-seq and dsci-atac-seq in the online tutorials and the HTML output of MAESTRO.

*4. For single-cell level quality control, the authors described which cutoffs they used for analyzing data in this manuscript and mentioned that these QC parameters are configurable and can be tuned. It would be useful if they can be more concrete and tell potential users how to tune and choose the QC cutoffs for users' own data. It might be useful to provide information on the normal ranges of QC statistics.*

In the updated MAESTRO, we added command-line arguments for the MAESTRO basic module. After loading MAESTRO environment, users can type “MAESTRO scrna-qc -h” or “MAESTRO scatac-qc -h” to see the detailed description of each parameter and the default QC cutoffs. They can tune the QC parameters directly through command line input. Similar to the bulk-level QC statistics, the normal range for single-cell level QC statistics might also vary across different species and platforms. In the MAESTRO online tutorials for 10X Genomics PBMC scRNA-seq and scATAC-seq dataset analyses, we provided the information of the normal ranges of QC statistics and instruction on how to choose the proper cutoff, and discussed QC for other platforms.

*5. For scATAC-seq QC, Figure 2f shows that the high-quality and low-quality cells form distinct clusters when plotted using "Reads passed filters" and "Fraction of reads in promoter". This plot is used to demonstrate MAESTRO's ability to select high-quality cells. However, is this "clear separation" between high- and low-quality cells always true or is it only specific to this example dataset? In order to show that this "clear separation" is generally useful for QC, it is important to show that it is a general pattern in multiple independent datasets.*

MAESTRO separates high-quality and low-quality cells based on the number of reads passed filters and the fraction of reads in promoter regions. For all the 10X Genomics datasets, the high-quality and low-quality cells form distinct clusters based on our observations, reflecting the distinct amount of DNA content or sequenced reads between empty droplets and droplets with cells (Figure R7). We did not observe a clear separation for scATAC-seq datasets using microfluidics-based devices, possibly because such devices usually load fewer cells (i.e. lower throughput) and have a relatively higher capture efficiency compared to droplet-based methods.

Figure R7. Visualization of high-quality and low-quality cells from scATAC-seq selected by MAESTRO.

(a) Datasets from 10X Genomics based platform.

(b) Datasets from the microfluidics-based platform. *6. For scATAC-seq clustering analysis, chromVAR was not benchmarked in the manuscript but it is incorporated into MAESTRO. In contrast, snapATAC-seq was benchmarked but it is not incorporated into MAESTRO. Can authors explain why?*

ChromVAR calculates the accessibility derivations for each transcription factor at single-cell resolution. It was reported to have worse performance in clustering scATAC-seq data compared to LSI-based method, cisTopic and snapATAC (Chen et, al. 2019). On the other hand, chromVAR provides information on TF motif enrichment in each cell, which is complementary to TF ChIP-seq information in Cistrome database. In the revised manuscript, we clarified that 1. the default dimension reduction and clustering method for scATAC-seq dataset is LSI-based method, with the LDA-based method implemented in cisTopic as an alternative option; 2. the default TF inference method is giggle, and chromVAR is an alternative option if users want to investigate the motif enrichment.

*7. The most difficult part for automatic cell type annotation actually is compiling marker genes for each cell type. Currently, MAESTRO only provides marker genes for immune cell types. It will be more useful if marker genes for other major cell types could also be provided as a reference database.*

We included more cell-type signatures in MAESTRO such as signatures from cancer microenvironment, adult mouse brain (Tasic et al., 2016), as well as signatures from cell atlas project such as Tabula Muris. Besides, MAESTRO also supports reading cell-type signatures from Cell Marker database (<http://biocc.hrbmu.edu.cn/CellMarker/download.jsp>) and PanglaoDB (<https://panglaodb.se/markers.html?cell_type=%27choose%27>). Users could download the cell-type specific marker genes from these databases or even compile their own cell-type signature gene list and use it as a reference for MAESTRO.  *8. For integrating scRNA-seq and scATAC-seq, the authors mentioned that the other three methods (snapATAC, cicero, Seurat) failed to align cells for rare cell populations such as pDCs or Mast cells (Figure S7a-f). However, they did not mention how MAESTRO performed in those rare cell populations. Indeed, Figure 5a-b seem to suggest that MAESTRO also failed to align Mast cells. Moreover, interpretation of Figure S7a-f and Figure 5a-b using human eyes could be misleading. For example, if blue data points are plotted after plotting the red data points, it is possible that the red data points in certain regions of the UMAP plot are all covered by blue data point. As a result, one cannot see any red dots by eye in these regions (e.g., pDC, Mast) even though both blue and red data points exist and mix/align well. Thus, it is important to "quantify" the overlap between scRNA-seq and scATAC-seq in order to convincingly compare different methods' ability to align cells for rare cell populations.*

For each cell type in the PBMC Different Donors dataset, we quantified the number of cells that successfully received transferred labels from scRNA-seq to scATAC-seq (Table R1 and Table S5 in Additional File 7). For all the three tested dataset, MAESTRO, Seurat and snapATAC shows better performance than cicero, while for PBMC Different Donors and BMMC same Donor dataset, MAESTRO have the lowest cumulative error among all the methods. For PBMC Different Donors dataset, cicero failed to align Memory B-cells, CD 16 Monocytes and DC cells, and snapATAC failed to align pDC cells. All of the tested methods failed to align CD4 Naïve T-cells, Macrophage and Mast cells, while the number of CD14 Monocytes is much higher than expected. These analysis indicated the chromatin landscape of Macrophage/Mast cells might resemble other innate immune cells such as Monocytes thus cannot be correctly assigned using any current strategies. We included the detailed tables for the three datasets and discussed the above analysis in the revised manuscript. More details can be found in our response to reviewer #1’s point #2.

*9. In Figure 5e and Figure S8d, the improvement of MAESTRO over Seurat and snapATAC in terms of NMI seems to be small. However, in Figure 5f and Figure S8c, the improvement of MAESTRO over Seurat and snapATAC in terms of correlation between ATAC and RNA gene expression seems to be much bigger. It is unclear how much of the improvement in Figure 5f and Figure S8c is attributed to the improved cell alignment (which is what NMI tries to measure) and how much of the improvement is simply caused by the improved correlation between the regulatory potential (RP) and gene expression conditional on fixing cell alignment. It will be useful to decompose Figure 5f (and similarly Figure S8c) into two figures. In the first figure, scRNA-seq and scATAC-seq are aligned using different methods (MAESTRO, Seurat, snapATAC, cicero), and for each alignment method the correlation between ATAC and RNA is computed using the RP and gene expression (even if the RP is not used in aligning the cells for Seurat, snapATAC and cicero). These four correlations are then compared. This figure will show the pure gain of MAESTRO over the other methods in terms of cell alignment. In the second figure, using the cell alignment provided by MAESTRO, compute and compare the correlation between ATAC and RNA using four different ways to summarize ATAC data (RP by MAESTRO, promoter and gene-body accessibility score from Seurat, gene-body accessibility score from snapATAC, and co-accessibility gene score from cicero). This figure will show the pure effect of using RP in improving the correlation between the RNA-seq gene expression measurements and ATAC-seq chromatin accessibility measurements.*

We thank the reviewer for this helpful suggestion! In the revised manuscript, we added additional analyses on multiple datasets to demonstrate the superior performance of MAESTRO. We generated the distribution of label prediction scores after integrating scATAC-seq with scRNA-seq. We found the MAESTRO regulatory potential (Regulation Potential) model has the overall highest cell type prediction score (Figure R3). After filtering cells with lower prediction scores, MAESTRO could successfully align more cells than Seurat, snapATAC and cicero (Figure R2). In addition, we evaluated the correlation between gene expression levels and gene activity scores of all genes or only the expressed genes (filtered by log2 UMI from scRNA-seq). Both results suggest that MAESTRO RP has higher consistency (Pearson’s correlation) with scRNA-seq. Furthermore, while only considering the expressed genes, MAESTRO RP score is the only one positively correlated with gene expression (Figure R2). Taken together, the above analyses suggest that MAESTRO has better performance to align the cells and predict the expression level from chromatin accessibility. We updated the figures and manuscript accordingly. More details can be found in our response to reviewer #1’s point #2.

*Minor issues:

10. While MAESTRO is an abbreviation for Model-based AnalysEs of Transcriptome and RegulOme, it is unclear which part of the software represents "Model-based". Most functions of the software are implemented by calling other existing tools. This is fine from the perspective of developing useful software. However, for naming the software, are there any new "models" developed here that is unique for MAESTRO? If there are not, using the word "model-based" is somewhat confusing and misleading.*

The “model-based” in MAESTRO represents the regulatory potential model for estimating the gene activity score from scATAC-seq, which is the key step to annotate the scATAC-seq clusters and to integrate scATAC-seq with scRNA-seq. We addressed the logistic of RP definition, the half-decay distance estimation in our response to point #1 from reviewer #1 and the revised supplementary materials. We also reorganized the manuscript to highlight the regulatory potential model in MAESTRO.

*11. Related to the above comment, Table 1 is very useful for comparing functions of MAESTRO with other methods. It would be also useful to annotate which of the functions in MAESTRO are based on calling existing methods and software tools (e.g. alignment, etc.), and which of the functions are newly developed in MAESTRO and therefore represent new methods. This will be helpful for highlighting the methodological novelties of MAESTRO.*

We thank the reviewer for this helpful suggestion! In the revised manuscript, we added a table to summarize all the integrated software as well as newly developed components in MAESTRO (Table R2 and Table S2 in the Additional file 4). *12. How many healthy donors and CLL patients are used in the CLL example? The description in the method seems to suggest that there is only one healthy donor and one patient. However, throughout the paper, the authors used the plural form "healthy donors" and "patients" instead of "healthy donor" and "patient".*

Only one healthy donor and one CLL patient are used in the CLL example. We corrected the descriptions to “a healthy donor” and “a patient”.

*13. Line 569 "marker genes divided by log length of marker genes": here the meaning of "length" is unclear. Does it refer to the length of gene body (in the unit of base pairs) or do you mean the total number of marker genes?*

The “length” means the total number of marker genes. We edited the description as “divided by log2 total number of marker genes for that cell-type”. *14. Figure S8: the text description for S8(c) and S8(d) should be switched. Also, for S8(b), the dot size represents the fraction of cells that have a "higher" regulatory potential level. How is "higher" defined? What is the cutoff?*

We switched the order of figures in the revised manuscript. The dot size represents the fraction of cells that have non-negative log2 RP score for each gene shown in the figure. We changed the description from “higher RP” to “non-negative log2 RP” in all the related text. *15. There are a number of typos in the manuscript. For example
1) Line 497: "Principle Component Analysis" should be "Principal Component Analysis".
2) Line 539: "minimum presence faction" should be "minimum presence fraction".
3) Line 569: "devided" should be "divided". 
4) Figure 1: in the Analysis box of the figure, "Exprssion Index" should be "Expression Index".*

We corrected all the above typos in the revised manuscript.

**Second round of review**

**Reviewer 1**

The authors have significantly improved the quality of the manuscript. They have reorganized the manuscript, had a better explanation of the regulatory potential model, and conducted comparisons to the existing software on more independent datasets. Furthermore, they optimized the performance of MAESTRO by significantly simplifying the installation, accelerating multiple steps, and improved the usability by adding detailed descriptions and documentation. The authors have tried very hard to demonstrate that the regulatory potential score has better performance in integration. However, this conclusion is still not supported well by the analysis.

Figure R2 (a) (b) show that, if the correlation is calculated by Spearman's correlation coefficients, SnapATAC will show comparable or even better performance compared to MAESTRO. This fact indicates that all the conclusions based on Pearson's correlation coefficients maybe not solid. Figure R2 (c)(d)(e) shows that regulatory potential score is the only one that can keep a positive correlation for "expressed genes" regardless of the way of correlation calculation. However, this impression is inconsistent with the fact that other methods also have good performance for transferring labels from RNA to ATAC (Supplementary Table 5, Table R1). Instead of "all genes" or "expressed genes", it will make more sense to perform the same analysis as in Figure R2 (c-e) for "highly variable genes", since only those genes are used in both clustering of scRNA-seq and integration of scRNA-seq and scATAC-seq. If regulatory potential score indeed has a higher correlation than other gene activity scores for "highly variable genes", then the authors can conclude that regulatory potential score is a better indicator for gene expression. And this result will be worth being presented in the main figures.

Base on Supplementary Table 5(Table R1), MAESTRO has slightly better performance at the single-cell level in terms of "Cumulative error". However, at the cluster level, the success rate of transferring of MAESTRO in Dataset 3 is 11/12. It is even worse compared to Seurat and SnapATAC because the DC cells are almost missing in MAESTRO's results. And in Dataset 1 and Dataset 2, the success rates of MAESTRO are 10/13 and 9/9 respectively, which are the same as Seurat. Overall, it is clear that MAESTRO outperforms Cicero. However, replacing the original gene activity score in Seurat or SnapATAC by regulatory potential score provide limited benefit for integration. Thus, I suggest the author do a more in-depth analysis to help the users to understand why the failure of transferring at cluster level happens. There could be many reasons. a) the population that exists in RNA-seq may be lost in the ATAC experiment. For example, the authors could identify Macrophage/Mast's marker genes in the scRNA-seq of Dataset 1 and plot those genes' regulatory potential scores in scATAC-seq data. If the population is not existing in scATAC-seq, then indeed there are no ways to transfer the label. b) The cluster is a lack of positive marker genes. For example, a subpopulation of T cells may be separated from other subtypes by a limited number of negative markers while without positive marker genes. If this is the case, the potential solution could be merge subtypes together before transferring. After transferring, further, separate them based on subtype-specific gene's regulatory potential score. c) MAESTRO failed to construct a regulatory potential score for marker genes. Since the regulatory potential score is peak based, the cell-type-specific peaks may not be included in the peaks called from aggregated scATAC-seq. The potential solution could be switching to a bin-based gene activity calculation. d) CCA failed to find anchors for a particular cluster. The original CCA solution in Seurat didn't print out any intermediate files. It will be very helpful if the author could modify the code to print out intermediate files and generate some tables or figures to summary the anchor's information for each label. e) Seurat failed to get a high prediction score for a particular cell because of the small population size. During anchor weighting step, if the cell number of the cluster is small, the default parameter maybe not optimized for a cell to receive a high prediction score from a small number of anchors. If the MAESTRO workflow can provide a serious of "diagnosing" functions to tell the different possibilities mentioned above and functions for improving cluster level label transferring, it will make a unique contribution for integration analysis and sufficient to be published. I suggest that the author does not make a strong conclusion about the performance of the regulatory potential score. Instead, the author can emphasis that "MAESTRO is super easy to install, friendly for parallel computing, includes plenty of diagnosing functions and is flexible to construct a customized pipeline to achieve higher performance".

Besides, there are some minor issues need to be further addressed.

In the "Clustering evaluation based on the simulated scATAC-seq dataset" section, the authors have evaluated the effect of different sequencing depth on scATAC-seq Clustering. However, how the sequencing depth of scATAC-seq will affect the performance of the integration of MAESTRO, Seurat, SnapATAC has not been evaluated. For the datasets used in Figure 6, if downsampling the data to 80%, 60%, 40%, and 20% of the original data, the proportion of high prediction score, Pearson's correlation between RNA and ATAC will be expected to decrease. However, the results of which method, MAESTRO, Seurat, snapATAC, or cicero will decrease slower will be important. In other words, how is MAESTRO tolerated to low depth data, compared to other methods?

Supplementary Table 2 shows that for Dataset 2 BCC scATAC-seq MAESTRO needs ~50G memory. However, in the response letter, the authors mentioned that "We optimized the regulatory potential calculation in MAESTRO by implementing the matrix manipulation function in Python, which can handle scATAC-seq with 38K cells and ~500K peaks on a computer with 380GB memory". The inconsistency needs to be clarified.

Supplementary Table 5(Table R1) shows that for CD4 Naïve T cell, all methods failed to transfer labels from RNA to ATAC. However, Figure 4 d,e, and Figure R6 show there are some data for CD4 Naïve T cell in scATAC-seq data. The inconsistency needs to be clarified.

Line 476-479 and Figure 4 (a)(b) mentioned that "All of the tested methods fail to align Macrophage and Mast cells, indicating that the chromatin landscape of Macrophage/Mast cells might resemble other innate immune cells such as Monocytes thus cannot be correctly assigned using any current strategy". This conflicts with the fact that in Dataset 3, Mast cells have been success transferred together with Mono cells.

**Reviewer 2**

The authors have satisfactorily addressed my questions.

**Authors Response**

**Point-by-point responses to the reviewers’ comments:**

We thank the reviewer’s suggestions on how to improve MAESTRO. Based on the reviewer’s comments, we have updated the regulatory potential model of MAESTRO, performed more comprehensive analyses, and added several novel functions in the MAESTRO workflow. The major changes are summarized below.

- We have modified the regulatory potential model of MAESTRO by removing the interfering peaks from adjacent genes and adjusting the weight of exon peaks. We demonstrated that the new model significantly improved the consistency between gene expression and gene activity from the same cluster and outperforms the existing methods for integrative analysis.
- We have performed down-sampling analysis and showed that MAESTRO is robust to datasets with low sequencing depth.
- We have modified the integration function of MAESTRO, and output more intermediate figures and log file for diagnosing the possible failure in integrating rare cell populations.
- Besides the suggested function from the reviewer, we have also added functions for 1) automatically annotating cell-types for scATAC-seq clusters based on public bulk chromatin accessibility dataset from Cistrome Database; 2) generating genome browser tracks at cluster level for scATAC-seq dataset visualization; 3) supporting peak calling and downstream analysis by creating pseudo bulk samples for scATAC-seq clusters.

We provided a point-by-point response as follows. The reviewer’s comments are in *blue italics* followed by our responses in regular font.

*Reviewer #1: The authors have significantly improved the quality of the manuscript. They have reorganized the manuscript, had a better explanation of the regulatory potential model, and conducted comparisons to the existing software on more independent datasets. Furthermore, they optimized the performance of MAESTRO by significantly simplifying the installation, accelerating multiple steps, and improved the usability by adding detailed descriptions and documentation. The authors have tried very hard to demonstrate that the regulatory potential score has better performance in integration. However, this conclusion is still not supported well by the analysis.* 

We thank the reviewer for the thoughtful suggestions on how to improve the integrating function of MAESTRO. In the original MAESTRO regulatory potential (RP) model, scATAC-seq peaks surrounding each gene were considered as potential transcriptional regulator binding sites that impact gene expression, and the contribution of each peak to gene expression was modeled by a monotonically decaying function by the distance to TSS. However, in scATAC-seq datasets, the exon region of a gene can also be accessible, which might reflect RNA Pol II transcriptional complex on chromatin during transcription. In addition, we noticed that unexpressed genes may be incorrectly assigned with gene regulatory potential scores due to the strong promoter and exon signals from nearby expressed genes. Based on these considerations, we have updated our RP model to evaluate the impact of nearby chromatin accessibility on gene expression. In the new RP model, MAESTRO gives the peaks present in the exon region of the candidate gene an equal weight as the TSS (weighted as 1 and normalized by the exon length, Gene A in Figure R1a), and removes the peaks located in nearby promoters and exons (Gene B in Figure R1a). We have tested the performance on integrating scATAC-seq with scRNA-seq of the old RP model (termed as “simple RP model”, model 1, the red group in Figure R1 b and c), the new RP model (termed as “enhanced RP model”, model 8, the pink group in Figure R1 b and c), and several other model combinations. Our analysis showed that after integration, the “enhanced RP model” has the highest label prediction score compared to other models (pink group versus all other groups in Figure R1b), which indicates the cell-type labels from scRNA-seq were transferred more appropriately. In addition, we have compared the consistency of gene activity with gene expression for each cell-type cluster, and the “enhanced RP model” has an overall higher consistency in the Spearman’s correlation coefficient on three different datasets (Figure R1c). These results suggest that the “enhanced RP model” has improved performance in both integrating scATAC-seq with scRNA-seq, and predicting potential gene expression from scATAC-seq. We thus set the “enhanced RP model” as the default gene scoring model for MAESTRO, but also support the “simple RP model” as alternative options. We have added these new results in the revised Supplementary Materials.

Figure R1. Comparison of scRNA-seq and scATAC-seq integration performance using different regulatory potential models

(a) Schema of the new regulatory potential model in MAESTRO. For each gene, we weighed the presence of ATAC-seq peaks around its TSS by an exponential decay function on its distance to TSS, with 10kb as half-decay distance to both directions. Peaks present within the exon region of the candidate gene (Gene A) were given a weight of 1 and further normalized by exon length. Peaks located in the nearby gene promoter or exon regions (Gene B) were removed from the score calculation. The weighted scores for each peak (green bar in the figure) are summed to generate the regulatory potential score for the candidate gene.

(b) Distribution of cell-type label transferring score for different RP models, listed as follows. The 8^th^ model is the default RP model used in MAESTRO.

1: TSS-centered RP, simple RP model;

2: Gene-centered RP;

3: TSS-centered RP excluding peaks in adjacent gene promoters;

4: TSS-centered RP excluding peaks in adjacent gene promoters and exons;

5: TSS-centered RP excluding peaks in adjacent gene promoters and gene-bodies;

6: TSS-centered RP with equal weights for peaks located in exon of candidate genes, normalized by total exon length;

7: TSS-centered RP with equal weights for peaks located in gene-body of candidate genes, normalized by gene body;

8: TSS-centered RP, with equal weights for peaks located in exon of candidate gene, normalized by exon length , excluding peaks in any other gene promoters and exons, enhanced RP model;

9: TSS-centered RP, with equal weights for peaks located in gene-bodies of candidate gene, excluding peaks in any other gene promoters and gene-bodies;

(c) Distribution of Spearman’s correlation coefficient between RP score and gene expression for each cluster using different models from Figure R1b.

*Figure R2 (a) (b) show that, if the correlation is calculated by Spearman's correlation coefficients, SnapATAC will show comparable or even better performance compared to MAESTRO. This fact indicates that all the conclusions based on Pearson's correlation coefficients maybe not solid. Figure R2 (c)(d)(e) shows that regulatory potential score is the only one that can keep a positive correlation for "expressed genes" regardless of the way of correlation calculation. However, this impression is inconsistent with the fact that other methods also have good performance for transferring labels from RNA to ATAC (Supplementary Table 5, Table R1). Instead of "all genes" or "expressed genes", it will make more sense to perform the same analysis as in Figure R2 (c-e) for "highly variable genes", since only those genes are used in both clustering of scRNA-seq and integration of scRNA-seq and scATAC-seq. If regulatory potential score indeed has a higher correlation than other gene activity scores for "highly variable genes", then the authors can conclude that regulatory potential score is a better indicator for gene expression. And this result will be worth being presented in the main figures.
Base on Supplementary Table 5(Table R1), MAESTRO has slightly better performance at the single-cell level in terms of "Cumulative error". However, at the cluster level, the success rate of transferring of MAESTRO in Dataset 3 is 11/12. It is even worse compared to Seurat and SnapATAC because the DC cells are almost missing in MAESTRO's results. And in Dataset 1 and Dataset 2, the success rates of MAESTRO are 10/13 and 9/9 respectively, which are the same as Seurat. Overall, it is clear that MAESTRO outperforms Cicero. However, replacing the original gene activity score in Seurat or SnapATAC by regulatory potential score provide limited benefit for integration.*

With the updated regulatory potential model, we have compared the performance of integration using MAESTRO, Seurat, snapATAC, and cicero. In our previous analysis, we compared the cell-type composition of scATAC-seq and scRNA-seq dataset, and evaluated the consistency using normalized mutual information (NMI). However, the cell-type composition of scATAC-seq and scRNA-seq were not necessarily exactly the same, especially for Dataset 1, in which the scATAC-seq and scRNA-seq PBMC samples were actually derived from different healthy donors. For this reason, we removed the consistency analysis of cell-type proportions as well as the cumulated error analysis in Supplementary Table 5, and only used the distribution of cell-type label transferring score and the number of cells that has a robust prediction (cell-type label prediction score > 0.5) to evaluate the integration performance (Figure R2a and Table R1). We found that the “enhanced RP model” of MAESTRO gave a significantly higher cell-type label transferring score compared to the other three methods for the PBMC dataset from different donors (Dataset 1) and the BMMC dataset from the same donor (Dataset 3). MAESTRO has similar performance with Seurat for the PBMC dataset from the same donor (Dataset 2, Figure R2a). Consistently, MAESTRO has a higher number of successfully transferred cells for Dataset 1 and Dataset 3, and only lower than Seurat for Dataset 2 (Table R1). Integration using the “enhanced RP model” also successfully transferred the DC labels for Dataset 3, which was failed in the original RP model (Table R1). These analyses collectively suggest that the RP model used by MAESTRO has comparable if not better performance in integrating scATAC-seq with scRNA-seq.

We have also demonstrated that the RP score from MAESTRO has a higher consistency with gene expression, no matter whether the consistency was evaluated by using all the genes, or the top 2000 highly variable genes as suggested by the reviewer (Figure R2c, d, evaluated using spearman’s correlation coefficient). This supports the RP score in MAESTRO as a better proxy of gene expression than other methods. We have updated the regulatory potential model and the corresponding analyses in the revised manuscript.

Figure R2. Comparison of scRNA-seq and scATAC-seq integration performance of MAESTRO, Seurat, snapATAC and cicero

(a) Distribution of cell-type label transferring score for MAESTRO, Seurat, snapATAC and cicero on three independent datasets. Statistical significance was evaluated using two-sided Wilcoxon rank-sum test.

(b) Distribution of Spearman’s correlation coefficient between gene activity score and gene expression using all genes for each cluster. Colors represent different methods.

(c) Distribution of spearman’s correlation coefficient between gene activity score and gene expression using top 2000 highly variable genes for each cluster. Colors represent different methods.

**Dataset 1: PBMC Different Donors**

| Methods | B Naive | B Memory | CD14 Mono | CD16 Mono | CD4  Naive T | CD4 Memory T | CD8  Naïve T | CD8 Activated T | NK | DC | pDC | Macrophage | Mast | Total |
| --- | --- | --- | --- | --- | --- | --- | --- | --- | --- | --- | --- | --- | --- | --- |
| MAESTRO | 385 | 218 | 3759 | 249 | 0 | 1552 | 399 | 1231 | 471 | 193 | 64 | 0 | 0 | 8521 |
| Seurat | 348 | 238 | 3842 | 229 | 0 | 1541 | 410 | 1230 | 467 | 101 | 63 | 0 | 0 | 8469 |
| snapATAC | 331 | 236 | 3738 | 220 | 0 | 1516 | 391 | 1251 | 459 | 135 | 0 | 0 | 0 | 8277 |
| cicero | 561 | 22 | 4339 | 6 | 0 | 1431 | 398 | 1128 | 459 | 3 | 62 | 0 | 0 | 8409 |

**Dataset 2: PBMC Same Donor**

| Methods | B Naive | CD14 Mono | CD16 Mono | CD4 Naive T | CD4 Memory T | CD8 T | NK | DC | pDC | Total |
| --- | --- | --- | --- | --- | --- | --- | --- | --- | --- | --- |
| MAESTRO | 769 | 3264 | 349 | 1775 | 743 | 1576 | 575 | 176 | 71 | 9298 |
| Seurat | 769 | 3446 | 358 | 1665 | 937 | 1595 | 581 | 237 | 70 | 9658 |
| snapATAC | 750 | 3061 | 414 | 1539 | 832 | 1557 | 579 | 178 | 71 | 8981 |
| cicero | 767 | 4260 | 0 | 1868 | 0 | 675 | 531 | 0 | 0 | 8101 |

**Dataset 3: BMMC Same Donor**

| Methods | CD4 T | CD8 Naïve T | CD8 Activated T | NK | CLL1 | CLL2 | Early B | Pre-pro B | DC | Mast | MastPro | Mono | Total |
| --- | --- | --- | --- | --- | --- | --- | --- | --- | --- | --- | --- | --- | --- |
| MAESTRO | 1672 | 695 | 51 | 615 | 2034 | 278 | 839 | 157 | 67 | 115 | 229 | 781 | 7533 |
| Seurat | 1554 | 409 | 193 | 516 | 1953 | 338 | 343 | 145 | 72 | 113 | 210 | 739 | 6585 |
| snapATAC | 1525 | 857 | 30 | 320 | 2354 | 166 | 632 | 152 | 62 | 110 | 178 | 690 | 7076 |
| cicero | 1583 | 478 | 84 | 340 | 2283 | 29 | 458 | 0 | 51 | 7 | 223 | 813 | 6349 |

Table R1. The number of cells in scATAC-seq that successfully received transferred labels from scRNA-seq for each cell type (prediction score > 0.5). The last column represents the total number of cells that have a high prediction score.

*Thus, I suggest the author do a more in-depth analysis to help the users to understand why the failure of transferring at cluster level happens. There could be many reasons. a) the population that exists in RNA-seq may be lost in the ATAC experiment. For example, the authors could identify Macrophage/Mast's marker genes in the scRNA-seq of Dataset 1 and plot those genes' regulatory potential scores in scATAC-seq data. If the population is not existing in scATAC-seq, then indeed there are no ways to transfer the label. b) The cluster is a lack of positive marker genes. For example, a subpopulation of T cells may be separated from other subtypes by a limited number of negative markers while without positive marker genes. If this is the case, the potential solution could be merge subtypes together before transferring. After transferring, further, separate them based on subtype-specific gene's regulatory potential score. c) MAESTRO failed to construct a regulatory potential score for marker genes. Since the regulatory potential score is peak based, the cell-type-specific peaks may not be included in the peaks called from aggregated scATAC-seq. The potential solution could be switching to a bin-based gene activity calculation. d) CCA failed to find anchors for a particular cluster. The original CCA solution in Seurat didn't print out any intermediate files. It will be very helpful if the author could modify the code to print out intermediate files and generate some tables or figures to summary the anchor's information for each label. e) Seurat failed to get a high prediction score for a particular cell because of the small population size. During anchor weighting step, if the cell number of the cluster is small, the default parameter maybe not optimized for a cell to receive a high prediction score from a small number of anchors. If the MAESTRO workflow can provide a serious of "diagnosing" functions to tell the different possibilities mentioned above and functions for improving cluster level label transferring, it will make a unique contribution for integration analysis and sufficient to be published. I suggest that the author does not make a strong conclusion about the performance of the regulatory potential score. Instead, the author can emphasis that "MAESTRO is super easy to install, friendly for parallel computing, includes plenty of diagnosing functions and is flexible to construct a customized pipeline to achieve higher performance".* 

We investigated the possible explanations for the failure in integrating Macrophage and Mast population for Dataset 1. As mentioned earlier, for this dataset, the scATAC-seq and scRNA-seq samples were generated from different donors, thus Macrophage and Mast cells might not be present in the scATAC-seq sample. We checked the positive markers PPBP and GNG11 for Macrophage, and TPSAB1 for Mast cells. The expression of those markers can be observed from scRNA-seq dataset, but their gene activity are missing in almost all the cells from scATAC-seq dataset, whether evaluating using RP or other gene scoring method, indicating these two populations might not be existing in scATAC-seq samples (Figure R3a, b). Besides, no anchor cell pairs were identified for Mast cell and only 1 pair was identified for Macrophage using Seurat CCA analysis (Figure R3c). These analyses suggested that Macrophage and Mast cells might be under-represented in the scATAC-seq samples, thus all of the methods, including MAESTRO, Seurat, snapATAC and cicero, failed to integrate them regardless of the gene activity scoring methods. We have added the analysis in the revised manuscript. Following the reviewer’s suggestion, we implemented functions to provide diagnosing figures and logs during the integration process for users to investigate the possible reasons of failure when integrating rare cell populations.

Figure R3. Mast and Macrophage population are not presented in PBMC Different Donors scATAC-seq dataset

(a) Umap plot showing the gene expression from scRNA-seq and gene activity from scATAC-seq for Macrophage markers PPBP and GNG11.

(b) Umap plot showing the gene expression from scRNA-seq and gene activity from scATAC-seq for Mast marker TPSAB1.

(c) Number of anchor cell pairs identified using Seurat CCA analysis in different cell types.

*Besides, there are some minor issues need to be further addressed. 
1.      In the "Clustering evaluation based on the simulated scATAC-seq dataset" section, the authors have evaluated the effect of different sequencing depth on scATAC-seq Clustering. However, how the sequencing depth of scATAC-seq will affect the performance of the integration of MAESTRO, Seurat, SnapATAC has not been evaluated. For the datasets used in Figure 6, if downsampling the data to 80%, 60%, 40%, and 20% of the original data, the proportion of high prediction score, Pearson's correlation between RNA and ATAC will be expected to decrease. However, the results of which method, MAESTRO, Seurat, snapATAC, or cicero will decrease slower will be important. In other words, how is MAESTRO tolerated to low depth data, compared to other methods?* 

We performed the down-sampling analysis following the reviewer’s suggestions. The PBMC scATAC-seq sample from the same donor (Dataset 2) includes 10K high-quality cells and was deeply sequenced with ~770M mappable read pairs, which is about 77K read pairs per cell. We randomly down-sampled the data to 60% (~46K/cell), 40% (~30K/cell), 20% (~15K/cell), 10% (~8K/cell), 5% (~4K/cell) and 1% (~0.8K/cell) of the original data. For each condition, we re-did the peak calling, gene activity modeling, clustering, and integration analysis and compared with the results using the full dataset. We only evaluated the integration performances of MAESTRO, Seurat, and snapATAC as cicero showed poor performance in terms of integration. For integrating with scRNA-seq and label transferring analysis, all of the tested methods showed relatively robust results at the sequencing depth of 15K read pairs per cell. MAESTRO and snapATAC showed slightly better integration performance than Seurat at a lower sequence depth with 8K or even 4K read pairs/cell (Figure R4 a, b). Besides, the peak-based regulatory potential score from MAESTRO was very robust to the sequencing depth, and showed a better consistency with gene expression even at a low sequence depth with 4K read pairs/cell compared to snapATAC and Seurat. In summary, these analyses demonstrated that both MAESTRO and snapATAC are more tolerated to low depth data than Seurat, and the regulatory potential score from MAESTRO is better at predicting gene expression even at a low sequencing depth of 4K read pairs/cell.

Figure R4. Comparison of integration performance of MAESTRO, snapATAC and Seurat using down-sampled scATAC-seq datasets

(a) NMI of the transferred scATAC-seq cell-type labels at different sequencing depth with the transferred scATAC-seq cell-type labels using original dataset.

(b) Distribution of cell-type label transferring score at different sequencing depth.

(c) Consistency between the gene expression from scRNA-seq and gene activity score from scATAC-seq at different sequencing depth. After integration, gene expression and gene activity score from each cell type cluster were averaged by cell and the consistency per cluster was evaluated using spearman’s correlation coefficients.

*2.      Supplementary Table 2 shows that for Dataset 2 BCC scATAC-seq MAESTRO needs ~50G memory. However, in the response letter, the authors mentioned that "We optimized the regulatory potential calculation in MAESTRO by implementing the matrix manipulation function in Python, which can handle scATAC-seq with 38K cells and ~500K peaks on a computer with 380GB memory". The inconsistency needs to be clarified.* 

We thank the reviewer for this comment! The “380GB memory” in the response letter was to describe the total memory of the computer we used for the analysis. When processing the BCC scATAC-seq dataset, MAESTRO only consumed ~50GB memory on that computer. We have clarified this in the revised manuscript.

*3.      Supplementary Table 5(Table R1) shows that for CD4 Naïve T cell, all methods failed to transfer labels from RNA to ATAC. However, Figure 4 d,e, and Figure R6 show there are some data for CD4 Naïve T cell in scATAC-seq data. The inconsistency needs to be clarified.*

We thank the reviewer for pointing this out. MAESTRO indeed transferred CD4 Naïve T-cells labels to 5 cells in the PBMC scATAC-seq dataset from different donors (Dataset 1), however, none of the cells have a prediction score larger than 0.5. In Supplementary Table 5, we only quantified the cells if they have a predicted label transfer score greater than 0.5. We have removed the CD4 Naïve T-cell cluster from Figure 4 d,e for consistency in the revised manuscript.

*4.      Line 476-479 and Figure 4 (a)(b) mentioned that "All of the tested methods fail to align Macrophage and Mast cells, indicating that the chromatin landscape of Macrophage/Mast cells might resemble other innate immune cells such as Monocytes thus cannot be correctly assigned using any current strategy". This conflicts with the fact that in Dataset 3, Mast cells have been success transferred together with Mono cells.*

For the PBMC dataset from different donors (Dataset 1), the scRNA-seq and scATAC-seq samples are actually from different healthy donors, and we have demonstrated that the failure in integration was caused by under-representation of Macrophage and Mast cell populations in scATAC-seq samples (Figure R3). For the BMMC dataset from the same donors (Dataset 3), the scRNA-seq and scATAC-seq data are from the same donors and thus all the cell types were successfully transferred using different methods (Supplementary Table 5). We have modified the description accordingly in the revised manuscript.

**Third round of review**

**Reviewer 1**

The authors have performed a more comprehensive analysis and added some nice new functions to MAESTRO. However, it is a little bit strange that they have changed their default regulatory potential model to a different one, which is not asked by the reviewers. Additionally, the changes make their new “enhanced RP model” very similar to the best model (model 42) proposed by a recently released work named ArchR https://www.biorxiv.org/content/10.1101/2020.04.28.066498v1 from William J. Greenleaf’s lab. Since the regulatory potential model is the key to the integrative analyses of single-cell RNA-seq and ATAC-seq data, I believe it is necessary for the authors to make some clarification carefully.

ArchR has benchmarked 56 different models for inferring gene expression from scATAC-seq data using matched scATAC-seq and scRNA-seq data. And here is the description of their best model, “The most accurate model across both datasets was Model 42 (a model within the gene body extended + exponential decay + gene boundary class of models) (Fig. 2b) which integrates the signal from the entire gene body, and scales signal with bi-directional exponential decays from the gene TSS (extended upstream by 5 kb) and the gene transcription termination site (TTS) while accounting for neighboring genes boundaries (Fig. 2c). This model yielded robust genome-wide gene score predictions in both PBMC and bone marrow cell datasets (Fig. 2d-f and Supplementary Fig. 9i- j). We additionally confirmed the efficacy of this class of gene score models using previously 8 published paired bulk ATAC-seq and RNA-seq data from hematopoietic cells (Supplementary Fig. 9k-m)19. Given this analysis, we implemented this class of gene score models via Model 42 for all downstream analyses involving inferred gene expression in ArchR.”
The major difference between the MAESTRO ‘s “enhanced RP model” and ArchR’s Model 42 is that the “enhanced RP model” only has high weight for exon peaks. However, this is also suspicious. To my knowledge, there is no single literature has mentioned that ATAC peaks are enriched in exon regions. If the “enhanced RP model” also give high weight for intron peaks, then it will be almost the same as ArchR’s Model 42.

I believe the regulatory potential model, referred to as the “simple RP model” in the R2 version manuscript, is an original idea from MAESTRO. However, actually, it is very similar to Model 21 mentioned by ArchR and it has been shown to has poor performance compared to Model 42 in ArchR’s systematic comparative analysis. On the other hand, it is hard to tell whether the new “enhanced RP model” is “partially” inspired by ArchR or independently discovered by MAESTRO’s authors. Since the ArchR is only online in bioRxiv and has not been officially published, it is maybe not reasonable to ask the authors to include ArchR to do more comparative analysis. But it will be very important for the authors to cite ArchR’s bioRxiv manuscript in the main text and discuss what is the relationship between the idea of “enhanced RP model” and ArchR’s manuscript and what is the unique benefit MAESTRO can provide compared to ArchR.

**Authors Response**

**Point-by-point responses to the reviewers’ comments:**

*Reviewer #1: The authors have performed a more comprehensive analysis and added some nice new functions to MAESTRO. However, it is a little bit strange that they have changed their default regulatory potential model to a different one, which is not asked by the reviewers. Additionally, the changes make their new “enhanced RP model” very similar to the best model (model 42) proposed by a recently released work named ArchR* [*https://www.biorxiv.org/content/10.1101/2020.04.28.066498v1*](https://www.biorxiv.org/content/10.1101/2020.04.28.066498v1) *from William J. Greenleaf’s lab. Since the regulatory potential model is the key to the integrative analyses of single-cell RNA-seq and ATAC-seq data, I believe it is necessary for the authors to make some clarification carefully.*

*ArchR has benchmarked 56 different models for inferring gene expression from scATAC-seq data using matched scATAC-seq and scRNA-seq data. And here is the description of their best model, “The most accurate model across both datasets was Model 42 (a model within the gene body extended + exponential decay + gene boundary class of models) (Fig. 2b) which integrates the signal from the entire gene body, and scales signal with bi-directional exponential decays from the gene TSS (extended upstream by 5 kb) and the gene transcription termination site (TTS) while accounting for neighboring genes boundaries (Fig. 2c). This model yielded robust genome-wide gene score predictions in both PBMC and bone marrow cell datasets (Fig. 2d-f and Supplementary Fig. 9i- j). We additionally confirmed the efficacy of this class of gene score models using previously 8 published paired bulk ATAC-seq and RNA-seq data from hematopoietic cells (Supplementary Fig. 9k-m)19. Given this analysis, we implemented this class of gene score models via Model 42 for all downstream analyses involving inferred gene expression in ArchR.”*

*The major difference between the MAESTRO ‘s “enhanced RP model” and ArchR’s Model 42 is that the “enhanced RP model” only has high weight for exon peaks. However, this is also suspicious. To my knowledge, there is no single literature has mentioned that ATAC peaks are enriched in exon regions. If the “enhanced RP model” also give high weight for intron peaks, then it will be almost the same as ArchR’s Model 42.*

*I believe the regulatory potential model, referred to as the “simple RP model” in the R2 version manuscript, is an original idea from MAESTRO. However, actually, it is very similar to Model 21 mentioned by ArchR and it has been shown to has poor performance compared to Model 42 in ArchR’s systematic comparative analysis. On the other hand, it is hard to tell whether the new “enhanced RP model” is “partially” inspired by ArchR or independently discovered by MAESTRO’s authors. Since the ArchR is only online in bioRxiv and has not been officially published, it is maybe not reasonable to ask the authors to include ArchR to do more comparative analysis. But it will be very important for the authors to cite ArchR’s bioRxiv manuscript in the main text and discuss what is the relationship between the idea of “enhanced RP model” and ArchR’s manuscript and what is the unique benefit MAESTRO can provide compared to ArchR.*

In the course of our investigation we observed that the gene body quantification method used by SNAP-ATAC correlates reasonably well with gene expression. We noticed, however, that much of the gene body ATAC-seq signal comes from exons, including alternative transcription start sites and sites of polymerase II pausing, and less from introns. We found that addition of the transcriptionally derived component of the ATAC-seq signal to our exponential decay regulatory component could perform better than the regulatory component alone. We exclude the exonic component of neighboring genes because we assume this signal is a result of transcription, and less likely to be associated with regulation. As this enhanced model performs better than the exponential regulatory model alone, we include it in the current release of MAESTRO. As the reviewer suggests, there are some similarities between the enhanced model and Model 42 from ArchR, and perhaps comparable performance. ArchR Model 42 is a combination of the regulatory potential model we have previously studied in the context of transcriptional regulation (Wang et al, Nature Protocols, 2012, Wang et al, Genome Research, 2016; Qin et al, Genome Biology, 2020, Chen et al, Nature Communications, 2020), combined with gene body signal, but with boundaries imposed based on the TSSs of neighboring genes. The rationale in ArchR for excluding all signal beyond the TSS of neighboring genes is unclear and is likely to miss enhancer activity that is known to not be strictly associated with the nearest gene. In MAESTRO, instead of giving equal weight to the whole gene body, only the exonic regions are considered in the transcriptional component of the model, and the intronic regions are still modeled as distal enhancers and weighted by the exponential decay function. In MAESTRO we also do not exclude all ATAC-seq signal beyond the TSSs of neighboring genes, only the exonic signal. We have discussed this in the revised manuscript and have added the citation for ArchR.
